# Supplementary material for: A CRISPRi-based genetic resource to study essential Staphylococcus aureus genes
Source: mBio. 2023 Dec 6;15(1):e02773-23. doi: 10.1128/mbio.02773-23 (PMC10870820; doi:10.1128/mbio.02773-23)
Supplement: Supplemental material — Supplemental figures and tables. [file mbio.02773-23-s0001.docx]

**SUPPLEMENTARY INFORMATION**

**A CRISPRi-based genetic resource to study essential *Staphylococcus aureus* genes**

Patricia Reed^1,#^, Moritz Sorg^1,#^, Dominik Alwardt^1,#^, Lúcia Serra^1^, Helena Veiga^1^, Simon Schäper^1^, Mariana G. Pinho^1,*^

^1^Bacterial Cell Biology, Instituto de Tecnologia Química e Biológica António Xavier, Universidade Nova de Lisboa, Oeiras, Portugal.

^#^These authors have contributed equally to this work

*Correspondence and requests for materials should be addressed to M.G.P. (email: [mgpinho@itqb.unl.pt](mailto:mgpinho@itqb.unl.pt))

[**Supplementary Figures** 2](#_Toc147509097)

[**Supplementary Table 1. Essential operons identified in *Staphylococcus aureus*** 10](#_Toc147509098)

[**Supplementary Table 2. Lisbon CRISPRi Mutant Library primer list** 22](#_Toc147509099)

[**Supplementary Table 3. Lisbon CRISPRi Mutant Library strain list** 35](#_Toc147509100)

[**Supplementary Table 4. Lisbon CRISPRi Mutant Library clone list** 44](#_Toc147509101)

[**Supplementary Table 5. Additional primers used in this study** 55](#_Toc147509102)

# **Supplementary Figures**

**Figure S1.** **Plasmid-based CRISPRi system allows leaky production of dCas9 in the absence of inducer.** (**A**) Western Blot using anti-Cas9 antibody of protein extracts from non-induced (-) and induced (+, 0.1 µM CdCl_2_) strain BCBMS02 showing that production of dCas9-sGFP increases in the presence of CdCl_2_, but is also present in the absence of the inducer. (**B**) Strains BCBMS26 and BCBMS11 showed depletion of eqFP650-RodZ even when *dCas9* expression was not induced, indicating leakiness of the cadmium-inducible promoter. The control strain lacking dCas9 (BCBMS07) shows eqFP650-RodZ localized at the septum.

**Figure S2.** **Disruption of the *rodZ* gene and expression of dCas9_spy_ do not cause growth defects in NCTC8325-4.** Overnight cultures were diluted 1:1000 in fresh media and growth was followed at 37^o^C in a 96-well plate. Disruption of the *rodZ* gene in strain NCTCΔ*rodZ* does not impair growth compared to the parental strain NCTC8325-4. Expression of dCas9_spy_ from pCNX in strain BCBMS25 (NCTC8325-4 Δ*rodZ:fp650rodZ* pBCB40*)* induced with 0.1 mM CdCl_2_ also did not affect growth.


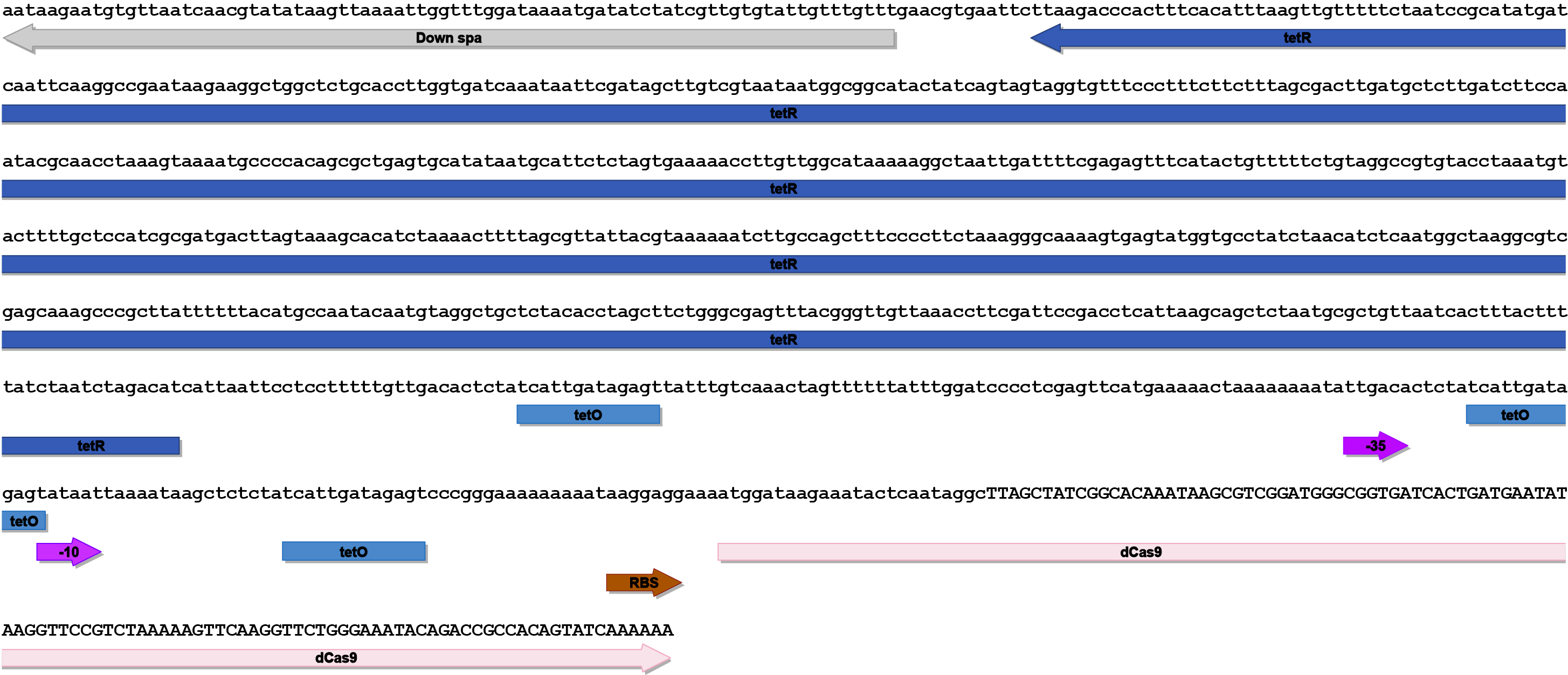


**Figure S3.** **Nucleotide sequence of P*_xyl/tetO3_* and *tetR* gene introduced in the *spa* locus of strains BCBMS14/15.** The promoter is based on the tetracycline-inducible *xyl/tetO* promoter with two additional *tetO* sites. In addition, a weak ribosome binding site (RBS) was used to reduce translation of *dCas9*.

**Figure S4. CRISPRi system with chromosome-encoded *dCas9_Spy_* is suitable to target essential genes in *S. aureus* strain COL.** Growth curves of strain BCBMS15 or the same strain expressing sgRNAs targeting the essential genes *ftsZ* (BCBMS20), *pbpA* (BCBMS 21) and *murJ* (BCBMS 22) in the absence (-) or the presence (+) of 100 ng/ml aTc (inducer for *dcas9* expression).

**Figure S5.** **Evaluation of growth inhibition in mutants from the Lisbon CRISPRi Mutant Library.** Graphs show values of R (ratio of area under the curve of growth curves obtained in the presence versus the absence of inducer aTc) for the 261 strains of the LCML containing sgRNAs targeting a predicted essential gene/operon. For each gene/operon data shown corresponds to the strain containing the sgRNA, A or B, with highest efficiency. Error bars represent SEM from three independent experiments.

**Figure S5 (continued)**

**Figure S5 (continued)**

**Figure S6.** **Evaluation of growth inhibition at 37 ^o^C in selected mutants from the Lisbon CRISPRi Mutant Library.** Growth in the presence and absence of aTc of LCML strains that showed R higher than 0.5 when growth inhibition was tested at 30 ^o^C (see Figure S5) was also evaluated at 37 ^o^C**.** R is ratio of the area under the curve of the growth curve obtained in the presence versus the absence of inducer aTc. Error bars represent SEM from three independent experiments.

# **Supplementary Table 1. Essential operons identified in *Staphylococcus aureus***

| **Operon**  **Number** | **Gene position**  **in operon** | **Locus Tag** | **Gene Name** |
| --- | --- | --- | --- |
| **1** | 1 | SAUSA300_0001 | ***dnaA*** |
| **2** | 1 | SAUSA300_0002 | ***dnaN*** |
| **3** | 1 | SAUSA300_0003 | ***0003*** |
| **3** | 2 | SAUSA300_0004 | *recF* |
| **3** | 3 | SAUSA300_0005 | ***gyrB*** |
| **3** | 4 | SAUSA300_0006 | *gyrA* |
| **4** | 1 | SAUSA300_0009 | ***serS*** |
| **5** | 1 | SAUSA300_0013 | *0013* |
| **5** | 2 | SAUSA300_0014 | ***gdpP*** |
| **5** | 3 | SAUSA300_0015 | ***rplI*** |
| **5** | 4 | SAUSA300_0016 | ***dnaB*** |
| **6** | 1 | SAUSA300_0020 | ***walR*** |
| **6** | 2 | SAUSA300_0021 | *walK* |
| **6** | 3 | SAUSA300_0022 | *walH* |
| **6** | 4 | SAUSA300_0023 | *walI* |
| **7** | 1 | SAUSA300_0024 | ***walJ*** |
| **8** | 1 | SAUSA300_0089 | ***0089*** |
| **9** | 1 | SAUSA300_0248 | ***tarF*** |
| **10** | 1 | SAUSA300_0249 | ***tarI / ispD*** |
| **10** | 2 | SAUSA300_0250 | *tarJ* |
| **10** | 3 | SAUSA300_0251 | *tarL* |
| **10** | 4 | SAUSA300_0252 | *tarS* |
| **11** | 1 | SAUSA300_0363 | ***0363*** |
| **12** | 1 | SAUSA300_0366 | ***rpsF*** |
| **12** | 2 | SAUSA300_0367 | ***ssb*** |
| **12** | 3 | SAUSA300_0368 | *rpsR* |
| **13** | 1 | SAUSA300_0386 | ***xpt*** |
| **13** | 2 | SAUSA300_0387 | *pbuX* |
| **13** | 3 | SAUSA300_0388 | ***guaB*** |
| **13** | 4 | SAUSA300_0389 | *guaA* |
| **14** | 1 | SAUSA300_0425 | ***mpsA*** |
| **14** | 2 | SAUSA300_0426 | *mpsB* |
| **15** | 1 | SAUSA300_0452 | ***dnaX*** |
| **16** | 1 | SAUSA300_0453 | ***0453*** |
| **16** | 2 | SAUSA300_0454 | *recR* |
| **17** | 1 | SAUSA300_0458 | *0458* |
| **17** | 2 | SAUSA300_0459 | ***tmk*** |
| **17** | 3 | SAUSA300_0460 | *pstA* |
| **18** | 1 | SAUSA300_0461 | ***holB*** |
| **18** | 2 | SAUSA300_0462 | *0462* |
| **18** | 3 | SAUSA300_0463 | *0463* |
| **19** | 1 | SAUSA300_0467 | ***metS*** |
| **19** | 2 | SAUSA300_0468 | *0468* |
| **Operon**  **Number** | **Gene position**  **in operon** | **Locus Tag** | **Gene Name** |
| **20** | 1 | SAUSA300_0477 | ***glmU*** |
| **21** | 1 | SAUSA300_0478 | ***prs*** |
| **22** | 1 | SAUSA300_0479 | ***rplY*** |
| **22** | 2 | SAUSA300_0480 | ***pth*** |
| **22** | 3 | SAUSA300_0481 | *mfd* |
| **22** | 4 | SAUSA300_0482 | *0482* |
| **22** | 5 | SAUSA300_0483 | *0483* |
| **22** | 6 | SAUSA300_0484 | ***0484*** |
| **22** | 7 | SAUSA300_0485 | ***divIC*** |
| **23** | 1 | SAUSA300_0487 | ***tilS*** |
| **24** | 1 | SAUSA300_0492 | ***folP*** |
| **24** | 2 | SAUSA300_0493 | *folB* |
| **24** | 3 | SAUSA300_0494 | *folK* |
| **25** | 1 | SAUSA300_0496 | ***lysS*** |
| **26** | 1 | SAUSA300_0513 | ***gltX*** |
| **27** | 1 | SAUSA300_0514 | ***cysE*** |
| **27** | 2 | SAUSA300_0515 | *cysS* |
| **27** | 3 | SAUSA300_0516 | *0516* |
| **27** | 4 | SAUSA300_0517 | *0517* |
| **27** | 5 | SAUSA300_0518 | *0518* |
| **28** | 1 | SAUSA300_0522 | ***rplK*** |
| **28** | 2 | SAUSA300_0523 | *rplA* |
| **29** | 1 | SAUSA300_0524 | ***rplJ*** |
| **29** | 2 | SAUSA300_0525 | *rplL* |
| **30** | 1 | SAUSA300_0527 | ***rpoB*** |
| **30** | 2 | SAUSA300_0528 | *rpoC* |
| **31** | 1 | SAUSA300_0529 | ***rplGB*** |
| **31** | 2 | SAUSA300_0530 | ***rpsL*** |
| **31** | 3 | SAUSA300_0531 | *0531* |
| **31** | 4 | SAUSA300_0532 | ***fusA*** |
| **31** | 5 | SAUSA300_0533 | ***tuf*** |
| **32** | 1 | SAUSA300_0553 | *0553* |
| **32** | 2 | SAUSA300_0552 | *0552* |
| **32** | 3 | SAUSA300_0551 | ***folE2*** |
| **33** | 1 | SAUSA300_0570 | ***eutD*** |
| **33** | 2 | SAUSA300_0571 | *lipL* |
| **34** | 1 | SAUSA300_0572 | ***mvaK1*** |
| **34** | 2 | SAUSA300_0573 | *mvaD* |
| **34** | 3 | SAUSA300_0574 | *mvaK2* |
| **35** | 1 | SAUSA300_0595 | *0595* |
| **35** | 2 | SAUSA300_0596 | ***argS*** |
| **36** | 1 | SAUSA300_0623 | ***tagA*** |
| **37** | 1 | SAUSA300_0624 | ***tagH*** |
| **38** | 1 | SAUSA300_0625 | ***tagG*** |
| **38** | 2 | SAUSA300_0626 | ***tagB*** |
| **Operon**  **Number** | **Gene position**  **in operon** | **Locus Tag** | **Gene Name** |
| **38** | 3 | SAUSA300_0627 | *tagX* |
| **39** | 1 | SAUSA300_0628 | ***tagD*** |
| **40** | 1 | SAUSA300_0636 | *dhaK* |
| **40** | 2 | SAUSA300_0637 | *dhal* |
| **40** | 3 | SAUSA300_0638 | *dhaM* |
| **40** | 4 | SAUSA300_0639 | ***0639*** |
| **41** | 1 | SAUSA300_0703 | ***ltaS*** |
| **42** | 1 | SAUSA300_0715 | ***nrdI*** |
| **42** | 2 | SAUSA300_0716 | *nrdE* |
| **42** | 3 | SAUSA300_0717 | *nrdF* |
| **43** | 1 | SAUSA300_0722 | ***murB*** |
| **44** | 1 | SAUSA300_0731 | ***tagO*** |
| **45** | 1 | SAUSA300_0737 | ***secA*** |
| **46** | 1 | SAUSA300_0738 | ***prfB*** |
| **47** | 1 | SAUSA300_0743 | ***hprK*** |
| **47** | 2 | SAUSA300_0744 | *lgt* |
| **47** | 3 | SAUSA300_0745 | *0745* |
| **47** | 4 | SAUSA300_0746 | *0746* |
| **47** | 5 | SAUSA300_0747 | *trxB* |
| **48** | 1 | SAUSA300_0748 | *0748* |
| **48** | 2 | SAUSA300_0749 | ***0749*** |
| **48** | 3 | SAUSA300_0750 | *0750* |
| **49** | 1 | SAUSA300_0755 | ***gapR*** |
| **49** | 2 | SAUSA300_0756 | ***gapA*** |
| **49** | 3 | SAUSA300_0757 | *pgk* |
| **49** | 4 | SAUSA300_0758 | *tpiA* |
| **49** | 5 | SAUSA300_0759 | *pgm* |
| **49** | 6 | SAUSA300_0760 | *eno* |
| **50** | 1 | SAUSA300_0761 | ***0761*** |
| **51** | 1 | SAUSA300_0763 | *est* |
| **51** | 2 | SAUSA300_0764 | *rnr* |
| **51** | 3 | SAUSA300_0765 | ***smpB*** |
| **52** | 1 | SAUSA300_0818 | ***sufC*** |
| **52** | 2 | SAUSA300_0819 | *sufD* |
| **52** | 3 | SAUSA300_0820 | *sufS* |
| **52** | 4 | SAUSA300_0821 | *sufU* |
| **52** | 5 | SAUSA300_0822 | *sufB* |
| **53** | 1 | SAUSA300_0835 | ***dltA*** |
| **53** | 2 | SAUSA300_0836 | *dltB* |
| **53** | 3 | SAUSA300_0837 | *dltC* |
| **53** | 4 | SAUSA300_0838 | *dltD* |
| **54** | 1 | SAUSA300_0858 | ***0858*** |
| **55** | 1 | SAUSA300_0865 | ***pgi*** |
| **56** | 1 | SAUSA300_0866 | *0866* |
| **56** | 2 | SAUSA300_0867 | *spsA* |
| **Operon**  **Number** | **Gene position**  **in operon** | **Locus Tag** | **Gene Name** |
| **56** | 3 | SAUSA300_0868 | ***spsB*** |
| **57** | 1 | SAUSA300_0885 | ***fabH*** |
| **57** | 2 | SAUSA300_0886 | *fabF* |
| **58** | 1 | SAUSA300_0897 | ***trpS*** |
| **59** | 1 | SAUSA300_0898 | ***spxA*** |
| **60** | 1 | SAUSA300_0906 | ***0906*** |
| **60** | 2 | SAUSA300_0907 | *relQ* |
| **60** | 3 | SAUSA300_0908 | ***ppnK*** |
| **60** | 4 | SAUSA300_0909 | *0909* |
| **60** | 5 | SAUSA300_0910 | *mgtE* |
| **60** | 6 | SAUSA300_0911 | *cpaA* |
| **61** | 1 | SAUSA300_0912 | ***fabI*** |
| **62** | 1 | SAUSA300_0919 | ***murE*** |
| **62** | 2 | SAUSA300_0920 | *0920* |
| **62** | 3 | SAUSA300_0921 | *prfC* |
| **63** | 1 | SAUSA300_0922 | ***0922*** |
| **64** | 1 | SAUSA300_0944 | ***menA*** |
| **65** | 1 | SAUSA300_0945 | *0945* |
| **65** | 2 | SAUSA300_0946 | *menD* |
| **65** | 3 | SAUSA300_0947 | *0947* |
| **65** | 4 | SAUSA300_0948 | ***menB*** |
| **66** | 1 | SAUSA300_0983 | ***ptsH*** |
| **66** | 2 | SAUSA300_0984 | *ptsI* |
| **67** | 1 | SAUSA300_0989 | ***rnjA*** |
| **67** | 2 | SAUSA300_0990 | ***rpoY*** |
| **68** | 1 | SAUSA300_0991 | ***def*** |
| **69** | 1 | SAUSA300_1013 | ***ftsW*** |
| **70** | 1 | SAUSA300_1023 | *rsmD* |
| **70** | 2 | SAUSA300_1024 | ***coaD*** |
| **71** | 1 | SAUSA300_1026 | ***1026*** |
| **71** | 2 | SAUSA300_1027 | ***rpmF*** |
| **72** | 1 | SAUSA300_1037 | ***pheS*** |
| **72** | 2 | SAUSA300_1038 | *pheT* |
| **73** | 1 | SAUSA300_1044 | ***trxA*** |
| **74** | 1 | SAUSA300_1049 | ***murI*** |
| **74** | 2 | SAUSA300_1050 | *1050* |
| **74** | 3 | SAUSA300_1051 | *1051* |
| **75** | 1 | SAUSA300_1072 | ***mraZ*** |
| **75** | 2 | SAUSA300_1073 | *mraW* |
| **75** | 3 | SAUSA300_1074 | ***ftsL*** |
| **75** | 4 | SAUSA300_1075 | ***pbpA*** |
| **76** | 1 | SAUSA300_1076 | ***mraY*** |
| **76** | 2 | SAUSA300_1077 | ***murD*** |
| **76** | 3 | SAUSA300_1078 | ***divIB*** |
| **76** | 4 | SAUSA300_1079 | ***ftsA*** |
| **Operon**  **Number** | **Gene position**  **in operon** | **Locus Tag** | **Gene Name** |
| **76** | 5 | SAUSA300_1080 | ***ftsZ*** |
| **77** | 1 | SAUSA300_1081 | *1081* |
| **77** | 2 | SAUSA300_1082 | ***1082*** |
| **77** | 3 | SAUSA300_1083 | ***sepF*** |
| **77** | 4 | SAUSA300_1084 | *1084* |
| **77** | 5 | SAUSA300_1085 | *1085* |
| **77** | 6 | SAUSA300_1086 | *1086* |
| **78** | 1 | SAUSA300_1087 | ***ileS*** |
| **79** | 1 | SAUSA300_1102 | ***gmk*** |
| **79** | 2 | SAUSA300_1103 | *rpoZ* |
| **80** | 1 | SAUSA300_1104 | ***coaBC*** |
| **80** | 2 | SAUSA300_1105 | ***priA*** |
| **81** | 1 | SAUSA300_1108 | ***def*** |
| **81** | 2 | SAUSA300_1109 | ***fmt*** |
| **81** | 3 | SAUSA300_1110 | *sun* |
| **81** | 4 | SAUSA300_1111 | *rlmN* |
| **81** | 5 | SAUSA300_1112 | *stpI* |
| **81** | 6 | SAUSA300_1113 | *pknB* |
| **82** | 1 | SAUSA300_1114 | *rsgA* |
| **82** | 2 | SAUSA300_1115 | ***rpe*** |
| **82** | 3 | SAUSA300_1116 | *thiN* |
| **83** | 1 | SAUSA300_1117 | ***rpmB*** |
| **84** | 1 | SAUSA300_1121 | ***fapR*** |
| **84** | 2 | SAUSA300_1122 | ***plsX*** |
| **84** | 3 | SAUSA300_1123 | *fabD* |
| **84** | 4 | SAUSA300_1124 | *fabG* |
| **85** | 1 | SAUSA300_1125 | ***acpP*** |
| **86** | 1 | SAUSA300_1127 | *smc* |
| **86** | 2 | SAUSA300_1128 | ***ftsY*** |
| **86** | 3 | SAUSA300_1129 | *1129* |
| **86** | 4 | SAUSA300_1130 | *ffh* |
| **87** | 1 | SAUSA300_1131 | ***rpsP*** |
| **87** | 2 | SAUSA300_1132 | *rimM* |
| **87** | 3 | SAUSA300_1133 | ***trmD*** |
| **87** | 4 | SAUSA300_1134 | ***rplS*** |
| **88** | 1 | SAUSA300_1136 | ***rbgA*** |
| **88** | 2 | SAUSA300_1137 | *rnhB* |
| **89** | 1 | SAUSA300_1142 | *dprA* |
| **89** | 2 | SAUSA300_1143 | ***topA*** |
| **90** | 1 | SAUSA300_1149 | ***rpsB*** |
| **90** | 2 | SAUSA300_1150 | ***tsf*** |
| **91** | 1 | SAUSA300_1151 | ***pyrH*** |
| **91** | 2 | SAUSA300_1152 | ***frr*** |
| **92** | 1 | SAUSA300_1153 | ***uppS*** |
| **92** | 2 | SAUSA300_1154 | *cdsA* |
| **Operon**  **Number** | **Gene position**  **in operon** | **Locus Tag** | **Gene Name** |
| **93** | 1 | SAUSA300_1155 | ***rasP*** |
| **93** | 2 | SAUSA300_1156 | ***proS*** |
| **94** | 1 | SAUSA300_1157 | ***polC*** |
| **95** | 1 | SAUSA300_1158 | ***rimP*** |
| **95** | 2 | SAUSA300_1159 | ***nusA*** |
| **95** | 3 | SAUSA300_1160 | *1160* |
| **95** | 4 | SAUSA300_1161 | *1161* |
| **95** | 5 | SAUSA300_1162 | *infB* |
| **96** | 1 | SAUSA300_1166 | ***rpsO*** |
| **97** | 1 | SAUSA300_1168 | ***rnjB*** |
| **98** | 1 | SAUSA300_1169 | *ftsK* |
| **98** | 2 | SAUSA300_1170 | *1170* |
| **98** | 3 | SAUSA300_1171 | *1171* |
| **98** | 4 | SAUSA300_1172 | *1172* |
| **98** | 5 | SAUSA300_1173 | *1173* |
| **98** | 6 | SAUSA300_1174 | *1174* |
| **98** | 7 | SAUSA300_1175 | ***rodZ*** |
| **98** | 8 | SAUSA300_1176 | ***pgsA*** |
| **99** | 1 | SAUSA300_1200 | ***glnR*** |
| **99** | 2 | SAUSA300_1201 | *glnA* |
| **100** | 1 | SAUSA300_1237 | ***lexA*** |
| **101** | 1 | SAUSA300_1239 | ***tkt*** |
| **102** | 1 | SAUSA300_1249 | ***plsY*** |
| **103** | 1 | SAUSA300_1250 | ***parE*** |
| **103** | 2 | SAUSA300_1251 | *parC* |
| **104** | 1 | SAUSA300_1257 | ***msrR*** |
| **105** | 1 | SAUSA300_1269 | ***femA*** |
| **105** | 2 | SAUSA300_1270 | ***femB*** |
| **106** | 1 | SAUSA300_1312 | *1312* |
| **106** | 2 | SAUSA300_1311 | ***murG*** |
| **106** | 3 | SAUSA300_1310 | *1310* |
| **107** | 1 | SAUSA300_1319 | ***folA*** |
| **107** | 2 | SAUSA300_1318 | *1318* |
| **108** | 1 | SAUSA300_1320 | ***thyA*** |
| **109** | 1 | SAUSA300_1340 | ***recU*** |
| **109** | 2 | SAUSA300_1341 | ***pbp2*** |
| **110** | 1 | SAUSA300_1344 | ***dnaD*** |
| **110** | 2 | SAUSA300_1343 | *nth* |
| **110** | 3 | SAUSA300_1342 | *1342* |
| **111** | 1 | SAUSA300_1349 | *1349* |
| **111** | 2 | SAUSA300_1348 | ***papS*** |
| **111** | 3 | SAUSA300_1347 | ***birA*** |
| **111** | 4 | SAUSA300_1346 | *1346* |
| **112** | 1 | SAUSA300_1354 | *1354* |
| **112** | 2 | SAUSA300_1353 | *1353* |
| **Operon**  **Number** | **Gene position**  **in operon** | **Locus Tag** | **Gene Name** |
| **112** | 3 | SAUSA300_1352 | *1352* |
| **112** | 4 | SAUSA300_1351 | ***1351*** |
| **113** | 1 | SAUSA300_1360 | ***ubiE*** |
| **113** | 2 | SAUSA300_1359 | ***1359*** |
| **114** | 1 | SAUSA300_1362 | ***hup*** |
| **115** | 1 | SAUSA300_1364 | ***engA*** |
| **115** | 2 | SAUSA300_1363 | *gpsA* |
| **116** | 1 | SAUSA300_1367 | ***cmk*** |
| **116** | 2 | SAUSA300_1366 | *1366* |
| **117** | 1 | SAUSA300_1373 | ***fer*** |
| **118** | 1 | SAUSA300_1453 | ***rnz*** |
| **119** | 1 | SAUSA300_1454 | ***zwf*** |
| **120** | 1 | SAUSA300_1460 | *1460* |
| **120** | 2 | SAUSA300_1459 | ***gnd*** |
| **121** | 1 | SAUSA300_1467 | *ipdA* |
| **121** | 2 | SAUSA300_1466 | ***bfmBAA*** |
| **121** | 3 | SAUSA300_1465 | *1465* |
| **121** | 4 | SAUSA300_1464 | ***bmfBB*** |
| **122** | 1 | SAUSA300_1476 | ***accB*** |
| **122** | 2 | SAUSA300_1475 | ***accC*** |
| **122** | 3 | SAUSA300_1474 | *1474* |
| **122** | 4 | SAUSA300_1473 | *nusB* |
| **122** | 5 | SAUSA300_1472 | *xseA* |
| **122** | 6 | SAUSA300_1471 | *xseB* |
| **122** | 7 | SAUSA300_1470 | *1470* |
| **123** | 1 | SAUSA300_1491 | *pepQ2* |
| **123** | 2 | SAUSA300_1490 | ***efp*** |
| **124** | 1 | SAUSA300_1492 | ***1492*** |
| **124** | 2 | SAUSA300_1493 | *1493* |
| **125** | 1 | SAUSA300_1511 | ***rpmG*** |
| **126** | 1 | SAUSA300_1520 | ***trmK*** |
| **126** | 2 | SAUSA300_1519 | *1519* |
| **127** | 1 | SAUSA300_1521 | ***rpoD*** |
| **128** | 1 | SAUSA300_1522 | ***dnaG*** |
| **129** | 1 | SAUSA300_1525 | ***glyS*** |
| **130** | 1 | SAUSA300_1531 | *phoH* |
| **130** | 2 | SAUSA300_1530 | ***ybeY*** |
| **130** | 3 | SAUSA300_1529 | *dgcA* |
| **130** | 4 | SAUSA300_1528 | *cdd* |
| **130** | 5 | SAUSA300_1527 | *era* |
| **130** | 6 | SAUSA300_1526 | *recO* |
| **131** | 1 | SAUSA300_1535 | ***rpsU*** |
| **132** | 1 | SAUSA300_1542 | *hrcA* |
| **132** | 2 | SAUSA300_1541 | ***grpE*** |
| **132** | 3 | SAUSA300_1540 | ***dnaK*** |
| **Operon**  **Number** | **Gene position**  **in operon** | **Locus Tag** | **Gene Name** |
| **132** | 4 | SAUSA300_1539 | *dnaJ* |
| **132** | 5 | SAUSA300_1538 | *prmA* |
| **132** | 6 | SAUSA300_1537 | *rsmE* |
| **132** | 7 | SAUSA300_1536 | *mtaB* |
| **133** | 1 | SAUSA300_1545 | ***rpsT*** |
| **134** | 1 | SAUSA300_1548 | *comEB* |
| **134** | 2 | SAUSA300_1547 | *comEC* |
| **134** | 3 | SAUSA300_1546 | ***holA*** |
| **135** | 1 | SAUSA300_1558 | ***mtnN*** |
| **135** | 2 | SAUSA300_1557 | ***1557*** |
| **135** | 3 | SAUSA300_1556 | *1556* |
| **135** | 4 | SAUSA300_1555 | *aroE* |
| **135** | 5 | SAUSA300_1554 | *1554* |
| **135** | 6 | SAUSA300_1553 | *nadD* |
| **135** | 7 | SAUSA300_1552 | *1552* |
| **135** | 8 | SAUSA300_1551 | *1551* |
| **135** | 9 | SAUSA300_1550 | *1550* |
| **135** | 10 | SAUSA300_1549 | *1549* |
| **136** | 1 | SAUSA300_1571 | *1571* |
| **136** | 2 | SAUSA300_1570 | *1570* |
| **136** | 3 | SAUSA300_1569 | *1569* |
| **136** | 4 | SAUSA300_1568 | *udk* |
| **136** | 5 | SAUSA300_1567 | ***greA*** |
| **137** | 1 | SAUSA300_1575 | ***alaS*** |
| **137** | 2 | SAUSA300_1574 | *1574* |
| **137** | 3 | SAUSA300_1573 | *1573* |
| **137** | 4 | SAUSA300_1572 | *1572* |
| **138** | 1 | SAUSA300_1579 | ***iscS*** |
| **138** | 2 | SAUSA300_1578 | *mnmA* |
| **139** | 1 | SAUSA300_1587 | ***hisS*** |
| **139** | 2 | SAUSA300_1586 | *aspS* |
| **140** | 1 | SAUSA300_1590 | ***relA*** |
| **140** | 2 | SAUSA300_1589 | ***dtd*** |
| **140** | 3 | SAUSA300_1588 | *lytH* |
| **141** | 1 | SAUSA300_1593 | ***secF*** |
| **142** | 1 | SAUSA300_1600 | ***obgE*** |
| **142** | 2 | SAUSA300_1599 | *1599* |
| **142** | 3 | SAUSA300_1598 | *ruvA* |
| **142** | 4 | SAUSA300_1597 | *ruvB* |
| **142** | 5 | SAUSA300_1596 | *queA* |
| **142** | 6 | SAUSA300_1595 | *tgt* |
| **142** | 7 | SAUSA300_1594 | *yajC* |
| **143** | 1 | SAUSA300_1603 | ***rplU*** |
| **143** | 2 | SAUSA300_1602 | ***1602*** |
| **Operon**  **Number** | **Gene position**  **in operon** | **Locus Tag** | **Gene Name** |
| **143** | 3 | SAUSA300_1601 | ***rpmA*** |
| **144** | 1 | SAUSA300_1611 | ***valS*** |
| **144** | 2 | SAUSA300_1610 | *folC* |
| **145** | 1 | SAUSA300_1619 | ***hemA*** |
| **145** | 2 | SAUSA300_1618 | *hemX* |
| **145** | 3 | SAUSA300_1617 | *hemC* |
| **145** | 4 | SAUSA300_1616 | *hemD* |
| **145** | 5 | SAUSA300_1615 | *hemB* |
| **145** | 6 | SAUSA300_1614 | *hemL* |
| **146** | 1 | SAUSA300_1620 | ***engB*** |
| **147** | 1 | SAUSA300_1624 | ***1624*** |
| **147** | 2 | SAUSA300_1623 | *1623* |
| **148** | 1 | SAUSA300_1627 | ***infC*** |
| **148** | 2 | SAUSA300_1626 | *rpmI* |
| **148** | 3 | SAUSA300_1625 | ***rplT*** |
| **149** | 1 | SAUSA300_1629 | ***thrS*** |
| **150** | 1 | SAUSA300_1632 | ***nrdR*** |
| **150** | 2 | SAUSA300_1631 | ***dnaB*** |
| **150** | 3 | SAUSA300_1630 | *dnaI* |
| **151** | 1 | SAUSA300_1636 | *polA* |
| **151** | 2 | SAUSA300_1635 | ***mutM*** |
| **151** | 3 | SAUSA300_1634 | ***coaE*** |
| **152** | 1 | SAUSA300_1645 | ***pfkA*** |
| **152** | 2 | SAUSA300_1644 | *pykA* |
| **153** | 1 | SAUSA300_1647 | ***accD*** |
| **153** | 2 | SAUSA300_1646 | *accA* |
| **154** | 1 | SAUSA300_1650 | *1650* |
| **154** | 2 | SAUSA300_1649 | ***dnaE*** |
| **155** | 1 | SAUSA300_1658 | *1658* |
| **155** | 2 | SAUSA300_1657 | ***ackA*** |
| **156** | 1 | SAUSA300_1666 | ***rpsD*** |
| **157** | 1 | SAUSA300_1673 | ***plsC*** |
| **158** | 1 | SAUSA300_1675 | ***tyrS*** |
| **159** | 1 | SAUSA300_1691 | *1691* |
| **159** | 2 | SAUSA300_1690 | ***1690*** |
| **159** | 3 | SAUSA300_1689 | *1689* |
| **159** | 4 | SAUSA300_1688 | ***1688*** |
| **159** | 5 | SAUSA300_1687 | *1687* |
| **159** | 6 | SAUSA300_1686 | ***murC*** |
| **160** | 1 | SAUSA300_1700 | ***murJ*** |
| **160** | 2 | SAUSA300_1699 | *1699* |
| **160** | 3 | SAUSA300_1698 | *1698* |
| **161** | 1 | SAUSA300_1704 | ***leuS*** |
| **161** | 2 | SAUSA300_1703 | *1703* |
| **162** | 1 | SAUSA300_1729 | ***1729*** |
| **Operon**  **Number** | **Gene position**  **in operon** | **Locus Tag** | **Gene Name** |
| **163** | 1 | SAUSA300_1730 | ***metK*** |
| **164** | 1 | SAUSA300_1748 | ***1748*** |
| **165** | 1 | SAUSA300_1749 | ***1749*** |
| **166** | 1 | SAUSA300_1793 | *1793* |
| **166** | 2 | SAUSA300_1792 | *1792* |
| **166** | 3 | SAUSA300_1791 | ***cbf1*** |
| **167** | 1 | SAUSA300_1869 | ***map*** |
| **168** | 1 | SAUSA300_1873 | ***murT*** |
| **168** | 2 | SAUSA300_1872 | *gatD* |
| **169** | 1 | SAUSA300_1879 | ***dgkB*** |
| **169** | 2 | SAUSA300_1878 | *rumA* |
| **170** | 1 | SAUSA300_1882 | ***gatC*** |
| **170** | 2 | SAUSA300_1881 | ***gatA*** |
| **170** | 3 | SAUSA300_1880 | *gatB* |
| **171** | 1 | SAUSA300_1887 | *pcrB* |
| **171** | 2 | SAUSA300_1886 | ***pcrA*** |
| **171** | 3 | SAUSA300_1885 | ***ligA*** |
| **171** | 4 | SAUSA300_1884 | *1884* |
| **172** | 1 | SAUSA300_1894 | ***pncB*** |
| **172** | 2 | SAUSA300_1893 | *nadE* |
| **173** | 1 | SAUSA300_1899 | ***pncA*** |
| **173** | 2 | SAUSA300_1900 | ***ppaC*** |
| **174** | 1 | SAUSA300_1914 | ***pmtR*** |
| **174** | 2 | SAUSA300_1913 | ***pmtA*** |
| **174** | 3 | SAUSA300_1912 | *pmtB* |
| **174** | 4 | SAUSA300_1911 | ***pmtC*** |
| **174** | 5 | SAUSA300_1910 | *pmtD* |
| **175** | 1 | SAUSA300_1983 | ***groES*** |
| **175** | 2 | SAUSA300_1982 | *groEL* |
| **176** | 1 | SAUSA300_1988 | ***hld*** |
| **177** | 1 | SAUSA300_2005 | ***tsaE*** |
| **177** | 2 | SAUSA300_2004 | *tsaB* |
| **177** | 3 | SAUSA300_2003 | *rimI* |
| **177** | 4 | SAUSA300_2002 | ***gcp*** |
| **178** | 1 | SAUSA300_2031 | *2031* |
| **178** | 2 | SAUSA300_2030 | *2030* |
| **178** | 3 | SAUSA300_2029 | ***2029*** |
| **178** | 4 | SAUSA300_2028 | ***acpS*** |
| **178** | 5 | SAUSA300_2027 | *alr* |
| **179** | 1 | SAUSA300_2039 | ***ddl*** |
| **179** | 2 | SAUSA300_2038 | *murF* |
| **180** | 1 | SAUSA300_2046 | ***oxaA*** |
| **181** | 1 | SAUSA300_2056 | *2056* |
| **181** | 2 | SAUSA300_2055 | ***murA*** |
| **181** | 3 | SAUSA300_2054 | *fabZ* |
| **Operon**  **Number** | **Gene position**  **in operon** | **Locus Tag** | **Gene Name** |
| **182** | 1 | SAUSA300_2062 | ***atpF*** |
| **182** | 2 | SAUSA300_2061 | *atpH* |
| **182** | 3 | SAUSA300_2060 | *atpA* |
| **182** | 4 | SAUSA300_2059 | *atpG* |
| **182** | 5 | SAUSA300_2058 | *atpD* |
| **182** | 6 | SAUSA300_2057 | *atpC* |
| **183** | 1 | SAUSA300_2073 | *tdk* |
| **183** | 2 | SAUSA300_2072 | ***prfA*** |
| **183** | 3 | SAUSA300_2071 | *prmC* |
| **183** | 4 | SAUSA300_2070 | *2070* |
| **183** | 5 | SAUSA300_2069 | *ptpB* |
| **184** | 1 | SAUSA300_2074 | ***rpmE2*** |
| **185** | 1 | SAUSA300_2079 | ***fbaA*** |
| **186** | 1 | SAUSA300_2081 | ***pyrG*** |
| **187** | 1 | SAUSA300_2084 | ***coaA*** |
| **188** | 1 | SAUSA300_2104 | ***glmS*** |
| **189** | 1 | SAUSA300_2113 | ***dacA*** |
| **189** | 2 | SAUSA300_2112 | *ybbR* |
| **189** | 3 | SAUSA300_2111 | *glmM* |
| **190** | 1 | SAUSA300_2172 | ***rplM*** |
| **190** | 2 | SAUSA300_2171 | ***rpsI*** |
| **191** | 1 | SAUSA300_2182 | ***infA*** |
| **191** | 2 | SAUSA300_2181 | *rpmJ* |
| **191** | 3 | SAUSA300_2180 | *rpsM* |
| **191** | 4 | SAUSA300_2179 | *rpsK* |
| **191** | 5 | SAUSA300_2178 | *rpoA* |
| **191** | 6 | SAUSA300_2177 | ***rplQ*** |
| **192** | 1 | SAUSA300_2205 | ***rpsJ*** |
| **192** | 2 | SAUSA300_2204 | *rplC* |
| **192** | 3 | SAUSA300_2203 | *rplD* |
| **192** | 4 | SAUSA300_2202 | *rplW* |
| **192** | 5 | SAUSA300_2201 | *rplB* |
| **192** | 6 | SAUSA300_2200 | *rpsS* |
| **192** | 7 | SAUSA300_2199 | *rplV* |
| **192** | 8 | SAUSA300_2198 | *rpsC* |
| **192** | 9 | SAUSA300_2197 | *rplP* |
| **192** | 10 | SAUSA300_2196 | *rpmC* |
| **192** | 11 | SAUSA300_2195 | *rpsQ* |
| **192** | 12 | SAUSA300_2194 | *rplN* |
| **192** | 13 | SAUSA300_2193 | *rplX* |
| **192** | 14 | SAUSA300_2192 | *rplE* |
| **192** | 15 | SAUSA300_2191 | *rpsN* |
| **192** | 16 | SAUSA300_2190 | *rpsH* |
| **192** | 17 | SAUSA300_2189 | *rplF* |
| **192** | 18 | SAUSA300_2188 | *rplR* |
| **Operon**  **Number** | **Gene position**  **in operon** | **Locus Tag** | **Gene Name** |
| **192** | 19 | SAUSA300_2187 | *rpsE* |
| **192** | 20 | SAUSA300_2186 | *rpmD* |
| **192** | 21 | SAUSA300_2185 | ***rplO*** |
| **192** | 22 | SAUSA300_2184 | ***secY*** |
| **192** | 23 | SAUSA300_2183 | ***adK*** |
| **193** | 1 | SAUSA300_2214 | ***femX*** |
| **193** | 2 | SAUSA300_2213 | *2213* |
| **194** | 1 | SAUSA300_2238 | ***ureA*** |
| **194** | 2 | SAUSA300_2239 | *ureB* |
| **194** | 3 | SAUSA300_2240 | *ureC* |
| **194** | 4 | SAUSA300_2241 | *ureE* |
| **194** | 5 | SAUSA300_2242 | *ureF* |
| **194** | 6 | SAUSA300_2243 | *ureG* |
| **194** | 7 | SAUSA300_2244 | *ureD* |
| **195** | 1 | SAUSA300_2283 | ***rpiA*** |
| **196** | 1 | SAUSA300_2293 | *corA* |
| **196** | 2 | SAUSA300_2292 | ***fni*** |
| **197** | 1 | SAUSA300_2483 | ***mvaA*** |
| **198** | 1 | SAUSA300_2484 | ***mvaS*** |
| **199** | 1 | SAUSA300_2646 | ***trmE*** |
| **199** | 2 | SAUSA300_2645 | *gidA* |
| **199** | 3 | SAUSA300_2644 | *gidB* |
| **199** | 4 | SAUSA300_2643 | *2643* |
| **200** | 1 | SAUSA300_2647 | ***rnpA*** |
| **200** | 2 | SAUSA300_2648 | ***rpmH*** |

Genes listed in bold were targeted with specific sgRNAs in the LCML library. Those appearing in the Nebraska transposon mutant library are underlined.

# **Supplementary Table 2. Lisbon CRISPRi Mutant Library primer list**

The primers for all A and B clones tested are listed here. The A clones denote the first sgRNA designed, the B clones are sgRNAs designed when the A clone did not exhibit a reduction in R greater than 0.5. R is the ratio of the area under the curve (AUC) of growth curves obtained in the presence (+) versus the absence (-) of anhydrotetracycline.

| **LCMLN^o^** | **Plasmid** | **Primer N^o^** | **Primer Sequence** |
| --- | --- | --- | --- |
|  |  | 5846 - EcR (reverse) | ACTAGTATTATACCTAGGACTGAGCTAGC |
| 1 | psg0001_dnaA | 8276 | ATACACAAAAATAACAGCCGgttttAGAGCTAGAAATAGCAAGTTAAAATAAGGC |
| 2 | psg0002_dnaN | 8277 | TCGTTTATATATAATTATATgttttAGAGCTAGAAATAGCAAGTTAAAATAAGGC |
| 3 | psg0003 | 8278 | ACAAATTTCATTTAAAATAGgttttAGAGCTAGAAATAGCAAGTTAAAATAAGGC |
| 4 | psg0009_serS | 8279 | CAAATAATTATCATTTATTAgttttAGAGCTAGAAATAGCAAGTTAAAATAAGGC |
| 5 | psg0016_dnaB | 8280 | TCATACATTCTATCCATGAAgttttAGAGCTAGAAATAGCAAGTTAAAATAAGGC |
| 6 | psg0020_walR | 8281 | ATGTTTGTGTAAAAAATCACgttttAGAGCTAGAAATAGCAAGTTAAAATAAGGC |
| 7 | psg0024_walJ | 8282 | TGTAACTTTAGTTCATCGACgttttAGAGCTAGAAATAGCAAGTTAAAATAAGGC |
| 8 | psg0089 | 8283 | GCCAAAATAAAAAATGGACGgttttAGAGCTAGAAATAGCAAGTTAAAATAAGGC |
| 9 | psg0248_tarF | 8284 | ATATAATTACAAAAACACGTgttttAGAGCTAGAAATAGCAAGTTAAAATAAGGC |
| 10 | psg0249_ispD | 8285 | ATATAGATATAGTTGAATGGgttttAGAGCTAGAAATAGCAAGTTAAAATAAGGC |
| 11 | psg0363 | 8286 | TGATTATAAGCAGTCATAATgttttAGAGCTAGAAATAGCAAGTTAAAATAAGGC |
| 12 | psg0367_ssb | 8287 | TTTATATTTGCACCTCCTTGgttttAGAGCTAGAAATAGCAAGTTAAAATAAGGC |
| 13 | psg0388_guaB | 8288 | GGTAAAATATCAGATTGTGCgttttAGAGCTAGAAATAGCAAGTTAAAATAAGGC |
| 14 | psg0452_dnaX | 8289 | GGAAATTTTACGATTCCGTGgttttAGAGCTAGAAATAGCAAGTTAAAATAAGGC |
| 15 | psg0453 | 8290 | ATTGATTTTCAAGGAGGAAACgttttAGAGCTAGAAATAGCAAGTTAAAATAAGGC |
| 16 | psg00459_tmk | 8291 | GTTTCTCCTTTGAAAATAATgttttAGAGCTAGAAATAGCAAGTTAAAATAAGGC |
| 17 | psg0461_holB | 8292 | CCTTTTGCTTTTATATAAAAgttttAGAGCTAGAAATAGCAAGTTAAAATAAGGC |
| 18 | psg0467_metS | 8303 | AAGATTTCTATGCATTTCAAgttttAGAGCTAGAAATAGCAAGTTAAAATAAGGC |
| 19 | psg0477_glmU | 8304 | TTCAGCACCATGTCCTACGAgttttAGAGCTAGAAATAGCAAGTTAAAATAAGGC |
| 20 | psg0478_prs | 8305 | AGATTACATTAATATTACATgttttAGAGCTAGAAATAGCAAGTTAAAATAAGGC |
| 21 | psg0480_pth | 8306 | GACAAATTCATCATTGTCATgttttAGAGCTAGAAATAGCAAGTTAAAATAAGGC |
| 22 | psg0484 | 8398 | TTCTAGCCACTGCTTTAACGgttttAGAGCTAGAAATAGCAAGTTAAAATAAGGC |
| 23 | psg0485_divIC | 8399 | CAATATCATTGCGATGTTTTgttttAGAGCTAGAAATAGCAAGTTAAAATAAGGC |
| 24 | psg0487_tilS | 8400 | ACTTTTACAATTTTCAAAAAgttttAGAGCTAGAAATAGCAAGTTAAAATAAGGC |
| 25 | psg0492_folP | 8494 | TTTATTATCTTATCATTCACgttttAGAGCTAGAAATAGCAAGTTAAAATAAGGC |
| 26 | psg0496_lysS | 8495 | TAATCCTTTAGTATTCCAACgttttAGAGCTAGAAATAGCAAGTTAAAATAAGGC |
| 27 | psg0513_gltX | 8496 | TGAAGATACCCAGTTGGACTgttttAGAGCTAGAAATAGCAAGTTAAAATAAGGC |
| 28 | psg0514_cysE | 8497 | CATACAAGTTGTATTGTTAGgttttAGAGCTAGAAATAGCAAGTTAAAATAAGGC |
| 29 | psg0532_fusA | 8498 | CTGAATAAATACGATAGATAgttttAGAGCTAGAAATAGCAAGTTAAAATAAGGC |
| 30 | psg0533 _tuf | 8499 | GTGACCGATAGTACCGATATgttttAGAGCTAGAAATAGCAAGTTAAAATAAGGC |
| 31 | psg0551_folE2 | 8500 | TTAAAGTGATATGTCCAATAgttttAGAGCTAGAAATAGCAAGTTAAAATAAGGC |
| 32 | psg0570_eutD | 8501 | TAAATCTTATTAATCATTCAgttttAGAGCTAGAAATAGCAAGTTAAAATAAGGC |
| 33 | psg0572_mvaK1 | 8502 | AAAAATAATGAATCAAGTATgttttAGAGCTAGAAATAGCAAGTTAAAATAAGGC |
| 34 | psg0596_argS | 8503 | ATATCAAGATAATGAAAAAAgttttAGAGCTAGAAATAGCAAGTTAAAATAAGGC |
| 35 | psg0623_tagA | 8504 | CTTTCTTCAACAGTCATAACgttttAGAGCTAGAAATAGCAAGTTAAAATAAGGC |
| 36 | psg0624_tagH | 8505 | AATCTAGATAAATGTGAATAgttttAGAGCTAGAAATAGCAAGTTAAAATAAGGC |
| 37 | psg0625_tagG | 8674 | AAACCATAATTTGCATAACAgttttAGAGCTAGAAATAGCAAGTTAAAATAAGGC |
| 38 | psg0626_tagB | 8675 | TGCGGTTTATCAATCACTTGgttttAGAGCTAGAAATAGCAAGTTAAAATAAGGC |
| 39 | psg0628_tagD | 8676 | TCCTCATATTGTCACATCATgttttAGAGCTAGAAATAGCAAGTTAAAATAAGGC |
| 40 | psg0703_ltaS | 8677 | CTTCAAGGTAATCGTTATTAgttttAGAGCTAGAAATAGCAAGTTAAAATAAGGC |
| 41 | psg0715_nrdl | 8678 | TAAAAAATGCTTTAACAACAgttttAGAGCTAGAAATAGCAAGTTAAAATAAGGC |
| 42 | psg0722_murB | 8679 | TCAACTTTAATTTTTTCATTgttttAGAGCTAGAAATAGCAAGTTAAAATAAGGC |
| 43 | psg0731_tagO | 8680 | TTCGATATTGCAATAACAATgttttAGAGCTAGAAATAGCAAGTTAAAATAAGGC |
| 44 | psg0737_secA | 8681 | CCTTTAGCTAAAAAACTGTTgttttAGAGCTAGAAATAGCAAGTTAAAATAAGGC |
| 45 | psg0738_prfB | 8682 | GTTTGGTTATCCCAAAAATTgttttAGAGCTAGAAATAGCAAGTTAAAATAAGGC |
| 46 | psg0743_hprK | 8683 | TATTGCTTGCTTAATTTACAgttttAGAGCTAGAAATAGCAAGTTAAAATAAGGC |
| 47 | psg0749 | 8684 | TTGTCCACCCGTACAACCGAgttttAGAGCTAGAAATAGCAAGTTAAAATAAGGC |
| 48 | psg0756_gapA | 8685 | TTCAAGTATTATCTTTGCTGgttttAGAGCTAGAAATAGCAAGTTAAAATAAGGC |
| 49 | psg0761 | 8755 | CGTTAAATTAAGTAATGCTTgttttAGAGCTAGAAATAGCAAGTTAAAATAAGGC |
| 50 | psg0765_smpB | 8756 | CGATTTTCCGCTAATGTACCgttttAGAGCTAGAAATAGCAAGTTAAAATAAGGC |
| 51 | psg0818_sufC | 8757 | TATCCTCAATAGACACATGTgttttAGAGCTAGAAATAGCAAGTTAAAATAAGGC |
| 52 | psg0835_dltA | 8758 | TGTCTAACAGCAATGCTTTGgttttAGAGCTAGAAATAGCAAGTTAAAATAAGGC |
| 53 | psg0858 | 8759 | GTCTCAACAAACGCACCGTAgttttAGAGCTAGAAATAGCAAGTTAAAATAAGGC |
| 54 | psg0865_pgi | 8760 | TTTCAACAAACATTTCAAACgttttAGAGCTAGAAATAGCAAGTTAAAATAAGGC |
| 55 | psg0868_spsB | 8761 | CGCTCGCCATCTTTCAAAGTgttttAGAGCTAGAAATAGCAAGTTAAAATAAGGC |
| 56 | psg0885_fabH | 8762 | GCATTGTCAATAATCTTTTCgttttAGAGCTAGAAATAGCAAGTTAAAATAAGGC |
| 57 | psg0897_trpS | 8763 | TAGTAGGAATTCCACTAGGTgttttAGAGCTAGAAATAGCAAGTTAAAATAAGGC |
| 58 | psg0898_spxA | 8764 | TTACGGCAAGATGTGCAACTgttttAGAGCTAGAAATAGCAAGTTAAAATAAGGC |
| 59 | psg0908_ppnK | 8765 | GTTCATCATTTTATGCTTTAgttttAGAGCTAGAAATAGCAAGTTAAAATAAGGC |
| 60 | psg0912_fabI | 8766 | CCTTATAATAATTAATTTAAgttttAGAGCTAGAAATAGCAAGTTAAAATAAGGC |
| 61 | psg0919_murE | 9735 | ACTATTAAAAATAAAACATAgttttAGAGCTAGAAATAGCAAGTTAAAATAAGGC |
| 62 | psg0922 | 9220 | ACGACAAGTACCCATAAATAgttttAGAGCTAGAAATAGCAAGTTAAAATAAGGC |
| 63 | psg0944_menA | 9736 | ACGGAAGCAGTTAATGTATGgttttAGAGCTAGAAATAGCAAGTTAAAATAAGGC |
| 64 | psg0948_menB | 9737 | GTAAACGCATTGCGTACTTCgttttAGAGCTAGAAATAGCAAGTTAAAATAAGGC |
| 65 | psg0983_ptsH | 9221 | GTTTGTACTAACATTGTTGCgttttAGAGCTAGAAATAGCAAGTTAAAATAAGGC |
| 66 | psg0989_rnjA | 9286 | GCATATACACCTACTTCATTgttttAGAGCTAGAAATAGCAAGTTAAAATAAGGC |
| 67 | psg0990_rpoY | 9287 | AAACTTTAAATACTGCCATAgttttAGAGCTAGAAATAGCAAGTTAAAATAAGGC |
| 68 | psg0991_def | 9288 | GCTGCTTTTTGACGCAAAGTgttttAGAGCTAGAAATAGCAAGTTAAAATAAGGC |
| 69 | psg1013_ftsW | 9738 | CGGATAATCAATAAACTTTGgttttAGAGCTAGAAATAGCAAGTTAAAATAAGGC |
| 70 | psg1024_coaD | 9289 | GTAATGGGGTCAAAACTACCgttttAGAGCTAGAAATAGCAAGTTAAAATAAGGC |
| 71 | psg1026 | 9739 | ACCGTTTGATCAAATTCAAAgttttAGAGCTAGAAATAGCAAGTTAAAATAAGGC |
| 72 | psg1037_pheS | 9740 | AACGCAGGTTTATCTTCATTgttttAGAGCTAGAAATAGCAAGTTAAAATAAGGC |
| 73 | psg1044_trxA | 8877 | ACCGGAGCGATCATTTTACAgttttAGAGCTAGAAATAGCAAGTTAAAATAAGGC |
| 74 | psg1049_murI | 8878 | CCAGAGTCTATTACACCTATgttttAGAGCTAGAAATAGCAAGTTAAAATAAGGC |
| 75 | psg1074_ftsL | 8879 | AAACTTGTTCGTCATATGGTgttttAGAGCTAGAAATAGCAAGTTAAAATAAGGC |
| 76 | psg1075_pbp1_b | 8880 | GTCCGAATAAACCAACAAGTgttttAGAGCTAGAAATAGCAAGTTAAAATAAGGC |
| 77 | psg1076_mraY | 8881 | TTTAATGTAGGTATTAAAACgttttAGAGCTAGAAATAGCAAGTTAAAATAAGGC |
| 78 | psg1077_murD | 8882 | AATAAAAATGTATTAGTTGTgttttAGAGCTAGAAATAGCAAGTTAAAATAAGGC |
| 79 | psg1078_divIB | 8883 | GGAATACATCTAAGAAAAGAgttttAGAGCTAGAAATAGCAAGTTAAAATAAGGC |
| 80 | psg1079_ftsA | 8884 | ATTTTTTATACCGCTCGTGTgttttAGAGCTAGAAATAGCAAGTTAAAATAAGGC |
| 81 | psg1082 | 8885 | TTTGTAACTGCAATCACGTTgttttAGAGCTAGAAATAGCAAGTTAAAATAAGGC |
| 82 | psg1083_sepF | 8886 | TTTACCTGTTGTTGTTTGTCgttttAGAGCTAGAAATAGCAAGTTAAAATAAGGC |
| 83 | psg1087_ileS | 8887 | CGCATTGGGAAATCTGTTTTgttttAGAGCTAGAAATAGCAAGTTAAAATAAGGC |
| 84 | psg1102_gmk | 8888 | GTACCTTTACCTACTCCAGAgttttAGAGCTAGAAATAGCAAGTTAAAATAAGGC |
| 85 | psg1104_coaBC | 8889 | TGCCGCAATGCCACCTGTAAgttttAGAGCTAGAAATAGCAAGTTAAAATAAGGC |
| 86 | psg1105_priA | 8890 | GATGACAGATTCGAGTTGTTgttttAGAGCTAGAAATAGCAAGTTAAAATAAGGC |
| 87 | psg1109_fmt | 8891 | AAAACAGTTGTTGAAAAGTCgttttAGAGCTAGAAATAGCAAGTTAAAATAAGGC |
| 88 | psg1115_rpe | 8892 | AAATCAACAGATAATAATGAgttttAGAGCTAGAAATAGCAAGTTAAAATAAGGC |
| 89 | psg1122_plsX | 8893 | GATATCGTATTAGAAGCCGTgttttAGAGCTAGAAATAGCAAGTTAAAATAAGGC |
| 90 | psg1125_acpP | 8894 | TATCAGCGTCTACACCTAAAgttttAGAGCTAGAAATAGCAAGTTAAAATAAGGC |
| 91 | psg1128_ftsY | 8895 | TTGACCTTGTTCTTCTGTTAgttttAGAGCTAGAAATAGCAAGTTAAAATAAGGC |
| 92 | psg1133_trmD | 8896 | AAAACACCATCAAACATTTCgttttAGAGCTAGAAATAGCAAGTTAAAATAAGGC |
| 93 | psg1136_rbgA | 8897 | TTGGCTTTCGCCATATGTCCgttttAGAGCTAGAAATAGCAAGTTAAAATAAGGC |
| 94 | psg1143_topA | 8898 | TCAATGGTTTTTGCTTTTGCgttttAGAGCTAGAAATAGCAAGTTAAAATAAGGC |
| 95 | psg1150_tsf | 8899 | TAGCAATACCTTTTTCACGTgttttAGAGCTAGAAATAGCAAGTTAAAATAAGGC |
| 96 | psg1151_pyrH | 8900 | GCAACACTTTTAATAATTACgttttAGAGCTAGAAATAGCAAGTTAAAATAAGGC |
| 97 | psg1152_frr | 8981 | AATTAGCTAACATCAGTGCAgttttAGAGCTAGAAATAGCAAGTTAAAATAAGGC |
| 98 | psg1153_uppS | 8982 | ATTTATTAGCTTTTTAAACAgttttAGAGCTAGAAATAGCAAGTTAAAATAAGGC |
| 99 | psg1156_proS | 8983 | CATCGTTGGTATAAAAACTTgttttAGAGCTAGAAATAGCAAGTTAAAATAAGGC |
| 100 | psg1157_polC | 8984 | TCTTCATGAGCTAAGAATTGgttttAGAGCTAGAAATAGCAAGTTAAAATAAGGC |
| 101 | psg1159_nusA | 8985 | GCATCAATTAATACTGCTCTgttttAGAGCTAGAAATAGCAAGTTAAAATAAGGC |
| 102 | psg1168_rnjB | 8986 | TGAATAGTGAGTTTATATATgttttAGAGCTAGAAATAGCAAGTTAAAATAAGGC |
| 103 | psg1176_pgsA | 8987 | CTAAAAACCGTAATCTGGTTgttttAGAGCTAGAAATAGCAAGTTAAAATAAGGC |
| 104 | psg1200_glnR | 8988 | TGATGCAATCAGACGAAATAgttttAGAGCTAGAAATAGCAAGTTAAAATAAGGC |
| 105 | psg1237_lexA | 8989 | CCAATTTCGCGAACACTAGGgttttAGAGCTAGAAATAGCAAGTTAAAATAAGGC |
| 106 | psg1239_tkt | 8990 | TTGAAGTAATCTTTAGATTGgttttAGAGCTAGAAATAGCAAGTTAAAATAAGGC |
| 107 | psg1249_plsY | 8991 | TTTCCAATTACGAATCCACTgttttAGAGCTAGAAATAGCAAGTTAAAATAAGGC |
| 108 | psg1250_parE | 8992 | GTTGATCCAATATACATACCgttttAGAGCTAGAAATAGCAAGTTAAAATAAGGC |
| 109 | psg1257_msrR | 9018 | TTCTTCTTCTTTTTTCGCTTgttttAGAGCTAGAAATAGCAAGTTAAAATAAGGC |
| 110 | psg1269_femA | 9019 | TTACAGATAGCATGCCATACgttttAGAGCTAGAAATAGCAAGTTAAAATAAGGC |
| 111 | psg1270_femB | 9020 | TTGTACAAAGTTGTCAAATTgttttAGAGCTAGAAATAGCAAGTTAAAATAAGGC |
| 112 | psg1311_murG | 9021 | TGTCCAACTGTTCCCCCTCCgttttAGAGCTAGAAATAGCAAGTTAAAATAAGGC |
| 113 | psg1319_folA | 9022 | CAAGTCATGTGCAACTAGAAgttttAGAGCTAGAAATAGCAAGTTAAAATAAGGC |
| 114 | psg1320_thyA | 9023 | ACTTTCTTTGTCGTTAATAGgttttAGAGCTAGAAATAGCAAGTTAAAATAAGGC |
| 115 | psg1340_recU | 9024 | TTACGATATGGTTTACCATTgttttAGAGCTAGAAATAGCAAGTTAAAATAAGGC |
| 116 | psg1341_pbp2 | 9025 | CCATTATTACCGTTTTTCTTgttttAGAGCTAGAAATAGCAAGTTAAAATAAGGC |
| 117 | psg1344_dnaD | 9026 | AATTCTCTTCGTATCACTACgttttAGAGCTAGAAATAGCAAGTTAAAATAAGGC |
| 118 | psg1347_birA | 9027 | CTTTGTCCAGATATATAATTgttttAGAGCTAGAAATAGCAAGTTAAAATAAGGC |
| 119 | psg1348_papS | 9028 | TCTTGAATTTGTTCTAATATgttttAGAGCTAGAAATAGCAAGTTAAAATAAGGC |
| 120 | psg1351 | 9029 | ACTTTGTGTTGTGCCCATAAgttttAGAGCTAGAAATAGCAAGTTAAAATAAGGC |
| 121 | psg1360_ubiE | 9030 | AAAACGCGTCATGAAAGACAgttttAGAGCTAGAAATAGCAAGTTAAAATAAGGC |
| 122 | psg1362_hup | 9031 | CATTAGACATTCACCTCCTGgttttAGAGCTAGAAATAGCAAGTTAAAATAAGGC |
| 123 | psg1364_engA | 9032 | ATTGTAGATTTACCTACATTgttttAGAGCTAGAAATAGCAAGTTAAAATAAGGC |
| 124 | psg1367_cmk | 9033 | ACCATCTAATGCAATATTAAgttttAGAGCTAGAAATAGCAAGTTAAAATAAGGC |
| 125 | psg1373_fer | 9034 | TCGTCGTAATCATATATATCgttttAGAGCTAGAAATAGCAAGTTAAAATAAGGC |
| 126 | psg1453_rnz | 9035 | TGTGTATTTCTCTCTTTTGTgttttAGAGCTAGAAATAGCAAGTTAAAATAAGGC |
| 127 | psg1454_zwf | 9036 | CCAAAGATTGTGATTAAACAgttttAGAGCTAGAAATAGCAAGTTAAAATAAGGC |
| 128 | psg1459_gnd | 9037 | AGCTAGGTTTTTACCCATAAgttttAGAGCTAGAAATAGCAAGTTAAAATAAGGC |
| 129 | psg1464_bmfBB | 9038 | TGAACACTCTCACCTAACTTgttttAGAGCTAGAAATAGCAAGTTAAAATAAGGC |
| 130 | psg1466_bmfBAA | 9039 | CTTTTAGGTCTTCTTCGCTAgttttAGAGCTAGAAATAGCAAGTTAAAATAAGGC |
| 131 | psg1475_accC | 9040 | TCCTAACTGCGATTTCACCGgttttAGAGCTAGAAATAGCAAGTTAAAATAAGGC |
| 132 | psg1490_efp | 9041 | CTTTACCAGGCTTTACATGTgttttAGAGCTAGAAATAGCAAGTTAAAATAAGGC |
| 133 | psg1492 | 8530 | TCTGTTGTTTTTTCTTCTAAgttttAGAGCTAGAAATAGCAAGTTAAAATAAGGC |
| 134 | psg1520_trmK | 8531 | ATTCTCCTTTATGAAAAAAGgttttAGAGCTAGAAATAGCAAGTTAAAATAAGGC |
| 135 | psg1521_rpoD | 8532 | CTGTGTTATCAGACATGAAAgttttAGAGCTAGAAATAGCAAGTTAAAATAAGGC |
| 136 | psg1522_dnaG | 8533 | ATTTATTTTGTTCAATATAAgttttAGAGCTAGAAATAGCAAGTTAAAATAAGGC |
| 137 | psg1525_glyS | 8534 | TCATACATGAAAACGCCCCAgttttAGAGCTAGAAATAGCAAGTTAAAATAAGGC |
| 138 | psg1530_ybeY | 8535 | TCAATGATCTTACTTACCAAgttttAGAGCTAGAAATAGCAAGTTAAAATAAGGC |
| 139 | psg1540_dnaK | 8536 | TCAGGGTTTTGAATTACTTTgttttAGAGCTAGAAATAGCAAGTTAAAATAAGGC |
| 140 | psg1541_grpE | 8537 | TCATTAATTTTTTGATCTTTgttttAGAGCTAGAAATAGCAAGTTAAAATAAGGC |
| 141 | psg1546_holA | 8538 | CTTTGTTTTTCAACCAATTCgttttAGAGCTAGAAATAGCAAGTTAAAATAAGGC |
| 142 | psg1557 | 8539 | ATTGATTGAACATATGAATTgttttAGAGCTAGAAATAGCAAGTTAAAATAAGGC |
| 143 | psg1567_greA | 8540 | TCAAAACCTTCTTGAGTCATgttttAGAGCTAGAAATAGCAAGTTAAAATAAGGC |
| 144 | psg1575_alaS | 8541 | ATTGGCACTAATGGTGCAGAgttttAGAGCTAGAAATAGCAAGTTAAAATAAGGC |
| 145 | p[sg1579_iscS](javascript:void(0)) | 8738 | TACTTCAGGTTTTACTGGTGgttttAGAGCTAGAAATAGCAAGTTAAAATAAGGC |
| 146 | p[sg1587_hisS](javascript:void(0)) | 8739 | AAAATATCCTGCGTCCCTCTgttttAGAGCTAGAAATAGCAAGTTAAAATAAGGC |
| 147 | psg1589_dtd | 8740 | GTATTTCACCATTCATTTGTgttttAGAGCTAGAAATAGCAAGTTAAAATAAGGC |
| 148 | psg1593_secF | 8741 | TTTATAAGTTGCAGCCATTCgttttAGAGCTAGAAATAGCAAGTTAAAATAAGGC |
| 149 | p[sg1600_obgE](javascript:void(0)) | 8742 | AATACCATTACCACCATCACgttttAGAGCTAGAAATAGCAAGTTAAAATAAGGC |
| 150 | p[sg1602](javascript:void(0)) | 8743 | CATATTCACCATGGTCAGCAgttttAGAGCTAGAAATAGCAAGTTAAAATAAGGC |
| 151 | psg1611_valS | 8744 | ACTTCACGAGGATCATATTTgttttAGAGCTAGAAATAGCAAGTTAAAATAAGGC |
| 152 | psg1619_hemA | 8745 | ATCTTCATGGGCAATTCGTAgttttAGAGCTAGAAATAGCAAGTTAAAATAAGGC |
| 153 | psg1620_engB | 8746 | ATGATTAATTCTATATTATTgttttAGAGCTAGAAATAGCAAGTTAAAATAAGGC |
| 154 | psg1624 | 8747 | TTATTAAAAATAATTTCTCTgttttAGAGCTAGAAATAGCAAGTTAAAATAAGGC |
| 155 | psg1627_infC | 8748 | TTGAGTTTGATCTTTTGCTAgttttAGAGCTAGAAATAGCAAGTTAAAATAAGGC |
| 156 | psg1629_thrS | 8749 | AACGCCTTTTTATTACCATCgttttAGAGCTAGAAATAGCAAGTTAAAATAAGGC |
| 157 | psg1631_dnaB | 8792 | TGGTCTTAAGCCGAATTCGAgttttAGAGCTAGAAATAGCAAGTTAAAATAAGGC |
| 158 | psg1632_nrdR | 8793 | ATTGTGTAGAATTACATTTCgttttAGAGCTAGAAATAGCAAGTTAAAATAAGGC |
| 159 | psg1634_coaE | 8794 | CCTGTTAGACCAATAACTTTgttttAGAGCTAGAAATAGCAAGTTAAAATAAGGC |
| 160 | psg1645_pfkA | 8795 | TGTACGAACAACTGCTCTTAgttttAGAGCTAGAAATAGCAAGTTAAAATAAGGC |
| 161 | psg1647_accD | 8796 | CACTTAGTCATAATACCTGCgttttAGAGCTAGAAATAGCAAGTTAAAATAAGGC |
| 162 | psg1649_dnaE | 8797 | TTCAGACACAGCAAGTCTTAgttttAGAGCTAGAAATAGCAAGTTAAAATAAGGC |
| 163 | psg1657_ackA | 8798 | TTTGTTACTAATTCCTCTTCgttttAGAGCTAGAAATAGCAAGTTAAAATAAGGC |
| 164 | psg1673_plsC | 8799 | ACGACATATTTACTATCCTTgttttAGAGCTAGAAATAGCAAGTTAAAATAAGGC |
| 165 | psg1675_tyrS | 8800 | TAAACTATCTGCCGTTGGATgttttAGAGCTAGAAATAGCAAGTTAAAATAAGGC |
| 166 | psg1686_murC | 8801 | TTTATGTTATTAGCATCAAAgttttAGAGCTAGAAATAGCAAGTTAAAATAAGGC |
| 167 | psg1688 | 8802 | GCGACATCTCCTACATATTTgttttAGAGCTAGAAATAGCAAGTTAAAATAAGGC |
| 168 | psg1704_leuS | 8803 | ATTTCTTTTCAATTTGATTGgttttAGAGCTAGAAATAGCAAGTTAAAATAAGGC |
| 169 | psg1729 | 8804 | ATAATGAGTCCAATGACTATgttttAGAGCTAGAAATAGCAAGTTAAAATAAGGC |
| 170 | psg1730_metK | 8805 | CTTGGTCAGCGATTTTATCTgttttAGAGCTAGAAATAGCAAGTTAAAATAAGGC |
| 171 | psg1748 | 8806 | TCGCGTATAATTTCATTCCTgttttAGAGCTAGAAATAGCAAGTTAAAATAAGGC |
| 172 | psg1749 | 8807 | TAATAGAATAACCATCCATTgttttAGAGCTAGAAATAGCAAGTTAAAATAAGGC |
| 173 | psg1869_map | 8808 | TTTCGTAGTGATACCTGGTTgttttAGAGCTAGAAATAGCAAGTTAAAATAAGGC |
| 174 | psg1873_murT | 8809 | TACGCGCCAATTTCGCTAGAgttttAGAGCTAGAAATAGCAAGTTAAAATAAGGC |
| 175 | psg1879_dgkB | 8810 | ATAGCTCTTTACCTGATGTCgttttAGAGCTAGAAATAGCAAGTTAAAATAAGGC |
| 176 | psg1881_gatA | 8811 | ATATCTTTAACAACATCAGAgttttAGAGCTAGAAATAGCAAGTTAAAATAAGGC |
| 177 | psg1882_gatC | 8812 | GCCATTTCTTCCGTTTCTTCgttttAGAGCTAGAAATAGCAAGTTAAAATAAGGC |
| 178 | psg1885_ligA | 8813 | TATTCACTATCTGGTACAGAgttttAGAGCTAGAAATAGCAAGTTAAAATAAGGC |
| 179 | psg1886_pcrA | 8814 | GCACCTGCCATAATTAACAAgttttAGAGCTAGAAATAGCAAGTTAAAATAAGGC |
| 180 | psg1894_pncB | 8815 | TTAAACTGTCGTCTTCTAATgttttAGAGCTAGAAATAGCAAGTTAAAATAAGGC |
| 181 | psg1900_ppaC | 8970 | GATGAAATTGCATCAGTGTCgttttAGAGCTAGAAATAGCAAGTTAAAATAAGGC |
| 182 | psg1911_pmtC | 8971 | ACCATTCATTACTTTCATAAgttttAGAGCTAGAAATAGCAAGTTAAAATAAGGC |
| 183 | psg1913_pmtA | 8972 | ATTAACATTACTTAATTCTAgttttAGAGCTAGAAATAGCAAGTTAAAATAAGGC |
| 184 | psg1983_groES | 8973 | ATAATCACACGATTTCCAATgttttAGAGCTAGAAATAGCAAGTTAAAATAAGGC |
| 185 | psg1988_hld | 8974 | ACTAGATCACAGAGATGTGAgttttAGAGCTAGAAATAGCAAGTTAAAATAAGGC |
| 186 | psg2002_gcp | 8975 | TGATGTCTACTTGCCACTTCgttttAGAGCTAGAAATAGCAAGTTAAAATAAGGC |
| 187 | psg2005_tsaE | 8976 | TTTAATGATGTTAAATGTCGgttttAGAGCTAGAAATAGCAAGTTAAAATAAGGC |
| 188 | psg2028_acpS | 8977 | AAAATCCGCTCAACCAATTTgttttAGAGCTAGAAATAGCAAGTTAAAATAAGGC |
| 189 | psg2039_ddl | 8978 | CTTTCTCCAATCACCATCATgttttAGAGCTAGAAATAGCAAGTTAAAATAAGGC |
| 190 | psg2046_oxaA | 8979 | ACCATAATACCTAAAAATAAgttttAGAGCTAGAAATAGCAAGTTAAAATAAGGC |
| 191 | psg2055_murA | 8980 | AATAAAGATGCTGTCAATATgttttAGAGCTAGAAATAGCAAGTTAAAATAAGGC |
| 192 | psg2062_atpF | 8981 | TAAGTTAGCTGTTTCAGTCAgttttAGAGCTAGAAATAGCAAGTTAAAATAAGGC |
| 193 | psg2072_prfA | 9003 | CTGAATCATTTACAACATCTgttttAGAGCTAGAAATAGCAAGTTAAAATAAGGC |
| 194 | psg2079_fbaA | 9004 | ATTTCTTTCATTGAAACTAAgttttAGAGCTAGAAATAGCAAGTTAAAATAAGGC |
| 195 | psg2081_pyrG | 9005 | CCTGGGTCAACATTTAAGTAgttttAGAGCTAGAAATAGCAAGTTAAAATAAGGC |
| 196 | psg2084_coaA | 9006 | ATTCAGTTTTAAAAGTACGTgttttAGAGCTAGAAATAGCAAGTTAAAATAAGGC |
| 197 | psg2104_glmS | 9007 | ACCTTTTAATAATAATTCTTgttttAGAGCTAGAAATAGCAAGTTAAAATAAGGC |
| 198 | psg2113_dacA | 9008 | ACTGAGGTTTTGAAAAAAGTgttttAGAGCTAGAAATAGCAAGTTAAAATAAGGC |
| 199 | psg2182_infA | 9009 | AACGCAATGTTTAAAGTAGAgttttAGAGCTAGAAATAGCAAGTTAAAATAAGGC |
| 200 | psg2184_secY | 9010 | TGTTCTAAAGAAGTTCACAAgttttAGAGCTAGAAATAGCAAGTTAAAATAAGGC |
| 201 | psg2214_femX | 9011 | TAATAAATCTCCATTTGGGTgttttAGAGCTAGAAATAGCAAGTTAAAATAAGGC |
| 202 | psg2238_ureA | 9012 | CTGATTAAAGCTAATGCCTCgttttAGAGCTAGAAATAGCAAGTTAAAATAAGGC |
| 203 | psg2283_rpiA | 9013 | TTAATTAGTTGCGCCATTTGgttttAGAGCTAGAAATAGCAAGTTAAAATAAGGC |
| 204 | psg2292_fni | 9014 | TGAATGCATTGCGTCAGATTgttttAGAGCTAGAAATAGCAAGTTAAAATAAGGC |
| 205 | psg2483_mvaA | 9015 | ATTCTTATCTAAATTTTGCAgttttAGAGCTAGAAATAGCAAGTTAAAATAAGGC |
| 206 | psg2646_trmE | 9017 | CCAATTGCCCCTTCACCCATgttttAGAGCTAGAAATAGCAAGTTAAAATAAGGC |
| 207 | psg2484_mvaS | 9016 | CCATGTCTACATAGTACTTgttttAGAGCTAGAAATAGCAAGTTAAAATAAGGC |
| 208 | psg0005_gyrB | 9484 | TGATTAATACGATACAATTTgttttAGAGCTAGAAATAGCAAGTTAAAATAAGGC |
| 209 | psg0527_rpoB | 9485 | GTTTACGATGTCTTCCATATgttttAGAGCTAGAAATAGCAAGTTAAAATAAGGC |
| 210 | psg0425_mpsA | 9486 | ATAGTAGATTCTGTACATAAgttttAGAGCTAGAAATAGCAAGTTAAAATAAGGC |
| 211 | psg0639 | 9487 | TCAATACATGTTTTTTTATAgttttAGAGCTAGAAATAGCAAGTTAAAATAAGGC |
| 212 | psg1359 | 9488 | TTTGCCACGTTAATCACCTTgttttAGAGCTAGAAATAGCAAGTTAAAATAAGGC |
| 213 | psg1590_relA | 9489 | TTCGCCTTAAACAATGATTTgttttAGAGCTAGAAATAGCAAGTTAAAATAAGGC |
| 214 | psg1791_cbf1 | 9490 | AATGTAATCACTTCTTTTGAgttttAGAGCTAGAAATAGCAAGTTAAAATAAGGC |
| 215 | psg2183_adk | 9491 | TGAGTTCCTTTACCTGCGCCgttttAGAGCTAGAAATAGCAAGTTAAAATAAGGC |
| 216 | psg2185_rplO | 9492 | TAACTCATGTAATTTCATTTgttttAGAGCTAGAAATAGCAAGTTAAAATAAGGC |
| 217 | psg2647_rnpA | 9493 | AATCTGCATTCTTTTTAATTgttttAGAGCTAGAAATAGCAAGTTAAAATAAGGC |
| 218 | psg2648_rpmH | 9494 | TTACTATGTTTACGTTTATTgttttAGAGCTAGAAATAGCAAGTTAAAATAAGGC |
| 219 | psg0015_rplI | 9495 | AAGTTATTTGCATAACCTACgttttAGAGCTAGAAATAGCAAGTTAAAATAAGGC |
| 220 | psg0366_rpsF | 9496 | TTTATATTTGCACCTCCTTGgttttAGAGCTAGAAATAGCAAGTTAAAATAAGGC |
| 221 | psg0479_rplY | 9497 | AACGTGTTTGTTTACCTTGAgttttAGAGCTAGAAATAGCAAGTTAAAATAAGGC |
| 222 | psg0522_rplK | 9498 | TGATATCGTGATGTGGTCACgttttAGAGCTAGAAATAGCAAGTTAAAATAAGGC |
| 223 | psg0524_rplJ | 9499 | GACACCTCCATTTAAATTTTgttttAGAGCTAGAAATAGCAAGTTAAAATAAGGC |
| 224 | psg0530_rpsL | 9500 | CGTACTAATTGGTTAATAGTgttttAGAGCTAGAAATAGCAAGTTAAAATAAGGC |
| 225 | psg0990_rpoY | 9501 | TAACCCTAGTAAAATCGTATgttttAGAGCTAGAAATAGCAAGTTAAAATAAGGC |
| 226 | psg1027_rpmF | 9502 | GTTTTAGAAGTTCTTCTTTTgttttAGAGCTAGAAATAGCAAGTTAAAATAAGGC |
| 227 | psg1117_rpmB | 9503 | CAGTTTACTCAAAATATAATgttttAGAGCTAGAAATAGCAAGTTAAAATAAGGC |
| 228 | psg1131_rpsP | 9504 | GCTACTACGATACGATAGAAgttttAGAGCTAGAAATAGCAAGTTAAAATAAGGC |
| 229 | psg1134_rplS | 9505 | CCTCGACAAATATATAGCAGgttttAGAGCTAGAAATAGCAAGTTAAAATAAGGC |
| 230 | psg1149_rpsB | 9506 | CCATTATAAATTCCTCCTATgttttAGAGCTAGAAATAGCAAGTTAAAATAAGGC |
| 231 | psg1166_rpsO | 9507 | AATACTCCTTAATCCGAGTTgttttAGAGCTAGAAATAGCAAGTTAAAATAAGGC |
| 232 | psg1511_rpmG | 9508 | TAAAGTTACGTTTACGCGCAgttttAGAGCTAGAAATAGCAAGTTAAAATAAGGC |
| 233 | psg1535_rpsU | 9509 | TCCCTCCCTCCAAATATCAAgttttAGAGCTAGAAATAGCAAGTTAAAATAAGGC |
| 234 | psg1545_rpsT | 9510 | TTGTGCAATCATTTATTTGAgttttAGAGCTAGAAATAGCAAGTTAAAATAAGGC |
| 235 | psg1601_rpmA | 9511 | TGTAAGTTTAATTTTAACATgttttAGAGCTAGAAATAGCAAGTTAAAATAAGGC |
| 236 | psg1625_rplT | 9512 | GGCATAAAAAATTCCTCCTTgttttAGAGCTAGAAATAGCAAGTTAAAATAAGGC |
| 237 | psg1666_rpsD | 9513 | CCATGTTGTCCTGGTGCGTAgttttAGAGCTAGAAATAGCAAGTTAAAATAAGGC |
| 238 | psg2074_rpmE2 | 9514 | CTCCTTTGCCCTGAACCATCgttttAGAGCTAGAAATAGCAAGTTAAAATAAGGC |
| 239 | psg2171_rpsI | 9515 | CCACGTAATTCGTAGTTTTCgttttAGAGCTAGAAATAGCAAGTTAAAATAAGGC |
| 240 | psg2177_rplQ | 9516 | GAAAAGAAGATTGATAAAGGgttttAGAGCTAGAAATAGCAAGTTAAAATAAGGC |
| 241 | psg0386_xpt | 10048 | CCAAACTAAATAATAGTTTCgttttAGAGCTAGAAATAGCAAGTTAAAATAAGGC |
| 242 | psg0529_rplGB | 10050 | ATTTACATAAAAATAACAAGgttttAGAGCTAGAAATAGCAAGTTAAAATAAGGC |
| 243 | psg0755_gapR | 10052 | ATTTACATAAAAATAACAAGgttttAGAGCTAGAAATAGCAAGTTAAAATAAGGC |
| 244 | psg0906 | 10054 | CTTACGAACACTATTTTTAAgttttAGAGCTAGAAATAGCAAGTTAAAATAAGGC |
| 245 | psg1072_mraZ | 10056 | GATTTATCATTAAGATAAATgttttAGAGCTAGAAATAGCAAGTTAAAATAAGGC |
| 246 | psg1108_def | 10058 | GTAATCCTAAATTTATTGTAgttttAGAGCTAGAAATAGCAAGTTAAAATAAGGC |
| 247 | psg1121_fapR | 10060 | AGTTCATGGTCTGTGATGAAgttttAGAGCTAGAAATAGCAAGTTAAAATAAGGC |
| 248 | psg1155_rasP | 10062 | CCCATACCGATCGCAAATTCgttttAGAGCTAGAAATAGCAAGTTAAAATAAGGC |
| 249 | psg1158_rimP | 10064 | AAATTCAAGTCTTCCATAATgttttAGAGCTAGAAATAGCAAGTTAAAATAAGGC |
| 250 | psg1558_mtnM | 10066 | TATTGTTACTTCTTCTTCCAgttttAGAGCTAGAAATAGCAAGTTAAAATAAGGC |
| 251 | psg1635_mutM | 10068 | CTTTTTACATGTTCTACTTCgttttAGAGCTAGAAATAGCAAGTTAAAATAAGGC |
| 252 | psg1690 | 10070 | GGTTCTATCACTCTACAATCgttttAGAGCTAGAAATAGCAAGTTAAAATAAGGC |
| 253 | psg1914_pmtR | 10072 | TGCTTAATCTGTTCATAAATgttttAGAGCTAGAAATAGCAAGTTAAAATAAGGC |
| 254 | psg2029 | 10074 | CTCATCACTTTTTTAGCGTGgttttAGAGCTAGAAATAGCAAGTTAAAATAAGGC |
| 255 | psg2205_rpsJ | 10076 | CTGCTGATTGATCAATTACGgttttAGAGCTAGAAATAGCAAGTTAAAATAAGGC |
| 256 | psg0014_gdpP | 10078 | TATTAGTAAAGCTTTCTTAGgttttAGAGCTAGAAATAGCAAGTTAAAATAAGGC |
| 257 | psg1603_rplU | 10080 | TAATAAGTCACGCCATACATgttttAGAGCTAGAAATAGCAAGTTAAAATAAGGC |
| 258 | psg2171_rplM | 10082 | ATGATAAACGACCTAATGTTgttttAGAGCTAGAAATAGCAAGTTAAAATAAGGC |
| 259 | psg1476_accB | 9891 | TTCAAGCATGTTCATATTGCgttttAGAGCTAGAAATAGCAAGTTAAAATAAGGC |
| 260 | psg1700_murJ | 6423 | CTTGGTAATTAATATACTAAgttttAGAGCTAGAAATAGCAAGTTAAAATAAGGC |
| 261 | psg1080_ftsZ | 6424 | AAATTTCCTCCTAGTTTTAgttttAGAGCTAGAAATAGCAAGTTAAAATAAGGC |
|  | **B Clones** |  |  |
| 4B | psg0009_serS_B | 9062 | ATTTTGCTCTTAACTGTGTCgttttAGAGCTAGAAATAGCAAGTTAAAATAAGGC |
| 6B | psg0020_walR_B | 9063 | AATTCTAAAATATCAGCAATgttttAGAGCTAGAAATAGCAAGTTAAAATAAGGC |
| 7B | psg0024_walJ_B | 9064 | ATACACTCATGCGTATCAAGgttttAGAGCTAGAAATAGCAAGTTAAAATAAGGC |
| 8B | psg0089_B | 9065 | AATTCAGTGAAAAACACATCgttttAGAGCTAGAAATAGCAAGTTAAAATAAGGC |
| 9B | psg0248_tarF_B | 9066 | AAATAAATTAGATTCTTGTTgttttAGAGCTAGAAATAGCAAGTTAAAATAAGGC |
| 10B | psg0249_ispD_B | 9276 | TCTAATGTATGGATTAAAATgttttAGAGCTAGAAATAGCAAGTTAAAATAAGGC |
| 15B | psg0453_B | 9546 | GTTTGTTGTGCTTGTTGCTTgttttAGAGCTAGAAATAGCAAGTTAAAATAAGGC |
| 24B | psg0487_tilS_B | 8545 | TAGAAACAGCGACAACAATAgttttAGAGCTAGAAATAGCAAGTTAAAATAAGGC |
| 25B | psg0492_folP_B | 8695 | GTCAGCACCTTCATCTATCAgttttAGAGCTAGAAATAGCAAGTTAAAATAAGGC |
| 28B | psg0514_cysE_B | 8696 | TCTAATGTTGAACGTGCCGCgttttAGAGCTAGAAATAGCAAGTTAAAATAAGGC |
| 31B | psg0551_folE2_B | 8697 | GTCATTTCGTTCTTAGTAGTgttttAGAGCTAGAAATAGCAAGTTAAAATAAGGC |
| 32B | psg0570_eutD_B | 8698 | ACACGTTCGTCCTCTCCTTCgttttAGAGCTAGAAATAGCAAGTTAAAATAAGGC |
| 33B | psg0572_mvaK1_B | 9277 | CGAATAGTTCCCGCTCTCTAgttttAGAGCTAGAAATAGCAAGTTAAAATAAGGC |
| 34B | psg0596_argS_B | 8699 | GGAACTTCAATTTTAATATCgttttAGAGCTAGAAATAGCAAGTTAAAATAAGGC |
| 35B | psg0623_tagA_B | 8700 | CGTTGATTGATTTGCAAAAAgttttAGAGCTAGAAATAGCAAGTTAAAATAAGGC |
| 36B | psg0624_tagH_B | 8701 | AAATGTTTTGTTTTTATGTTgttttAGAGCTAGAAATAGCAAGTTAAAATAAGGC |
| 40B | psg0703_ltaS_B | 9278 | CTTTGATTTAACAAGTATTTgttttAGAGCTAGAAATAGCAAGTTAAAATAAGGC |
| 42B | psg0722_murB_B | 9210 | TGATATTAAAGACATGAGAAgttttAGAGCTAGAAATAGCAAGTTAAAATAAGGC |
| 46B | psg0743_hprK_B | 9213 | AGTTTTTCTGTCGTTAACATgttttAGAGCTAGAAATAGCAAGTTAAAATAAGGC |
| 55B | psg0868_spsB_B | 9216 | GATTCACCTTTAATTGTATAgttttAGAGCTAGAAATAGCAAGTTAAAATAAGGC |
| 56B | psg0885_fabH_B | 9217 | TTAATAAAATTTTTAATACCgttttAGAGCTAGAAATAGCAAGTTAAAATAAGGC |
| 62B | psg0922_B | 9220 | AAATAAGGTAAGATCAAACTgttttAGAGCTAGAAATAGCAAGTTAAAATAAGGC |
| 65B | psg0983_ptsH_B | 9221 | AAATTCAATTCGTTTAAAACgttttAGAGCTAGAAATAGCAAGTTAAAATAAGGC |
| 66B | psg0989_rnjA_B | 9286 | ATCCCTAATAAGTTATCATCgttttAGAGCTAGAAATAGCAAGTTAAAATAAGGC |
| 67B | psg0990_rpoY_B | 9287 | GGATAAAAAGTTTGGTAGAAgttttAGAGCTAGAAATAGCAAGTTAAAATAAGGC |
| 68B | psg0991_def _B | 9288 | TTAAAAAATGATTAAAGTGTgttttAGAGCTAGAAATAGCAAGTTAAAATAAGGC |
| 70B | psg1024_coaD_B | 9289 | TAACGTTTAATCATATTAAAgttttAGAGCTAGAAATAGCAAGTTAAAATAAGGC |
| 92B | psg1133_trmD_B | 9291 | TTTACAACATCAGCAATATAgttttAGAGCTAGAAATAGCAAGTTAAAATAAGGC |
| 97B | psg1152_frr_B | 8999 | GCTAATTGTTGTACAGGTGTgttttAGAGCTAGAAATAGCAAGTTAAAATAAGGC |
| 98B | psg1153_uppS_B | 9292 | TCGTAATGACCTTTAATTCTgttttAGAGCTAGAAATAGCAAGTTAAAATAAGGC |
| 100B | psg1157_polC_B | 9293 | AAAATTAAATGCTTTATATTgttttAGAGCTAGAAATAGCAAGTTAAAATAAGGC |
| 103B | psg1176_pgsA_B | 9294 | AACCATCAACAAAATCGCTAgttttAGAGCTAGAAATAGCAAGTTAAAATAAGGC |
| 107B | psg1249_plsY_B | 9295 | TATCATATTCATTTAAATTAgttttAGAGCTAGAAATAGCAAGTTAAAATAAGGC |
| 109B | psg1257_msrR_B | 9296 | TTTCTTAATAAATCGTACTAgttttAGAGCTAGAAATAGCAAGTTAAAATAAGGC |
| 115B | psg1340_recU_B | 9299 | AATACATTTACCGTTAATGTgttttAGAGCTAGAAATAGCAAGTTAAAATAAGGC |
| 116B | psg1341_pbp2_B | 9300 | AATTTAGCTTCGGTAAAAGCgttttAGAGCTAGAAATAGCAAGTTAAAATAAGGC |
| 117B | psg1344_dnaD_B | 9301 | GATTTTAACAATGAACTTTAgttttAGAGCTAGAAATAGCAAGTTAAAATAAGGC |
| 118B | psg1347_birA_B | 9302 | ATACCTTGATACCAAATATCgttttAGAGCTAGAAATAGCAAGTTAAAATAAGGC |
| 119B | psg1348_papS_B | 9303 | CGAACAAATGTAACACCACTgttttAGAGCTAGAAATAGCAAGTTAAAATAAGGC |
| 120B | psg1351_B | 9304 | GTAGTTATAAAATCATAATAgttttAGAGCTAGAAATAGCAAGTTAAAATAAGGC |
| 121B | psg1360_ubiE_B | 9305 | ATACCAGTAACTTCACCTGTgttttAGAGCTAGAAATAGCAAGTTAAAATAAGGC |
| 129B | psg1464_bmfBB_B | 9990 | ACAGGAGAAAATGGCATAGAgttttAGAGCTAGAAATAGCAAGTTAAAATAAGGC |
| 131B | psg1475_accC_B | 9308 | TGCACATATGCTTCTTCGTCgttttAGAGCTAGAAATAGCAAGTTAAAATAAGGC |
| 132B | psg1490_efp_B | 9309 | ACGCTTATGTTAAAACTATAgttttAGAGCTAGAAATAGCAAGTTAAAATAAGGC |
| 133B | psg1492_B | 9552 | AAATGCACAAACGTTTCACTgttttAGAGCTAGAAATAGCAAGTTAAAATAAGGC |
| 134B | psg1520_trmK_B | 9553 | TTCAAACGTTTACTACGACTgttttAGAGCTAGAAATAGCAAGTTAAAATAAGGC |
| 136B | psg1522_dnaG_B | 9067 | ACTTACCAAGTCTAAAATGTgttttAGAGCTAGAAATAGCAAGTTAAAATAAGGC |
| 137B | psg1525_glyS_B | 9068 | CACCGTAAATATCACTACCAgttttAGAGCTAGAAATAGCAAGTTAAAATAAGGC |
| 139B | psg1540_dnaK_B | 9554 | TTTCGCCAAATTTAAGTTATgttttAGAGCTAGAAATAGCAAGTTAAAATAAGGC |
| 140B | psg1541_grpE_B | 9069 | CGTTCTATATTGTCTATTGCgttttAGAGCTAGAAATAGCAAGTTAAAATAAGGC |
| 141B | psg1546_holA_B | 9070 | GTTAATGTTTCTTCAACAATgttttAGAGCTAGAAATAGCAAGTTAAAATAAGGC |
| 143B | psg1567_greA_B | 9071 | TTAATTTTCTCTACAACTTCgttttAGAGCTAGAAATAGCAAGTTAAAATAAGGC |
| 144B | psg1575_alaS_B | 9072 | TTTACAATTCTTGGCTTTTTgttttAGAGCTAGAAATAGCAAGTTAAAATAAGGC |
| 147B | psg1589_dtd_B | 9555 | TGAAACTTTAATTAAATTGCgttttAGAGCTAGAAATAGCAAGTTAAAATAAGGC |
| 148B | psg1593_secF_B | 9073 | ATTTTGTCGCCTTTATTTAAgttttAGAGCTAGAAATAGCAAGTTAAAATAAGGC |
| 151B | psg1611_valS_B | 9323 | TACCAGTTACATTTGGTGGCgttttAGAGCTAGAAATAGCAAGTTAAAATAAGGC |
| 152B | psg1619_hemA_B | 9324 | TACAAAAACTATGAAATATAgttttAGAGCTAGAAATAGCAAGTTAAAATAAGGC |
| 154B | psg1624_B | 9325 | AAATGAGTTGTTTATATGAgttttAGAGCTAGAAATAGCAAGTTAAAATAAGGC |
| 156B | psg1629_thrS_B | 9326 | ATTGATCCATCAGTTTCAAGgttttAGAGCTAGAAATAGCAAGTTAAAATAAGGC |
| 157B | psg1631_dnaB_B | 9327 | ACTGCTTGCGTTCCAATTAGgttttAGAGCTAGAAATAGCAAGTTAAAATAAGGC |
| 158B | psg1632_nrdR_B | 9328 | CTAACTCCGAAGTCAGAGTTgttttAGAGCTAGAAATAGCAAGTTAAAATAAGGC |
| 159B | psg1634_coaE_B | 9329 | CTTCCACACACTTTGCATACgttttAGAGCTAGAAATAGCAAGTTAAAATAAGGC |
| 161B | psg1647_accD_B | 9330 | TGAACTTGACCAGTTAAAAAgttttAGAGCTAGAAATAGCAAGTTAAAATAAGGC |
| 162B | psg1649_dnaE_B | 9331 | ACTGAAACTCCTGACGCATTgttttAGAGCTAGAAATAGCAAGTTAAAATAAGGC |
| 163B | psg1657_ackA_B | 9332 | ACTATTACAGATTATTTTTTgttttAGAGCTAGAAATAGCAAGTTAAAATAAGGC |
| 165B | psg1675_tyrS_B | 9333 | ACCCTTACAACAAATATGTAgttttAGAGCTAGAAATAGCAAGTTAAAATAAGGC |
| 168B | psg1704_leuS_B | 9334 | AAACCAGCACCTGATGGATAgttttAGAGCTAGAAATAGCAAGTTAAAATAAGGC |
| 169B | psg1729_B | 9335 | ACTTTGTCTAATTTTTCCCAgttttAGAGCTAGAAATAGCAAGTTAAAATAAGGC |
| 170B | psg1730_metK_B | 9336 | TGTTGTTGTAGAAATTTCGCgttttAGAGCTAGAAATAGCAAGTTAAAATAAGGC |
| 171B | psg1748_B | 9337 | AGTAGAGTCGCCTATCTCTCgttttAGAGCTAGAAATAGCAAGTTAAAATAAGGC |
| 172B | psg1749_B | 9338 | GTTACAACCCATATGATTGTgttttAGAGCTAGAAATAGCAAGTTAAAATAAGGC |
| 173B | psg1869_map_B | 9339 | AAGATGTTACTTTAGTATTTgttttAGAGCTAGAAATAGCAAGTTAAAATAAGGC |
| 180B | psg1894_pncB_B | 9340 | GTAGACTATAATATAAAGCGgttttAGAGCTAGAAATAGCAAGTTAAAATAAGGC |
| 181B | psg1900_ppaC_B | 9341 | AATGAAGCCACTCCCTCAGCgttttAGAGCTAGAAATAGCAAGTTAAAATAAGGC |
| 182B | psg1911_pmtC_B | 9342 | AAAATATATGAATATAAATCgttttAGAGCTAGAAATAGCAAGTTAAAATAAGGC |
| 185B | psg1988_hld_B | 9345 | AGTATTTATTTCCTACAGTTgttttAGAGCTAGAAATAGCAAGTTAAAATAAGGC |
| 188B | psg2028_acpS_B | 9346 | CTTGACCACCCGCTGTATAAgttttAGAGCTAGAAATAGCAAGTTAAAATAAGGC |
| 191B | psg2055_murA_B | 9347 | ATTTTACTAACATATTCATAgttttAGAGCTAGAAATAGCAAGTTAAAATAAGGC |
| 192B | psg2062_atpF_B | 9348 | CTGAATTTATGCGATAGGCAgttttAGAGCTAGAAATAGCAAGTTAAAATAAGGC |
| 198B | psg2113_dacA_B | 9559 | TGATTATTACTGTCAATCAAgttttAGAGCTAGAAATAGCAAGTTAAAATAAGGC |
| 200B | psg2184_secY_B | 9560 | CCAGATTCTACTAATAAAGCgttttAGAGCTAGAAATAGCAAGTTAAAATAAGGC |
| 202B | psg2238_ureA_B | 9349 | ATCTAATCGAAAACAAATAGgttttAGAGCTAGAAATAGCAAGTTAAAATAAGGC |
| 203B | psg2283_rpiA_B | 9350 | ATTTTAAATAATACTCGTTAgttttAGAGCTAGAAATAGCAAGTTAAAATAAGGC |
| 208B | psg005_gyrB_B | 9608 | GTCGATCCTATATACATACCgttttAGAGCTAGAAATAGCAAGTTAAAATAAGGC |
| 209B | psg0527_rpoB_B | 9609 | ATAAAAAGACAAAAAGAAAAgttttAGAGCTAGAAATAGCAAGTTAAAATAAGGC |
| 211B | psg0639_B | 9611 | ATATATCTCATTGGCATAACgttttAGAGCTAGAAATAGCAAGTTAAAATAAGGC |
| 212B | psg1359_B | 9612 | TACTCAGAATAACAAATGCTgttttAGAGCTAGAAATAGCAAGTTAAAATAAGGC |
| 213B | psg1590_relA_B | 9613 | TGTATGGTAATCCGTTTTTTgttttAGAGCTAGAAATAGCAAGTTAAAATAAGGC |
| 214B | psg1791_cbf1_B | 9614 | TTAACATGTACAATTTCTTCgttttAGAGCTAGAAATAGCAAGTTAAAATAAGGC |
| 219B | psg0015_rplL_B | 9615 | TTTAAGTTGTGTTGCCGCATgttttAGAGCTAGAAATAGCAAGTTAAAATAAGGC |
| 221B | psg0479_rplY_B | 9675 | ACGAAACTATTATACACGTTgttttAGAGCTAGAAATAGCAAGTTAAAATAAGGC |
| 222B | psg0522_rplK_B | 9676 | ACGAAACTATTATACACGTTgttttAGAGCTAGAAATAGCAAGTTAAAATAAGGC |
| 223B | psg0524_rplJ_B | 9616 | CTTCAGCTACTGTTAATCCAgttttAGAGCTAGAAATAGCAAGTTAAAATAAGGC |
| 225B | psg0990_rpoY_B | 9287 | TAACGATTTGGTTAATAACTgttttAGAGCTAGAAATAGCAAGTTAAAATAAGGC |
| 231B | psg1166_rpsO_B | 9677 | AGTACAGCGATTTGTACTTCgttttAGAGCTAGAAATAGCAAGTTAAAATAAGGC |
| 234B | psg1545_rpsT_B | 9620 | CTTTTAGGAGGTGACAGAAAgttttAGAGCTAGAAATAGCAAGTTAAAATAAGGC |
| 235B | psg1601_rpmA_B | 9621 | TTGTCGTCATAATTGATATCgttttAGAGCTAGAAATAGCAAGTTAAAATAAGGC |
| 236B | psg1625_rplT_B | 9622 | TAACACGTTTAGCTGCTCCGgttttAGAGCTAGAAATAGCAAGTTAAAATAAGGC |
| 237B | psg1666_rpsD_B | 9623 | GTTACGTGACACGTCCGCATgttttAGAGCTAGAAATAGCAAGTTAAAATAAGGC |
| 239B | psg2171_rpsI_B | 9624 | ACTGTGATGTTACCTTCACCgttttAGAGCTAGAAATAGCAAGTTAAAATAAGGC |
| 240B | psg2177_rplQ_B | 9625 | GTTGAGAAATTAATCACTTTgttttAGAGCTAGAAATAGCAAGTTAAAATAAGGC |
| 241B | psg0386_xpt_B | 10049 | TGCGATACCGGAAGCTTCAAgttttAGAGCTAGAAATAGCAAGTTAAAATAAGGC |
| 245B | psg1072_mraZ_B | 10057 | ATTTAAGTCATAACGAAACTgttttAGAGCTAGAAATAGCAAGTTAAAATAAGGC |
| 249B | psg1158_rimP_B | 10065 | TAAGACATTGAAAAGAAATAgttttAGAGCTAGAAATAGCAAGTTAAAATAAGGC |
| 254B | psg2029_B | 10075 | CGTATTTTAAGTTAATCGATgttttAGAGCTAGAAATAGCAAGTTAAAATAAGGC |
| 256B | psg0014_gdpP_B | 10079 | AGTCCTACAATATGTGTCGTgttttAGAGCTAGAAATAGCAAGTTAAAATAAGGC |

# **Supplementary Table 3. Lisbon CRISPRi Mutant Library strain list**

This table lists all strains for the Lisbon CRISPRi Mutant Library (LCML) constructed in this study. For all 261 targeted essential genes, an initial sgRNA was designed (A clones). When these clones did not did not exhibit a reduction in R greater than 0.5, a second sgRNA was designed and tested, and these strains were given the suffix B.

| **Name** | **Description** |
| --- | --- |
| LCML1 | JE2 *Δspa:P_xyl/tetO3_-dcas9_Spy_* containing psg0001 (*dnaA*) |
| LCML2 | JE2 *Δspa:P_xyl/tetO3_-dcas9_Spy_* containingsg0002 (*dnaN*) |
| LCML3 | JE2 *Δspa:P_xyl/tetO3_-dcas9_Spy_* containing psg0003 |
| LCML4 | JE2 *Δspa:P_xyl/tetO3_-dcas9_Spy_* containing psg0009 (*serS*) |
| LCML5 | JE2 *Δspa:P_xyl/tetO3_-dcas9_Spy_* containing psg0016 (*dnaB*) |
| LCML6 | JE2 *Δspa:P_xyl/tetO3_-dcas9_Spy_* containing psg0020 (*walR*) |
| LCML7 | JE2 *Δspa:P_xyl/tetO3_-dcas9_Spy_* containing psg0024 (*walJ*) |
| LCML8 | JE2 *Δspa:P_xyl/tetO3_-dcas9_Spy_* containing psg0089 |
| LCML9 | JE2 *Δspa:P_xyl/tetO3_-dcas9_Spy_* containing psg0248 (*tarF*) |
| LCML10 | JE2 *Δspa:P_xyl/tetO3_-dcas9_Spy_* containing psg0249 (*ispD*) |
| LCML11 | JE2 *Δspa:P_xyl/tetO3_-dcas9_Spy_* containing psg0363 |
| LCML12 | JE2 *Δspa:P_xyl/tetO3_-dcas9_Spy_* containing psg0367 (*ssb*) |
| LCML13 | JE2 *Δspa:P_xyl/tetO3_-dcas9_Spy_* containing psg0388 (*guaB*) |
| LCML14 | JE2 *Δspa:P_xyl/tetO3_-dcas9_Spy_* containing psg0452 (*dnaX*) |
| LCML15 | JE2 *Δspa:P_xyl/tetO3_-dcas9_Spy_* containing psg0453 |
| LCML16 | JE2 *Δspa:P_xyl/tetO3_-dcas9_Spy_* containing psg00459 (*tmk*) |
| LCML17 | JE2 *Δspa:P_xyl/tetO3_-dcas9_Spy_* containing psg0461 (*holB*) |
| LCML18 | JE2 *Δspa:P_xyl/tetO3_-dcas9_Spy_* containing psg0467 (*metS*) |
| LCML19 | JE2 *Δspa:P_xyl/tetO3_-dcas9_Spy_* containing psg0477 (*glmU*) |
| LCML20 | JE2 *Δspa:P_xyl/tetO3_-dcas9_Spy_* containing psg0478 (*prs*) |
| LCML21 | JE2 *Δspa:P_xyl/tetO3_-dcas9_Spy_* containing psg0480 (*pth*) |
| LCML22 | JE2 *Δspa:P_xyl/tetO3_-dcas9_Spy_* containing psg0484 |
| LCML23 | JE2 *Δspa:P_xyl/tetO3_-dcas9_Spy_* containing psg0485 (*divIC*) |
| LCML24 | JE2 *Δspa:P_xyl/tetO3_-dcas9_Spy_* containing psg0487 (*tilS*) |
| LCML25 | JE2 *Δspa:P_xyl/tetO3_-dcas9_Spy_* containing psg0492 (*folP*) |
| LCML26 | JE2 *Δspa:P_xyl/tetO3_-dcas9_Spy_* containing psg0496 (*lysS*) |
| LCML27 | JE2 *Δspa:P_xyl/tetO3_-dcas9_Spy_* containing psg0513 (*gltX*) |
| LCML28 | JE2 *Δspa:P_xyl/tetO3_-dcas9_Spy_* containing psg0514 (*cysE*) |
| LCML29 | JE2 *Δspa:P_xyl/tetO3_-dcas9_Spy_* containing psg0532 (*fusA*) |
| LCML30 | JE2 *Δspa:P_xyl/tetO3_-dcas9_Spy_* containing psg0533 (*tuf*) |
| LCML31 | JE2 *Δspa:P_xyl/tetO3_-dcas9_Spy_* containing psg0551 (*folE2*) |
| LCML32 | JE2 *Δspa:P_xyl/tetO3_-dcas9_Spy_* containing psg0570 (*eutD*) |
| LCML33 | JE2 *Δspa:P_xyl/tetO3_-dcas9_Spy_* containing psg0572 (*mvaK1*) |
| LCML34 | JE2 *Δspa:P_xyl/tetO3_-dcas9_Spy_* containing psg0596 (*argS*) |
| LCML35 | JE2 *Δspa:P_xyl/tetO3_-dcas9_Spy_* containing psg0623 (*tagA*) |
| LCML36 | JE2 *Δspa:P_xyl/tetO3_-dcas9_Spy_* containing psg0624 (*tagH*) |
| LCML37 | JE2 *Δspa:P_xyl/tetO3_-dcas9_Spy_* containing psg0625 (*tagG*) |
| LCML38 | JE2 *Δspa:P_xyl/tetO3_-dcas9_Spy_* containing psg0626 (*tagB*) |
| LCML39 | JE2 *Δspa:P_xyl/tetO3_-dcas9_Spy_* containing psg0628 (*tagD*) |
| LCML40 | JE2 *Δspa:P_xyl/tetO3_-dcas9_Spy_* containing psg0703 (*ltaS*) |
| LCML41 | JE2 *Δspa:P_xyl/tetO3_-dcas9_Spy_* containing psg0715 (*nrdl*) |
| LCML42 | JE2 *Δspa:P_xyl/tetO3_-dcas9_Spy_* containing psg0722 (*murB*) |
| LCML43 | JE2 *Δspa:P_xyl/tetO3_-dcas9_Spy_* containing psg0731 (*tagO*) |
| LCML44 | JE2 *Δspa:P_xyl/tetO3_-dcas9_Spy_* containing psg0737 (*secA*) |
| LCML45 | JE2 *Δspa:P_xyl/tetO3_-dcas9_Spy_* containing psg0738 (*prfB*) |
| LCML46 | JE2 *Δspa:P_xyl/tetO3_-dcas9_Spy_* containing psg0743 (*hprK*) |
| LCML47 | JE2 *Δspa:P_xyl/tetO3_-dcas9_Spy_* containing psg0749 |
| LCML48 | JE2 *Δspa:P_xyl/tetO3_-dcas9_Spy_* containing psg0756 (*gapA*) |
| LCML49 | JE2 *Δspa:P_xyl/tetO3_-dcas9_Spy_* containing psg0761 |
| LCML50 | JE2 *Δspa:P_xyl/tetO3_-dcas9_Spy_* containing psg0765 (*smpB*) |
| LCML51 | JE2 *Δspa:P_xyl/tetO3_-dcas9_Spy_* containing psg0818 (*sufC*) |
| LCML52 | JE2 *Δspa:P_xyl/tetO3_-dcas9_Spy_* containing psg0835 (*dltA*) |
| LCML53 | JE2 *Δspa:P_xyl/tetO3_-dcas9_Spy_* containing psg0858 |
| LCML54 | JE2 *Δspa:P_xyl/tetO3_-dcas9_Spy_* containing psg0865 (*pgi*) |
| LCML55 | JE2 *Δspa:P_xyl/tetO3_-dcas9_Spy_* containing psg0868 (*spsB*) |
| LCML56 | JE2 *Δspa:P_xyl/tetO3_-dcas9_Spy_* containing psg0885 (*fabH*) |
| LCML57 | JE2 *Δspa:P_xyl/tetO3_-dcas9_Spy_* containing psg0897 (*trpS*) |
| LCML58 | JE2 *Δspa:P_xyl/tetO3_-dcas9_Spy_* containing psg0898 (*spxA*) |
| LCML59 | JE2 *Δspa:P_xyl/tetO3_-dcas9_Spy_* containing psg0908 (*ppnK*) |
| LCML60 | JE2 *Δspa:P_xyl/tetO3_-dcas9_Spy_* containing psg0912 (*fabI*) |
| LCML61 | JE2 *Δspa:P_xyl/tetO3_-dcas9_Spy_* containing psg0919 (*murE*) |
| LCML62 | JE2 *Δspa:P_xyl/tetO3_-dcas9_Spy_* containing psg0922 |
| LCML63 | JE2 *Δspa:P_xyl/tetO3_-dcas9_Spy_* containing psg0944 (*menA*) |
| LCML64 | JE2 *Δspa:P_xyl/tetO3_-dcas9_Spy_* containing psg0948 (*menB*) |
| LCML65 | JE2 *Δspa:P_xyl/tetO3_-dcas9_Spy_* containing psg0983 (*ptsH*) |
| LCML66 | JE2 *Δspa:P_xyl/tetO3_-dcas9_Spy_* containing psg0989 (*rnjA*) |
| LCML67 | JE2 *Δspa:P_xyl/tetO3_-dcas9_Spy_* containing psg0990 (*rpoY*) |
| LCML68 | JE2 *Δspa:P_xyl/tetO3_-dcas9_Spy_* containing psg0991 (*def*) |
| LCML69 | JE2 *Δspa:P_xyl/tetO3_-dcas9_Spy_* containing psg1013 (*ftsW*) |
| LCML70 | JE2 *Δspa:P_xyl/tetO3_-dcas9_Spy_* containing psg1024 (*coaD*) |
| LCML71 | JE2 *Δspa:P_xyl/tetO3_-dcas9_Spy_* containing psg1026 |
| LCML72 | JE2 *Δspa:P_xyl/tetO3_-dcas9_Spy_* containing psg1037 (*pheS*) |
| LCML73 | JE2 *Δspa:P_xyl/tetO3_-dcas9_Spy_* containing psg1044 (*trxA*) |
| LCML74 | JE2 *Δspa:P_xyl/tetO3_-dcas9_Spy_* containing psg1049 (*murI*) |
| LCML75 | JE2 *Δspa:P_xyl/tetO3_-dcas9_Spy_* containing psg1074 (*ftsL*) |
| LCML76 | JE2 *Δspa:P_xyl/tetO3_-dcas9_Spy_* containing psg1075 (*pbpA*) |
| LCML77 | JE2 *Δspa:P_xyl/tetO3_-dcas9_Spy_* containing psg1076 (*mraY*) |
| LCML78 | JE2 *Δspa:P_xyl/tetO3_-dcas9_Spy_* containing psg1077 (*murD*) |
| LCML79 | JE2 *Δspa:P_xyl/tetO3_-dcas9_Spy_* containing psg1078 (*divIB*) |
| LCML80 | JE2 *Δspa:P_xyl/tetO3_-dcas9_Spy_* containing psg1079 (*ftsA*) |
| LCML81 | JE2 *Δspa:P_xyl/tetO3_-dcas9_Spy_* containing psg1082 |
| LCML82 | JE2 *Δspa:P_xyl/tetO3_-dcas9_Spy_* containing psg1083 (*sepF*) |
| LCML83 | JE2 *Δspa:P_xyl/tetO3_-dcas9_Spy_* containing psg1087 (*ileS*) |
| LCML84 | JE2 *Δspa:P_xyl/tetO3_-dcas9_Spy_* containing psg1102 (*gmk*) |
| LCML85 | JE2 *Δspa:P_xyl/tetO3_-dcas9_Spy_* containing psg1104 (*coaBC*) |
| LCML86 | JE2 *Δspa:P_xyl/tetO3_-dcas9_Spy_* containing psg1105 (*priA*) |
| LCML87 | JE2 *Δspa:P_xyl/tetO3_-dcas9_Spy_* containing psg1109 (*fmt*) |
| LCML88 | JE2 *Δspa:P_xyl/tetO3_-dcas9_Spy_* containing psg1115 (*rpe*) |
| LCML89 | JE2 *Δspa:P_xyl/tetO3_-dcas9_Spy_* containing psg1122 (*plsX*) |
| LCML90 | JE2 *Δspa:P_xyl/tetO3_-dcas9_Spy_* containing psg1125 (*acpP*) |
| LCML91 | JE2 *Δspa:P_xyl/tetO3_-dcas9_Spy_* containing psg1128 (*ftsY*) |
| LCML92 | JE2 *Δspa:P_xyl/tetO3_-dcas9_Spy_* containing psg1133 (*trmD*) |
| LCML93 | JE2 *Δspa:P_xyl/tetO3_-dcas9_Spy_* containing psg1136 (*rbgA*) |
| LCML94 | JE2 *Δspa:P_xyl/tetO3_-dcas9_Spy_* containing psg1143 (*topA*) |
| LCML95 | JE2 *Δspa:P_xyl/tetO3_-dcas9_Spy_* containing psg1150 (*tsf*) |
| LCML96 | JE2 *Δspa:P_xyl/tetO3_-dcas9_Spy_* containing psg1151 (*pyrH*) |
| LCML97 | JE2 *Δspa:P_xyl/tetO3_-dcas9_Spy_* containing psg1152 (*frr*) |
| LCML98 | JE2 *Δspa:P_xyl/tetO3_-dcas9_Spy_* containing psg1153 (*uppS*) |
| LCML99 | JE2 *Δspa:P_xyl/tetO3_-dcas9_Spy_* containing psg1156 (*proS*) |
| LCML100 | JE2 *Δspa:P_xyl/tetO3_-dcas9_Spy_* containing psg1157 (*polC*) |
| LCML101 | JE2 *Δspa:P_xyl/tetO3_-dcas9_Spy_* containing psg1159 (*nusA*) |
| LCML102 | JE2 *Δspa:P_xyl/tetO3_-dcas9_Spy_* containing psg1168 (*rnjB*) |
| LCML103 | JE2 *Δspa:P_xyl/tetO3_-dcas9_Spy_* containing psg1176 (*pgsA*) |
| LCML104 | JE2 *Δspa:P_xyl/tetO3_-dcas9_Spy_* containing psg1200 (*glnR*) |
| LCML105 | JE2 *Δspa:P_xyl/tetO3_-dcas9_Spy_* containing psg1237 (*lexA*) |
| LCML106 | JE2 *Δspa:P_xyl/tetO3_-dcas9_Spy_* containing psg1239 (*tkt*) |
| LCML107 | JE2 *Δspa:P_xyl/tetO3_-dcas9_Spy_* containing psg1249 (*plsY*) |
| LCML108 | JE2 *Δspa:P_xyl/tetO3_-dcas9_Spy_* containing psg1250 (*parE*) |
| LCML109 | JE2 *Δspa:P_xyl/tetO3_-dcas9_Spy_* containing psg1257 (*msrR*) |
| LCML110 | JE2 *Δspa:P_xyl/tetO3_-dcas9_Spy_* containing psg1269 (*femA*) |
| LCML111 | JE2 *Δspa:P_xyl/tetO3_-dcas9_Spy_* containing psg1270 (*femB*) |
| LCML112 | JE2 *Δspa:P_xyl/tetO3_-dcas9_Spy_* containing psg1311 (*murG*) |
| LCML113 | JE2 *Δspa:P_xyl/tetO3_-dcas9_Spy_* containing psg1319 (*folA*) |
| LCML114 | JE2 *Δspa:P_xyl/tetO3_-dcas9_Spy_* containing psg1320 (*thyA*) |
| LCML115 | JE2 *Δspa:P_xyl/tetO3_-dcas9_Spy_* containing psg1340 (*recU*) |
| LCML116 | JE2 *Δspa:P_xyl/tetO3_-dcas9_Spy_* containing psg1341 (*pbp2*) |
| LCML117 | JE2 *Δspa:P_xyl/tetO3_-dcas9_Spy_* containing psg1344 (*dnaD*) |
| LCML118 | JE2 *Δspa:P_xyl/tetO3_-dcas9_Spy_* containing psg1347 (*birA*) |
| LCML119 | JE2 *Δspa:P_xyl/tetO3_-dcas9_Spy_* containing psg1348 (*papS*) |
| LCML120 | JE2 *Δspa:P_xyl/tetO3_-dcas9_Spy_* containing psg1351 |
| LCML121 | JE2 *Δspa:P_xyl/tetO3_-dcas9_Spy_* containing psg1360 (*ubiE*) |
| LCML122 | JE2 *Δspa:P_xyl/tetO3_-dcas9_Spy_* containing psg1362 (*hup*) |
| LCML123 | JE2 *Δspa:P_xyl/tetO3_-dcas9_Spy_* containing psg1364 (*engA*) |
| LCML124 | JE2 *Δspa:P_xyl/tetO3_-dcas9_Spy_* containing psg1367 (*cmk*) |
| LCML125 | JE2 *Δspa:P_xyl/tetO3_-dcas9_Spy_* containing psg1373 (*fer*) |
| LCML126 | JE2 *Δspa:P_xyl/tetO3_-dcas9_Spy_* containing psg1453 (*rnz*) |
| LCML127 | JE2 *Δspa:P_xyl/tetO3_-dcas9_Spy_* containing psg1454 (*zwf*) |
| LCML128 | JE2 *Δspa:P_xyl/tetO3_-dcas9_Spy_* containing psg1459 (*gnd*) |
| LCML129 | JE2 *Δspa:P_xyl/tetO3_-dcas9_Spy_* containing psg1464 (*bmfBB*) |
| LCML130 | JE2 *Δspa:P_xyl/tetO3_-dcas9_Spy_* containing psg1466 (*bmfBAA*) |
| LCML131 | JE2 *Δspa:P_xyl/tetO3_-dcas9_Spy_* containing psg1475 (*accC*) |
| LCML132 | JE2 *Δspa:P_xyl/tetO3_-dcas9_Spy_* containing psg1490 (*efp*) |
| LCML133 | JE2 *Δspa:P_xyl/tetO3_-dcas9_Spy_* containing psg1492 |
| LCML134 | JE2 *Δspa:P_xyl/tetO3_-dcas9_Spy_* containing psg1520 (*trmK*) |
| LCML135 | JE2 *Δspa:P_xyl/tetO3_-dcas9_Spy_* containing psg1521(*rpoD*) |
| LCML136 | JE2 *Δspa:P_xyl/tetO3_-dcas9_Spy_* containing psg1522 (*dnaG*) |
| LCML137 | JE2 *Δspa:P_xyl/tetO3_-dcas9_Spy_* containing psg1525 (*glyS*) |
| LCML138 | JE2 *Δspa:P_xyl/tetO3_-dcas9_Spy_* containing psg1530 (*ybeY*) |
| LCML139 | JE2 *Δspa:P_xyl/tetO3_-dcas9_Spy_* containing psg1540 (*dnaK*) |
| LCML140 | JE2 *Δspa:P_xyl/tetO3_-dcas9_Spy_* containing psg1541 (*grpE*) |
| LCML141 | JE2 *Δspa:P_xyl/tetO3_-dcas9_Spy_* containing psg1546 (*holA*) |
| LCML142 | JE2 *Δspa:P_xyl/tetO3_-dcas9_Spy_* containing psg1557 |
| LCML143 | JE2 *Δspa:P_xyl/tetO3_-dcas9_Spy_* containing psg1567 (*greA*) |
| LCML144 | JE2 *Δspa:P_xyl/tetO3_-dcas9_Spy_* containing psg1575 (*alaS*) |
| LCML[145](javascript:void(0)) | JE2 *Δspa:P_xyl/tetO3_-dcas9_Spy_* containing p[sg1579 (*iscS*)](javascript:void(0)) |
| LCML[146](javascript:void(0)) | JE2 *Δspa:P_xyl/tetO3_-dcas9_Spy_* containing p[sg1587 (*hisS*)](javascript:void(0)) |
| LCML147 | JE2 *Δspa:P_xyl/tetO3_-dcas9_Spy_* containing psg1589 (*dtd*) |
| LCML148 | JE2 *Δspa:P_xyl/tetO3_-dcas9_Spy_* containing psg1593 (*secF*) |
| LCML[149](javascript:void(0)) | JE2 *Δspa:P_xyl/tetO3_-dcas9_Spy_* containing p[sg1600 (*obgE*)](javascript:void(0)) |
| LCML[150](javascript:void(0)) | JE2 *Δspa:P_xyl/tetO3_-dcas9_Spy_* containing psg1602 |
| LCML151 | JE2 *Δspa:P_xyl/tetO3_-dcas9_Spy_* containing psg1611 (*valS*) |
| LCML152 | JE2 *Δspa:P_xyl/tetO3_-dcas9_Spy_* containing psg1619 (*hemA*) |
| LCML153 | JE2 *Δspa:P_xyl/tetO3_-dcas9_Spy_* containing psg1620 (*engB*) |
| LCML154 | JE2 *Δspa:P_xyl/tetO3_-dcas9_Spy_* containing psg1624 |
| LCML155 | JE2 *Δspa:P_xyl/tetO3_-dcas9_Spy_* containing psg1627 (*infC*) |
| LCML156 | JE2 *Δspa:P_xyl/tetO3_-dcas9_Spy_* containing psg1629 (*thrS*) |
| LCML157 | JE2 *Δspa:P_xyl/tetO3_-dcas9_Spy_* containing psg1631 (*dnaB*) |
| LCML158 | JE2 *Δspa:P_xyl/tetO3_-dcas9_Spy_* containing psg1632 (*nrdR*) |
| LCML159 | JE2 *Δspa:P_xyl/tetO3_-dcas9_Spy_* containing psg1634 (*coaE*) |
| LCML160 | JE2 *Δspa:P_xyl/tetO3_-dcas9_Spy_* containing psg1645 (*pfkA*) |
| LCML161 | JE2 *Δspa:P_xyl/tetO3_-dcas9_Spy_* containing psg1647 (*accD*) |
| LCML162 | JE2 *Δspa:P_xyl/tetO3_-dcas9_Spy_* containing psg1649 (*dnaE*) |
| LCML163 | JE2 *Δspa:P_xyl/tetO3_-dcas9_Spy_* containing psg1657 (*ackA*) |
| LCML164 | JE2 *Δspa:P_xyl/tetO3_-dcas9_Spy_* containing psg1673 (*plsC*) |
| LCML165 | JE2 *Δspa:P_xyl/tetO3_-dcas9_Spy_* containing psg1675 (*tyrS*) |
| LCML166 | JE2 *Δspa:P_xyl/tetO3_-dcas9_Spy_* containing psg1686 (*murC*) |
| LCML167 | JE2 *Δspa:P_xyl/tetO3_-dcas9_Spy_* containing psg1688 |
| LCML168 | JE2 *Δspa:P_xyl/tetO3_-dcas9_Spy_* containing psg1704 (*leuS*) |
| LCML169 | JE2 *Δspa:P_xyl/tetO3_-dcas9_Spy_* containing psg1729 |
| LCML170 | JE2 *Δspa:P_xyl/tetO3_-dcas9_Spy_* containing psg1730 (*metK*) |
| LCML171 | JE2 *Δspa:P_xyl/tetO3_-dcas9_Spy_* containing psg1748 |
| LCML172 | JE2 *Δspa:P_xyl/tetO3_-dcas9_Spy_* containing psg1749 |
| LCML173 | JE2 *Δspa:P_xyl/tetO3_-dcas9_Spy_* containing psg1869 (*map*) |
| LCML174 | JE2 *Δspa:P_xyl/tetO3_-dcas9_Spy_* containing psg1873 (*murT*) |
| LCML175 | JE2 *Δspa:P_xyl/tetO3_-dcas9_Spy_* containing psg1879 (*dgkB*) |
| LCML176 | JE2 *Δspa:P_xyl/tetO3_-dcas9_Spy_* containing psg1881 (*gatA*) |
| LCML177 | JE2 *Δspa:P_xyl/tetO3_-dcas9_Spy_* containing psg1882 (*gatC*) |
| LCML178 | JE2 *Δspa:P_xyl/tetO3_-dcas9_Spy_* containing psg1885 (*ligA*) |
| LCML179 | JE2 *Δspa:P_xyl/tetO3_-dcas9_Spy_* containing psg1886 (*pcrA*) |
| LCML180 | JE2 *Δspa:P_xyl/tetO3_-dcas9_Spy_* containing psg1894 (*pncB*) |
| LCML181 | JE2 *Δspa:P_xyl/tetO3_-dcas9_Spy_* containing psg1900 (*ppaC*) |
| LCML182 | JE2 *Δspa:P_xyl/tetO3_-dcas9_Spy_* containing psg1911 (*pmtC*) |
| LCML183 | JE2 *Δspa:P_xyl/tetO3_-dcas9_Spy_* containing psg1913 (*pmtA*) |
| LCML184 | JE2 *Δspa:P_xyl/tetO3_-dcas9_Spy_* containing psg1983 (*groES*) |
| LCML185 | JE2 *Δspa:P_xyl/tetO3_-dcas9_Spy_* containing psg1988 (*hld*) |
| LCML186 | JE2 *Δspa:P_xyl/tetO3_-dcas9_Spy_* containing psg2002 (*gcp*) |
| LCML187 | JE2 *Δspa:P_xyl/tetO3_-dcas9_Spy_* containing psg2005 (*tsaE*) |
| LCML188 | JE2 *Δspa:P_xyl/tetO3_-dcas9_Spy_* containing psg2028 (*acpS*) |
| LCML189 | JE2 *Δspa:P_xyl/tetO3_-dcas9_Spy_* containing psg2039 (*ddl*) |
| LCML190 | JE2 *Δspa:P_xyl/tetO3_-dcas9_Spy_* containing psg2046 (*oxaA*) |
| LCML191 | JE2 *Δspa:P_xyl/tetO3_-dcas9_Spy_* containing psg2055 (*murA*) |
| LCML192 | JE2 *Δspa:P_xyl/tetO3_-dcas9_Spy_* containing psg2062 (*atpF*) |
| LCML193 | JE2 *Δspa:P_xyl/tetO3_-dcas9_Spy_* containing psg2072 (*prfA*) |
| LCML194 | JE2 *Δspa:P_xyl/tetO3_-dcas9_Spy_* containing psg2079 (*fbaA*) |
| LCML195 | JE2 *Δspa:P_xyl/tetO3_-dcas9_Spy_* containing psg2081 (*pyrG*) |
| LCML196 | JE2 *Δspa:P_xyl/tetO3_-dcas9_Spy_* containing psg2084 (*coaA*) |
| LCML197 | JE2 *Δspa:P_xyl/tetO3_-dcas9_Spy_* containing psg2104 (*glmS*) |
| LCML198 | JE2 *Δspa:P_xyl/tetO3_-dcas9_Spy_* containing psg2113 (*dacA*) |
| LCML199 | JE2 *Δspa:P_xyl/tetO3_-dcas9_Spy_* containing psg2182 (*infA*) |
| LCML200 | JE2 *Δspa:P_xyl/tetO3_-dcas9_Spy_* containing psg2184 (*secY*) |
| LCML201 | JE2 *Δspa:P_xyl/tetO3_-dcas9_Spy_* containing psg2214 (*femX*) |
| LCML202 | JE2 *Δspa:P_xyl/tetO3_-dcas9_Spy_* containing psg2238 (*ureA*) |
| LCML203 | JE2 *Δspa:P_xyl/tetO3_-dcas9_Spy_* containing psg2283 (*rpiA*) |
| LCML204 | JE2 *Δspa:P_xyl/tetO3_-dcas9_Spy_* containing psg2292 (*fni*) |
| LCML205 | JE2 *Δspa:P_xyl/tetO3_-dcas9_Spy_* containing psg2483 (*mvaA*) |
| LCML206 | JE2 *Δspa:P_xyl/tetO3_-dcas9_Spy_* containing psg2646 (*trmE*) |
| LCML207 | JE2 *Δspa:P_xyl/tetO3_-dcas9_Spy_* containing psg2484 (*mvaS*) |
| LCML208 | JE2 *Δspa:P_xyl/tetO3_-dcas9_Spy_* containing psg0005 (*gyrB*) |
| LCML209 | JE2 *Δspa:P_xyl/tetO33_-dcas9_Spy_* containing psg0527 (*rpoB*) |
| LCML210 | JE2 *Δspa:P_xyl/tetO3_-dcas9_Spy_* containing psg0425 (*mpsA*) |
| LCML211 | JE2 *Δspa:P_xyl/tetO3_-dcas9_Spy_* containing psg0639 |
| LCML212 | JE2 *Δspa:P_xyl/tetO3_-dcas9_Spy_* containing psg1359 |
| LCML213 | JE2 *Δspa:P_xyl/tetO3_-dcas9_Spy_* containing psg1590 (*relA*) |
| LCML214 | JE2 *Δspa:P_xyl/tetO3_-dcas9_Spy_* containing psg1791 (*cbf1*) |
| LCML215 | JE2 *Δspa:P_xyl/tetO3_-dcas9_Spy_* containing psg2183 (*adk*) |
| LCML216 | JE2 *Δspa:P_xyl/tetO3_-dcas9_Spy_* containing psg2185 (*rplO*) |
| LCML217 | JE2 *Δspa:P_xyl/tetO3_-dcas9_Spy_* containing psg2647 (*rnpA*) |
| LCML218 | JE2 *Δspa:P_xyl/tetO3_-dcas9_Spy_* containing psg2648 (*rpmH*) |
| LCML219 | JE2 *Δspa:P_xyl/tetO3_-dcas9_Spy_* containing psg0015 (*rplI*) |
| LCML220 | JE2 *Δspa:P_xyl/tetO3_-dcas9_Spy_* containing psg0366 (*rpsF*) |
| LCML221 | JE2 *Δspa:P_xyl/tetO3_-dcas9_Spy_* containing psg0479 (*rplY*) |
| LCML222 | JE2 *Δspa:P_xyl/tetO3_-dcas9_Spy_* containing psg0522 (*rplK*) |
| LCML223 | JE2 *Δspa:P_xyl/tetO3_-dcas9_Spy_* containing psg0524 (*rplJ*) |
| LCML224 | JE2 *Δspa:P_xyl/tetO3_-dcas9_Spy_* containing psg0530 (*rpsL*) |
| LCML225 | JE2 *Δspa:P_xyl/tetO3_-dcas9_Spy_* containing psg0990 (*rpoY*) |
| LCML226 | JE2 *Δspa:P_xyl/tetO3_-dcas9_Spy_* containing psg1027 (*rpmF*) |
| LCML227 | JE2 *Δspa:P_xyl/tetO3_-dcas9_Spy_* containing psg1117 (*rpmB*) |
| LCML228 | JE2 *Δspa:P_xyl/tetO3_-dcas9_Spy_* containing psg1131 (*rpsP*) |
| LCML229 | JE2 *Δspa:P_xyl/tetO3_-dcas9_Spy_* containing psg1134 (*rplS*) |
| LCML230 | JE2 *Δspa:P_xyl/tetO3_-dcas9_Spy_* containing psg1149 (*rpsB*) |
| LCML231 | JE2 *Δspa:P_xyl/tetO3_-dcas9_Spy_* containing psg1166 (*rpsO*) |
| LCML232 | JE2 *Δspa:P_xyl/tetO3_-dcas9_Spy_* containing psg1511 (*rpmG*) |
| LCML233 | JE2 *Δspa:P_xyl/tetO3_-dcas9_Spy_* containing psg1535 (*rpsU*) |
| LCML234 | JE2 *Δspa:P_xyl/tetO3_-dcas9_Spy_* containing psg1545 (*rpsT*) |
| LCML235 | JE2 *Δspa:P_xyl/tetO3_-dcas9_Spy_* containing psg1601 (*rpmA*) |
| LCML236 | JE2 *Δspa:P_xyl/tetO3_-dcas9_Spy_* containing psg1625 (*rplT*) |
| LCML237 | JE2 *Δspa:P_xyl/tetO3_-dcas9_Spy_* containing psg1666 (*rpsD*) |
| LCML238 | JE2 *Δspa:P_xyl/tetO3_-dcas9_Spy_* containing psg2074 (*rpmE2*) |
| LCML239 | JE2 *Δspa:P_xyl/tetO3_-dcas9_Spy_* containing psg2171 (*rpsI*) |
| LCML240 | JE2 *Δspa:P_xyl/tetO3_-dcas9_Spy_* containing psg2177 (*rplQ*) |
| LCML241 | JE2 *Δspa:P_xyl/tetO3_-dcas9_Spy_* containing psg0386 (*xpt*) |
| LCML242 | JE2 *Δspa:P_xyl/tetO3_-dcas9_Spy_* containing psg0529 (*rplGB*) |
| LCML243 | JE2 *Δspa:P_xyl/tetO3_-dcas9_Spy_* containing psg0755 (*gapR*) |
| LCML244 | JE2 *Δspa:P_xyl/tetO3_-dcas9_Spy_* containing psg0906 |
| LCML245 | JE2 *Δspa:P_xyl/tetO3_-dcas9_Spy_* containing psg1072 (*mraZ*) |
| LCML246 | JE2 *Δspa:P_xyl/tetO3_-dcas9_Spy_* containing psg1108 (*def*) |
| LCML247 | JE2 *Δspa:P_xyl/tetO3_-dcas9_Spy_* containing psg1121 (*fapR*) |
| LCML248 | JE2 *Δspa:P_xyl/tetO3_-dcas9_Spy_* containing psg1155 (*rasP*) |
| LCML249 | JE2 *Δspa:P_xyl/tetO3_-dcas9_Spy_* containing psg1158 (*rimP*) |
| LCML250 | JE2 *Δspa:P_xyl/tetO3_-dcas9_Spy_* containing psg1558 (*mtnM*) |
| LCML251 | JE2 *Δspa:P_xyl/tetO3_-dcas9_Spy_* containing psg1635 (*mutM*) |
| LCML252 | JE2 *Δspa:P_xyl/tetO3_-dcas9_Spy_* containing psg1690 |
| LCML253 | JE2 *Δspa:P_xyl/tetO3_-dcas9_Spy_* containing psg1914 (*pmtR*) |
| LCML254 | JE2 *Δspa:P_xyl/tetO3_-dcas9_Spy_* containing psg2029 |
| LCML255 | JE2 *Δspa:P_xyl/tetO3_-dcas9_Spy_* containing psg2205 (*rpsJ*) |
| LCML256 | JE2 *Δspa:P_xyl/tetO3_-dcas9_Spy_* containing psg0014 (*gdpP*) |
| LCML257 | JE2 *Δspa:P_xyl/tetO3_-dcas9_Spy_* containing psg1603 (*rplU*) |
| LCML258 | JE2 *Δspa:P_xyl/tetO3_-dcas9_Spy_* containing psg2172 (*rplM*) |
| LCML259 | JE2 *Δspa:P_xyl/tetO3_-dcas9_Spy_* containing psg1476 (*accB*) |
| LCML260 | JE2 *Δspa:P_xyl/tetO3_-dcas9_Spy_* containing psg1700 (*murJ*) |
| LCML261 | JE2 *Δspa:P_xyl/tetO3_-dcas9_Spy_* containing psg1080 (*ftsZ*) |
| B Clones |  |
| LCML4_B | JE2 *Δspa:P_xyl/tetO3_-dcas9_Spy_* containing psg0009_B (*serS*) |
| LCML6_B | JE2 *Δspa:P_xyl/tetO3_-dcas9_Spy_* containing psg0020_B (*walR*) |
| LCML7_B | JE2 *Δspa:P_xyl/tetO3_-dcas9_Spy_* containing psg0024_B (*walJ*) |
| LCML8_B | JE2 *Δspa:P_xyl/tetO3_-dcas9_Spy_* containing psg0089_B |
| LCML9_B | JE2 *Δspa:P_xyl/tetO3_-dcas9_Spy_* containing psg0248_B (*tarF*) |
| LCML10_B | JE2 *Δspa:P_xyl/tetO3_-dcas9_Spy_* containing psg0249_B (*ispD*) |
| LCML15_B | JE2 *Δspa:P_xyl/tetO3_-dcas9_Spy_* containing psg0453_B |
| LCML24_B | JE2 *Δspa:P_xyl/tetO3_-dcas9_Spy_* containing psg0487_B (*tilS*) |
| LCML25_B | JE2 *Δspa:P_xyl/tetO3_-dcas9_Spy_* containing psg0492_B (*folP*) |
| LCML28_B | JE2 *Δspa:P_xyl/tetO3_-dcas9_Spy_* containing psg0514_B (*cysE*) |
| LCML31_B | JE2 *Δspa:P_xyl/tetO3_-dcas9_Spy_* containing psg0551_B (*folE2*) |
| LCML32_B | JE2 *Δspa:P_xyl/tetO3_-dcas9_Spy_* containing psg0570_B (*eutD*) |
| LCML33_B | JE2 *Δspa:P_xyl/tetO3_-dcas9_Spy_* containing psg0572_B (*mvaK1*) |
| LCML34_B | JE2 *Δspa:P_xyl/tetO3_-dcas9_Spy_* containing psg0596_B (*argS*) |
| LCML35_B | JE2 *Δspa:P_xyl/tetO3_-dcas9_Spy_* containing psg0623_B (*tagA*) |
| LCML36_B | JE2 *Δspa:P_xyl/tetO3_-dcas9_Spy_* containing psg0624_B (*tagH*) |
| LCML40_B | JE2 *Δspa:P_xyl/tetO3_-dcas9_Spy_* containing psg0703_B (*ltaS*) |
| LCML42_B | JE2 *Δspa:P_xyl/tetO3_-dcas9_Spy_* containing psg0722_B (*murB*) |
| LCML46_B | JE2 *Δspa:P_xyl/tetO3_-dcas9_Spy_* containing psg0743_B (*hprK*) |
| LCML55_B | JE2 *Δspa:P_xyl/tetO3_-dcas9_Spy_* containing psg0868_B (*spsB*) |
| LCML56_B | JE2 *Δspa:P_xyl/tetO3_-dcas9_Spy_* containing psg0885_B (*fabH*) |
| LCML62_B | JE2 *Δspa:P_xyl/tetO3_-dcas9_Spy_* containing psg0922_B |
| LCML65_B | JE2 *Δspa:P_xyl/tetO3_-dcas9_Spy_* containing psg0983_B (*ptsH*) |
| LCML66_B | JE2 *Δspa:P_xyl/tetO3_-dcas9_Spy_* containing psg0989_B (*rnjA*) |
| LCML67_B | JE2 *Δspa:P_xyl/tetO3_-dcas9_Spy_* containing psg0990_B (*rpoY*) |
| LCML68_B | JE2 *Δspa:P_xyl/tetO3_-dcas9_Spy_* containing psg0991_B (*def*) |
| LCML70_B | JE2 *Δspa:P_xyl/tetO3_-dcas9_Spy_* containing psg1024_B (*coaD*) |
| LCML92_B | JE2 *Δspa:P_xyl/tetO3_-dcas9_Spy_* containing psg1133_B (*trmD*) |
| LCML97_B | JE2 *Δspa:P_xyl/tetO3_-dcas9_Spy_* containing psg1152_B (*frr*) |
| LCML98_B | JE2 *Δspa:P_xyl/tetO3_-dcas9_Spy_* containing psg1153_B (*uppS*) |
| LCML100_B | JE2 *Δspa:P_xyl/tetO3_-dcas9_Spy_* containing psg1157_B (*polC*) |
| LCML103_B | JE2 *Δspa:P_xyl/tetO3_-dcas9_Spy_* containing psg1176_B (*pgsA)* |
| LCML107_B | JE2 *Δspa:P_xyl/tetO3_-dcas9_Spy_* containing psg1249_B (*plsY*) |
| LCML109_B | JE2 *Δspa:P_xyl/tetO3_-dcas9_Spy_* containing psg1257_B (*msrR*) |
| LCML115_B | JE2 *Δspa:P_xyl/tetO3_-dcas9_Spy_* containing psg1340_B (*recU*) |
| LCML116_B | JE2 *Δspa:P_xyl/tetO3_-dcas9_Spy_* containing psg1341_B (*pbp2*) |
| LCML117_B | JE2 *Δspa:P_xyl/tetO3_-dcas9_Spy_* containing psg1344_B (*dnaD*) |
| LCML118_B | JE2 *Δspa:P_xyl/tetO3_-dcas9_Spy_* containing psg1347_B (*birA*) |
| LCML119_B | JE2 *Δspa:P_xyl/tetO3_-dcas9_Spy_* containing psg1348_B (*papS*) |
| LCML120_B | JE2 *Δspa:P_xyl/tetO3_-dcas9_Spy_* containing psg1351_B |
| LCML121_B | JE2 *Δspa:P_xyl/tetO3_-dcas9_Spy_* containing psg1360_B (*ubiE*) |
| LCML129_B | JE2 *Δspa:P_xyl/tetO3_-dcas9_Spy_* containing psg1464_B (*bmfBB*) |
| LCML131_B | JE2 *Δspa:P_xyl/tetO3_-dcas9_Spy_* containing psg1475_B (*accC*) |
| LCML132_B | JE2 *Δspa:P_xyl/tetO3_-dcas9_Spy_* containing psg1490_B (*efp*) |
| LCML133_B | JE2 *Δspa:P_xyl/tetO3_-dcas9_Spy_* containing psg1492_B |
| LCML134_B | JE2 *Δspa:P_xyl/tetO3_-dcas9_Spy_* containing psg1520_B (*trmK*) |
| LCML136_B | JE2 *Δspa:P_xyl/tetO3_-dcas9_Spy_* containing psg1522_B (*dnaG*) |
| LCML137_B | JE2 *Δspa:P_xyl/tetO3_-dcas9_Spy_* containing psg1525_B (*glyS*) |
| LCML139_B | JE2 *Δspa:P_xyl/tetO3_-dcas9_Spy_* containing psg1540_B (*dnaK*) |
| LCML140_B | JE2 *Δspa:P_xyl/tetO3_-dcas9_Spy_* containing psg1541_B (*grpE*) |
| LCML141_B | JE2 *Δspa:P_xyl/tetO3_-dcas9_Spy_* containing psg1546_B (*holA*) |
| LCML143_B | JE2 *Δspa:P_xyl/tetO3_-dcas9_Spy_* containing psg1567_B (*greA*) |
| LCML144_B | JE2 *Δspa:P_xyl/tetO3_-dcas9_Spy_* containing psg1575_B (*alaS*) |
| LCML147_B | JE2 *Δspa:P_xyl/tetO3_-dcas9_Spy_* containing psg1589_B (*dtd*) |
| LCML148_B | JE2 *Δspa:P_xyl/tetO3_-dcas9_Spy_* containing psg1593_B (*secF*) |
| LCML151_B | JE2 *Δspa:P_xyl/tetO3_-dcas9_Spy_* containing psg1611_B (*valS*) |
| LCML152_B | JE2 *Δspa:P_xyl/tetO3_-dcas9_Spy_* containing psg1619_B (*hemA*) |
| LCML154_B | JE2 *Δspa:P_xyl/tetO3_-dcas9_Spy_* containing psg1624_B |
| LCML156_B | JE2 *Δspa:P_xyl/tetO3_-dcas9_Spy_* containing psg1629_B (*thrS*) |
| LCML157_B | JE2 *Δspa:P_xyl/tetO3_-dcas9_Spy_* containing psg1631_B (*dnaB*) |
| LCML158_B | JE2 *Δspa:P_xyl/tetO3_-dcas9_Spy_* containing psg1632_B (*nrdR*) |
| LCML159_B | JE2 *Δspa:P_xyl/tetO3_-dcas9_Spy_* containing psg1634_B (*coaE*) |
| LCML161_B | JE2 *Δspa:P_xyl/tetO3_-dcas9_Spy_* containing psg1647_B (*accD*) |
| LCML162_B | JE2 *Δspa:P_xyl/tetO3_-dcas9_Spy_* containing psg1649_B (*dnaE*) |
| LCML163_B | JE2 *Δspa:P_xyl/tetO3_-dcas9_Spy_* containing psg1657_B (*ackA*) |
| LCML165_B | JE2 *Δspa:P_xyl/tetO3_-dcas9_Spy_* containing psg1675_B (*tyrS*) |
| LCML168_B | JE2 *Δspa:P_xyl/tetO3_-dcas9_Spy_* containing psg1704_B (*leuS*) |
| LCML169_B | JE2 *Δspa:P_xyl/tetO3_-dcas9_Spy_* containing psg1729_B |
| LCML170_B | JE2 *Δspa:P_xyl/tetO3_-dcas9_Spy_* containing psg1730_B (*metK*) |
| LCML171_B | JE2 *Δspa:P_xyl/tetO3_-dcas9_Spy_* containing psg1748_B |
| LCML172_B | JE2 *Δspa:P_xyl/tetO3_-dcas9_Spy_* containing psg1749_B |
| LCML173_B | JE2 *Δspa:P_xyl/tetO3_-dcas9_Spy_* containing psg1869_B (*map_*) |
| LCML180_B | JE2 *Δspa:P_xyl/tetO3_-dcas9_Spy_* containing psg1894_B (*pncB*) |
| LCML181_B | JE2 *Δspa:P_xyl/tetO3_-dcas9_Spy_* containing psg1900_B (*ppaC*) |
| LCML182_B | JE2 *Δspa:P_xyl/tetO3_-dcas9_Spy_* containing psg1911_B (*pmtC*) |
| LCML184_B | JE2 *Δspa:P_xyl/tetO3_-dcas9_Spy_* containing psg1983_B (*groES*) |
| LCML185_B | JE2 *Δspa:P_xyl/tetO3_-dcas9_Spy_* containing psg1988_B (*hld*) |
| LCML188_B | JE2 *Δspa:P_xyl/tetO3_-dcas9_Spy_* containing psg2028_B (*acpS*) |
| LCML191_B | JE2 *Δspa:P_xyl/tetO3_-dcas9_Spy_* containing psg2055_B (*murA*) |
| LCML192_B | JE2 *Δspa:P_xyl/tetO3_-dcas9_Spy_* containing psg2062_B (*atpF*) |
| LCML198_B | JE2 *Δspa:P_xyl/tetO3_-dcas9_Spy_* containing psg2113_B (*dacA*) |
| LCML200_B | JE2 *Δspa:P_xyl/tetO3_-dcas9_Spy_* containing psg2184_B (*secY*) |
| LCML202_B | JE2 *Δspa:P_xyl/tetO3_-dcas9_Spy_* containing psg2238_B (*ureA*) |
| LCML203_B | JE2 *Δspa:P_xyl/tetO3_-dcas9_Spy_* containing psg2283_B (*rpiA*) |
| LCML208_B | JE2 *Δspa:P_xyl/tetO3_-dcas9_Spy_* containing psg0005_B (*gyrB*) |
| LCML209_B | JE2 *Δspa:P_xyl/tetO3_-dcas9_Spy_* containing psg0527_B (*rpoB*) |
| LCML211_B | JE2 *Δspa:P_xyl/tetO3_-dcas9_Spy_* containing psg0639_B |
| LCML212_B | JE2 *Δspa:P_xyl/tetO3_-dcas9_Spy_* containing psg1359_B |
| LCML213_B | JE2 *Δspa:P_xyl/tetO3_-dcas9_Spy_* containing psg1590_B (*relA*) |
| LCML214_B | JE2 *Δspa:P_xyl/tetO3_-dcas9_Spy_* containing psg1791_B (*cbf1*) |
| LCML219_B | JE2 *Δspa:P_xyl/tetO3_-dcas9_Spy_* containing psg0015_B (*rplI*) |
| LCML221_B | JE2 *Δspa:P_xyl/tetO3_-dcas9_Spy_* containing psg0479_B (*rplY*) |
| LCML222_B | JE2 *Δspa:P_xyl/tetO3_-dcas9_Spy_* containing psg0522_B (*rplK*) |
| LCML223_B | JE2 *Δspa:P_xyl/tetO3_-dcas9_Spy_* containing psg0524_B (*rplJ*) |
| LCML225_B | JE2 *Δspa:P_xyl/tetO3_-dcas9_Spy_* containing psg0990_B (*rpoY*) |
| LCML231_B | JE2 *Δspa:P_xyl/tetO3_-dcas9_Spy_* containing psg1166_B (*rpsO*) |
| LCML234_B | JE2 *Δspa:P_xyl/tetO3_-dcas9_Spy_* containing psg1545_B (*rpsT*) |
| LCML235_B | JE2 *Δspa:P_xyl/tetO3_-dcas9_Spy_* containing psg1601_B (*rmpA*) |
| LCML236_B | JE2 *Δspa:P_xyl/tetO3_-dcas9_Spy_* containing psg1625_B (*rplT*) |
| LCML237_B | JE2 *Δspa:P_xyl/tetO3_-dcas9_Spy_* containing psg1666_B (*rpsD*) |
| LCML239_B | JE2 *Δspa:P_xyl/tetO3_-dcas9_Spy_* containing psg2171_B (*rpsI)* |
| LCML240_B | JE2 *Δspa:P_xyl/tetO3_-dcas9_Spy_* containing psg2177_B (*rplQ*) |
| LCML241_B | JE2 *Δspa:P_xyl/tetO3_-dcas9_Spy_* containing psg0386_B (*xpt*) |
| LCML245_B | JE2 *Δspa:P_xyl/tetO3_-dcas9_Spy_* containing psg1072_B (*mraZ*) |
| LCML249_B | JE2 *Δspa:P_xyl/tetO3_-dcas9_Spy_* containing psg1158_B (*rimP*) |
| LCML254_B | JE2 *Δspa:P_xyl/tetO3_-dcas9_Spy_* containing psg2029_B |
| LCML256_B | JE2 *Δspa:P_xyl/tetO3_-dcas9_Spy_* containing psg0014_B (*gdpP*) |

# **Supplementary Table 4. Lisbon CRISPRi Mutant Library clone list**

This table lists the final strains included in the Lisbon CRISPRi Mutant Library (LCML). The library contains 261 strains, each carrying a plasmid-encoded sgRNA to target each of the selected essential genes/operons. The clone suffix (A or B) denotes which of the two sgRNAs designed to target each gene resulted in the greatest reduction in R (the ratio of the area under the curve (AUC) of growth curves obtained in the presence (expressing dCas9) versus the absence (not expressing dCas9) of anhydrotetracycline). Temp indicates the temperature (in °C) at which the indicated R value was obtained.

| **LCML**  **Number** | **Locus Tag** | **Name** | **Best**  **Clone** | **R Value** | **Temp** | **sgRNA Sequence** |
| --- | --- | --- | --- | --- | --- | --- |
| 1 | SAUSA300_0001 | sg0001_dnaA | A | 0.083 | 30 | ATACACAAAAATAACAGCCGgttttAGAGCTAGAAATAGCAAGTTAAAATAAGGC |
| 2 | SAUSA300_0002 | sg0002_dnaN | A | 0.027 | 30 | TCGTTTATATATAATTATATgttttAGAGCTAGAAATAGCAAGTTAAAATAAGGC |
| 3 | SAUSA300_0003 | sg0003 | A | 0.385 | 30 | ACAAATTTCATTTAAAATAGgttttAGAGCTAGAAATAGCAAGTTAAAATAAGGC |
| 4 | SAUSA300_0009 | sg0009_serS | A | 0.267 | 37 | CAAATAATTATCATTTATTAgttttAGAGCTAGAAATAGCAAGTTAAAATAAGGC |
| 5 | SAUSA300_0016 | sg0016_dnaB | A | 0.261 | 30 | TCATACATTCTATCCATGAAgttttAGAGCTAGAAATAGCAAGTTAAAATAAGGC |
| 6 | SAUSA300_0020 | sg0020_walR | A | 0.469 | 37 | ATGTTTGTGTAAAAAATCACgttttAGAGCTAGAAATAGCAAGTTAAAATAAGGC |
| 7 | SAUSA300_0024 | sg0024_walJ | A | 0.389 | 37 | TGTAACTTTAGTTCATCGACgttttAGAGCTAGAAATAGCAAGTTAAAATAAGGC |
| 8 | SAUSA300_0089 | sg0089 | A | 0.407 | 37 | GCCAAAATAAAAAATGGACGgttttAGAGCTAGAAATAGCAAGTTAAAATAAGGC |
| 9 | SAUSA300_0248 | sg0248_tarF | B | 0.401 | 30 | ATATAATTACAAAAACACGTgttttAGAGCTAGAAATAGCAAGTTAAAATAAGGC |
| 10 | SAUSA300_0249 | sg0249_ispD | B | 0.422 | 30 | TCTAATGTATGGATTAAAATgttttAGAGCTAGAAATAGCAAGTTAAAATAAGGC |
| 11 | SAUSA300_0363 | sg0363 | A | 0.278 | 30 | TGATTATAAGCAGTCATAATgttttAGAGCTAGAAATAGCAAGTTAAAATAAGGC |
| 12 | SAUSA300_0367 | sg0367_ssb | A | 0.111 | 30 | TTTATATTTGCACCTCCTTGgttttAGAGCTAGAAATAGCAAGTTAAAATAAGGC |
| 13 | SAUSA300_0388 | sg0388_guaB | A | 0.177 | 30 | GGTAAAATATCAGATTGTGCgttttAGAGCTAGAAATAGCAAGTTAAAATAAGGC |
| 14 | SAUSA300_0452 | sg0452_dnaX | A | 0.364 | 30 | GGAAATTTTACGATTCCGTGgttttAGAGCTAGAAATAGCAAGTTAAAATAAGGC |
| 15 | SAUSA300_0453 | sg0453 | B | 0.367 | 30 | GTTTGTTGTGCTTGTTGCTTgttttAGAGCTAGAAATAGCAAGTTAAAATAAGGC |
| 16 | SAUSA300_0459 | sg00459_tmk | A | 0.021 | 30 | GTTTCTCCTTTGAAAATAATgttttAGAGCTAGAAATAGCAAGTTAAAATAAGGC |
| 17 | SAUSA300_0461 | sg0461_holB | A | 0.239 | 30 | CCTTTTGCTTTTATATAAAAgttttAGAGCTAGAAATAGCAAGTTAAAATAAGGC |
| **LCML**  **Number** | **Locus Tag** | **Name** | **Best**  **Clone** | **R Value** | **Temp** | **sgRNA Sequence** |
| 18 | SAUSA300_0467 | sg0467_metS | A | 0.015 | 30 | AAGATTTCTATGCATTTCAAgttttAGAGCTAGAAATAGCAAGTTAAAATAAGGC |
| 19 | SAUSA300_0477 | sg0477_glmU | A | 0.223 | 30 | TTCAGCACCATGTCCTACGAgttttAGAGCTAGAAATAGCAAGTTAAAATAAGGC |
| 20 | SAUSA300_0478 | sg0478_prs | A | 0.476 | 30 | AGATTACATTAATATTACATgttttAGAGCTAGAAATAGCAAGTTAAAATAAGGC |
| 21 | SAUSA300_0480 | sg0480_pth | A | 0.492 | 30 | GACAAATTCATCATTGTCATgttttAGAGCTAGAAATAGCAAGTTAAAATAAGGC |
| 22 | SAUSA300_0484 | sg0484 | B | 0.032 | 30 | TTCTAGCCACTGCTTTAACGgttttAGAGCTAGAAATAGCAAGTTAAAATAAGGC |
| 23 | SAUSA300_0485 | sg0485_divIC | A | 0.294 | 30 | CAATATCATTGCGATGTTTTgttttAGAGCTAGAAATAGCAAGTTAAAATAAGGC |
| 24 | SAUSA300_0487 | sg0487_tilS | B | 0.083 | 30 | TAGAAACAGCGACAACAATAgttttAGAGCTAGAAATAGCAAGTTAAAATAAGGC |
| 25 | SAUSA300_0492 | sg0492_folP | B | 0.434 | 30 | GTCAGCACCTTCATCTATCAgttttAGAGCTAGAAATAGCAAGTTAAAATAAGGC |
| 26 | SAUSA300_0496 | sg0496_lysS | A | 0.216 | 30 | TAATCCTTTAGTATTCCAACgttttAGAGCTAGAAATAGCAAGTTAAAATAAGGC |
| 27 | SAUSA300_0513 | sg0513_gltX | A | 0.059 | 30 | TGAAGATACCCAGTTGGACTgttttAGAGCTAGAAATAGCAAGTTAAAATAAGGC |
| 28 | SAUSA300_0514 | sg0514_cysE | B | 0.625 | 30 | TCTAATGTTGAACGTGCCGCgttttAGAGCTAGAAATAGCAAGTTAAAATAAGGC |
| 29 | SAUSA300_0532 | sg0532_fusA | A | 0.006 | 30 | CTGAATAAATACGATAGATAgttttAGAGCTAGAAATAGCAAGTTAAAATAAGGC |
| 30 | SAUSA300_0533 | sg0533_tuf | A | 0.008 | 30 | GTGACCGATAGTACCGATATgttttAGAGCTAGAAATAGCAAGTTAAAATAAGGC |
| 31 | SAUSA300_0551 | sg0551_folE2 | B | 0.431 | 30 | GTCATTTCGTTCTTAGTAGTgttttAGAGCTAGAAATAGCAAGTTAAAATAAGGC |
| 32 | SAUSA300_0570 | sg0570_eutD | A | 0.486 | 30 | TAAATCTTATTAATCATTCAgttttAGAGCTAGAAATAGCAAGTTAAAATAAGGC |
| 33 | SAUSA300_0572 | sg0572_mvaK1 | B | 0.501 | 30 | CGAATAGTTCCCGCTCTCTAgttttAGAGCTAGAAATAGCAAGTTAAAATAAGGC |
| 34 | SAUSA300_0596 | sg0596_argS | B | 0.216 | 30 | GGAACTTCAATTTTAATATCgttttAGAGCTAGAAATAGCAAGTTAAAATAAGGC |
| 35 | SAUSA300_0623 | sg0623_tagA | B | 0.342 | 30 | CGTTGATTGATTTGCAAAAAgttttAGAGCTAGAAATAGCAAGTTAAAATAAGGC |
| 36 | SAUSA300_0624 | sg0624_tagH | B | 0.216 | 30 | AAATGTTTTGTTTTTATGTTgttttAGAGCTAGAAATAGCAAGTTAAAATAAGGC |
| 37 | SAUSA300_0625 | sg0625_tagG | A | 0.213 | 30 | AAACCATAATTTGCATAACAgttttAGAGCTAGAAATAGCAAGTTAAAATAAGGC |
| 38 | SAUSA300_0626 | sg0626_tagB | A | 0.347 | 30 | TGCGGTTTATCAATCACTTGgttttAGAGCTAGAAATAGCAAGTTAAAATAAGGC |
| 39 | SAUSA300_0628 | sg0628_tagD | A | 0.369 | 30 | TCCTCATATTGTCACATCATgttttAGAGCTAGAAATAGCAAGTTAAAATAAGGC |
| 40 | SAUSA300_0703 | sg0703_ltaS | A | 0.597 | 30 | CTTCAAGGTAATCGTTATTAgttttAGAGCTAGAAATAGCAAGTTAAAATAAGGC |
| 41 | SAUSA300_0715 | sg0715_nrdl | A | 0.405 | 30 | TAAAAAATGCTTTAACAACAgttttAGAGCTAGAAATAGCAAGTTAAAATAAGGC |
| 42 | SAUSA300_0722 | sg0722_murB | B | 0.157 | 30 | TGATATTAAAGACATGAGAAgttttAGAGCTAGAAATAGCAAGTTAAAATAAGGC |
| 43 | SAUSA300_0731 | sg0731_tagO | A | 0.460 | 30 | TTCGATATTGCAATAACAATgttttAGAGCTAGAAATAGCAAGTTAAAATAAGGC |
| **LCML**  **Number** | **Locus Tag** | **Name** | **Best**  **Clone** | **R Value** | **Temp** | **sgRNA Sequence** |
| 44 | SAUSA300_0737 | sg0737_secA | A | 0.365 | 30 | CCTTTAGCTAAAAAACTGTTgttttAGAGCTAGAAATAGCAAGTTAAAATAAGGC |
| 45 | SAUSA300_0738 | sg0738_prfB | A | 0.417 | 30 | TTTGGTTATCCCAAAAATTgttttAGAGCTAGAAATAGCAAGTTAAAATAAGGC |
| 46 | SAUSA300_0743 | sg0743_hprK | A | 0.550 | 30 | TATTGCTTGCTTAATTTACAgttttAGAGCTAGAAATAGCAAGTTAAAATAAGGC |
| 47 | SAUSA300_0749 | sg0749 | A | 0.416 | 30 | TTGTCCACCCGTACAACCGAgttttAGAGCTAGAAATAGCAAGTTAAAATAAGGC |
| 48 | SAUSA300_0756 | sg0756_gapA | A | 0.460 | 30 | TTCAAGTATTATCTTTGCTGgttttAGAGCTAGAAATAGCAAGTTAAAATAAGGC |
| 49 | SAUSA300_0761 | sg_0761 | A | 0.393 | 30 | CGTTAAATTAAGTAATGCTTgttttAGAGCTAGAAATAGCAAGTTAAAATAAGGC |
| 50 | SAUSA300_0765 | sg0765_smpB | A | 0.018 | 30 | CGATTTTCCGCTAATGTACCgttttAGAGCTAGAAATAGCAAGTTAAAATAAGGC |
| 51 | SAUSA300_0818 | sg0818_sufC | A | 0.423 | 30 | TATCCTCAATAGACACATGTgttttAGAGCTAGAAATAGCAAGTTAAAATAAGGC |
| 52 | SAUSA300_0835 | sg0835_dltA | A | 0.426 | 30 | TGTCTAACAGCAATGCTTTGgttttAGAGCTAGAAATAGCAAGTTAAAATAAGGC |
| 53 | SAUSA300_0858 | sg0858 | A | 0.165 | 30 | GTCTCAACAAACGCACCGTAgttttAGAGCTAGAAATAGCAAGTTAAAATAAGGC |
| 54 | SAUSA300_0865 | sg0865_pgi | A | 0.397 | 30 | TTTCAACAAACATTTCAAACgttttAGAGCTAGAAATAGCAAGTTAAAATAAGGC |
| 55 | SAUSA300_0868 | sg0868_spsB | A | 0.684 | 30 | CGCTCGCCATCTTTCAAAGTgttttAGAGCTAGAAATAGCAAGTTAAAATAAGGC |
| 56 | SAUSA300_0885 | sg0885_fabH | A | 0.554 | 30 | GCATTGTCAATAATCTTTTCgttttAGAGCTAGAAATAGCAAGTTAAAATAAGGC |
| 57 | SAUSA300_0897 | sg0897_trpS | A | 0.020 | 30 | TAGTAGGAATTCCACTAGGTgttttAGAGCTAGAAATAGCAAGTTAAAATAAGGC |
| 58 | SAUSA300_0898 | sg0898_spxA | A | 0.104 | 30 | TTACGGCAAGATGTGCAACTgttttAGAGCTAGAAATAGCAAGTTAAAATAAGGC |
| 59 | SAUSA300_0908 | sg0908_ppnK | A | 0.118 | 30 | GTTCATCATTTTATGCTTTAgttttAGAGCTAGAAATAGCAAGTTAAAATAAGGC |
| 60 | SAUSA300_0912 | sg0912_fabI | A | 0.423 | 30 | CCTTATAATAATTAATTTAAgttttAGAGCTAGAAATAGCAAGTTAAAATAAGGC |
| 61 | SAUSA300_0919 | sg0919_murE | A | 0.674 | 30 | ACTATTAAAAATAAAACATAgttttAGAGCTAGAAATAGCAAGTTAAAATAAGGC |
| 62 | SAUSA300_0922 | sg0922 | B | 0.597 | 30 | AAATAAGGTAAGATCAAACTgttttAGAGCTAGAAATAGCAAGTTAAAATAAGGC |
| 63 | SAUSA300_0944 | sg0944_menA | A | 0.470 | 30 | ACGGAAGCAGTTAATGTATGgttttAGAGCTAGAAATAGCAAGTTAAAATAAGGC |
| 64 | SAUSA300_0948 | sg0948_menB | A | 0.187 | 30 | GTAAACGCATTGCGTACTTCgttttAGAGCTAGAAATAGCAAGTTAAAATAAGGC |
| 65 | SAUSA300_0983 | sg0983_ptsH | B | 0.302 | 30 | AAATTCAATTCGTTTAAAACgttttAGAGCTAGAAATAGCAAGTTAAAATAAGGC |
| 66 | SAUSA300_0989 | sg0989_rnjA | B | 0.089 | 30 | ATCCCTAATAAGTTATCATCgttttAGAGCTAGAAATAGCAAGTTAAAATAAGGC |
| 67 | SAUSA300_0990 | sg0990_rpoY | B | 0.760 | 30 | GGATAAAAAGTTTGGTAGAAgttttAGAGCTAGAAATAGCAAGTTAAAATAAGGC |
| 68 | SAUSA300_0991 | sg0991_def | A | 0.910 | 30 | GCTGCTTTTTGACGCAAAGTgttttAGAGCTAGAAATAGCAAGTTAAAATAAGGC |
| 69 | SAUSA300_1013 | sg1013_ftsW | A | 0.075 | 30 | CGGATAATCAATAAACTTTGgttttAGAGCTAGAAATAGCAAGTTAAAATAAGGC |
| **LCML**  **Number** | **Locus Tag** | **Name** | **Best**  **Clone** | **R Value** | **Temp** | **sgRNA Sequence** |
| 70 | SAUSA300_1024 | sg1024_coaD | A | 0.648 | 37 | GTAATGGGGTCAAAACTACCgttttAGAGCTAGAAATAGCAAGTTAAAATAAGGC |
| 71 | SAUSA300_1026 | sg1026 | A | 0.155 | 30 | ACCGTTTGATCAAATTCAAAgttttAGAGCTAGAAATAGCAAGTTAAAATAAGGC |
| 72 | SAUSA300_1037 | sg1037_pheS | A | 0.032 | 30 | AACGCAGGTTTATCTTCATTgttttAGAGCTAGAAATAGCAAGTTAAAATAAGGC |
| 73 | SAUSA300_1044 | sg1044_trxA | A | 0.319 | 30 | ACCGGAGCGATCATTTTACAgttttAGAGCTAGAAATAGCAAGTTAAAATAAGGC |
| 74 | SAUSA300_1049 | sg1049_murI | A | 0.418 | 30 | CCAGAGTCTATTACACCTATgttttAGAGCTAGAAATAGCAAGTTAAAATAAGGC |
| 75 | SAUSA300_1074 | sg1074_ftsL | A | 0.009 | 30 | AAACTTGTTCGTCATATGGTgttttAGAGCTAGAAATAGCAAGTTAAAATAAGGC |
| 76 | SAUSA300_1075 | sg1075_pbpA | A | 0.019 | 30 | GTCCGAATAAACCAACAAGTgttttAGAGCTAGAAATAGCAAGTTAAAATAAGGC |
| 77 | SAUSA300_1076 | sg1076_mraY | A | 0.001 | 30 | TTTAATGTAGGTATTAAAACgttttAGAGCTAGAAATAGCAAGTTAAAATAAGGC |
| 78 | SAUSA300_1077 | sg1077_murD | A | 0.656 | 30 | AATAAAAATGTATTAGTTGTgttttAGAGCTAGAAATAGCAAGTTAAAATAAGGC |
| 79 | SAUSA300_1078 | sg1078_divIB | A | 0.095 | 30 | GGAATACATCTAAGAAAAGAgttttAGAGCTAGAAATAGCAAGTTAAAATAAGGC |
| 80 | SAUSA300_1079 | sg1079_ftsA | A | 0.038 | 30 | ATTTTTTATACCGCTCGTGTgttttAGAGCTAGAAATAGCAAGTTAAAATAAGGC |
| 81 | SAUSA300_1082 | sg1082 | A | 0.485 | 37 | TTTGTAACTGCAATCACGTTgttttAGAGCTAGAAATAGCAAGTTAAAATAAGGC |
| 82 | SAUSA300_1083 | sg1083_sepF | A | 0.269 | 30 | TTTACCTGTTGTTGTTTGTCgttttAGAGCTAGAAATAGCAAGTTAAAATAAGGC |
| 83 | SAUSA300_1087 | sg1087_ileS | A | 0.206 | 30 | CGCATTGGGAAATCTGTTTTgttttAGAGCTAGAAATAGCAAGTTAAAATAAGGC |
| 84 | SAUSA300_1102 | sg1102_gmk | A | 0.100 | 30 | GTACCTTTACCTACTCCAGAgttttAGAGCTAGAAATAGCAAGTTAAAATAAGGC |
| 85 | SAUSA300_1104 | sg1104_coaBC | A | 0.200 | 30 | TGCCGCAATGCCACCTGTAAgttttAGAGCTAGAAATAGCAAGTTAAAATAAGGC |
| 86 | SAUSA300_1105 | sg1105_priA | A | 0.181 | 30 | GATGACAGATTCGAGTTGTTgttttAGAGCTAGAAATAGCAAGTTAAAATAAGGC |
| 87 | SAUSA300_1109 | sg1109_fmt | A | 0.235 | 30 | AAAACAGTTGTTGAAAAGTCgttttAGAGCTAGAAATAGCAAGTTAAAATAAGGC |
| 88 | SAUSA300_1115 | sg1115_rpe | A | 0.097 | 30 | AAATCAACAGATAATAATGAgttttAGAGCTAGAAATAGCAAGTTAAAATAAGGC |
| 89 | SAUSA300_1122 | sg1122_plsX | A | 0.279 | 30 | GATATCGTATTAGAAGCCGTgttttAGAGCTAGAAATAGCAAGTTAAAATAAGGC |
| 90 | SAUSA300_1125 | sg1125_acpP | A | 0.224 | 30 | TATCAGCGTCTACACCTAAAgttttAGAGCTAGAAATAGCAAGTTAAAATAAGGC |
| 91 | SAUSA300_1128 | sg1128_ftsY | A | 0.189 | 30 | TTGACCTTGTTCTTCTGTTAgttttAGAGCTAGAAATAGCAAGTTAAAATAAGGC |
| 92 | SAUSA300_1133 | sg1133_trmD | B | 0.014 | 30 | TTTACAACATCAGCAATATAgttttAGAGCTAGAAATAGCAAGTTAAAATAAGGC |
| 93 | SAUSA300_1136 | sg1136_rbgA | A | 0.309 | 30 | TTGGCTTTCGCCATATGTCCgttttAGAGCTAGAAATAGCAAGTTAAAATAAGGC |
| 94 | SAUSA300_1143 | sg1143_topA | A | 0.324 | 30 | TCAATGGTTTTTGCTTTTGCgttttAGAGCTAGAAATAGCAAGTTAAAATAAGGC |
| 95 | SAUSA300_1150 | sg1150_tsf | A | 0.066 | 30 | AATACCTTTTTCACGTgttttAGAGCTAGAAATAGCAAGTTAAAATAAGGC |
| **LCML**  **Number** | **Locus Tag** | **Name** | **Best**  **Clone** | **R Value** | **Temp** | **sgRNA Sequence** |
| 96 | SAUSA300_1151 | sg1151_pyrH | A | 0.280 | 30 | GCAACACTTTTAATAATTACgttttAGAGCTAGAAATAGCAAGTTAAAATAAGGC |
| 97 | SAUSA300_1152 | sg1152_frr | B | 0.528 | 30 | GCTAATTGTTGTACAGGTGTgttttAGAGCTAGAAATAGCAAGTTAAAATAAGGC |
| 98 | SAUSA300_1153 | sg1153_uppS | B | 0.334 | 30 | TCGTAATGACCTTTAATTCTgttttAGAGCTAGAAATAGCAAGTTAAAATAAGGC |
| 99 | SAUSA300_1156 | sg1156_proS | A | 0.221 | 30 | CATCGTTGGTATAAAAACTTgttttAGAGCTAGAAATAGCAAGTTAAAATAAGGC |
| 100 | SAUSA300_1157 | sg1157_polC | B | 0.415 | 30 | AAAATTAAATGCTTTATATTgttttAGAGCTAGAAATAGCAAGTTAAAATAAGGC |
| 101 | SAUSA300_1159 | sg1159_nusA | A | 0.122 | 30 | GCATCAATTAATACTGCTCTgttttAGAGCTAGAAATAGCAAGTTAAAATAAGGC |
| 102 | SAUSA300_1168 | sg1168_rnjB | A | 0.312 | 30 | TGAATAGTGAGTTTATATATgttttAGAGCTAGAAATAGCAAGTTAAAATAAGGC |
| 103 | SAUSA300_1176 | sg1176_pgsA | B | 0.129 | 30 | AACCATCAACAAAATCGCTAgttttAGAGCTAGAAATAGCAAGTTAAAATAAGGC |
| 104 | SAUSA300_1200 | sg1200_glnR | A | 0.490 | 30 | TGATGCAATCAGACGAAATAgttttAGAGCTAGAAATAGCAAGTTAAAATAAGGC |
| 105 | SAUSA300_1237 | sg1237_lexA | A | 0.296 | 30 | CCAATTTCGCGAACACTAGGgttttAGAGCTAGAAATAGCAAGTTAAAATAAGGC |
| 106 | SAUSA300_1239 | sg1239_tkt | A | 0.279 | 30 | TTGAAGTAATCTTTAGATTGgttttAGAGCTAGAAATAGCAAGTTAAAATAAGGC |
| 107 | SAUSA300_1249 | sg1249_plsY | B | 0.334 | 30 | TATCATATTCATTTAAATTAgttttAGAGCTAGAAATAGCAAGTTAAAATAAGGC |
| 108 | SAUSA300_1250 | sg1250_parE | A | 0.987 | 30 | GTTGATCCAATATACATACCgttttAGAGCTAGAAATAGCAAGTTAAAATAAGGC |
| 109 | SAUSA300_1257 | sg1257_msrR | B | 0.161 | 30 | TTTCTTAATAAATCGTACTAgttttAGAGCTAGAAATAGCAAGTTAAAATAAGGC |
| 110 | SAUSA300_1269 | sg1269_femA | A | 0.297 | 30 | TTACAGATAGCATGCCATACgttttAGAGCTAGAAATAGCAAGTTAAAATAAGGC |
| 111 | SAUSA300_1270 | sg1270_femB | A | 0.121 | 30 | TTGTACAAAGTTGTCAAATTgttttAGAGCTAGAAATAGCAAGTTAAAATAAGGC |
| 112 | SAUSA300_1311 | sg1311_murG | A | 0.300 | 30 | TGTCCAACTGTTCCCCCTCCgttttAGAGCTAGAAATAGCAAGTTAAAATAAGGC |
| 113 | SAUSA300_1319 | sg1319_folA | A | 0.164 | 30 | CAAGTCATGTGCAACTAGAAgttttAGAGCTAGAAATAGCAAGTTAAAATAAGGC |
| 114 | SAUSA300_1320 | sg1320_thyA | A | 0.077 | 30 | ACTTTCTTTGTCGTTAATAGgttttAGAGCTAGAAATAGCAAGTTAAAATAAGGC |
| 115 | SAUSA300_1340 | sg1340_recU | B | 0.596 | 30 | AATACATTTACCGTTAATGTgttttAGAGCTAGAAATAGCAAGTTAAAATAAGGC |
| 116 | SAUSA300_1341 | sg1341_pbp2 | B | 0.119 | 30 | AATTTAGCTTCGGTAAAAGCgttttAGAGCTAGAAATAGCAAGTTAAAATAAGGC |
| 117 | SAUSA300_1344 | sg1344_dnaD | B | 0.173 | 30 | GATTTTAACAATGAACTTTAgttttAGAGCTAGAAATAGCAAGTTAAAATAAGGC |
| 118 | SAUSA300_1347 | sg1347_birA | B | 0.462 | 30 | ATACCTTGATACCAAATATCgttttAGAGCTAGAAATAGCAAGTTAAAATAAGGC |
| 119 | SAUSA300_1348 | sg1348_papS | B | 0.574 | 30 | CGAACAAATGTAACACCACTgttttAGAGCTAGAAATAGCAAGTTAAAATAAGGC |
| 120 | SAUSA300_1351 | sg1351 | A | 0.767 | 30 | CTTTGTGTTGTGCCCATAAgttttAGAGCTAGAAATAGCAAGTTAAAATAAGGC |
| 121 | SAUSA300_1360 | sg1360_ubiE | B | 0.500 | 30 | ATACCAGTAACTTCACCTGTgttttAGAGCTAGAAATAGCAAGTTAAAATAAGGC |
| **LCML**  **Number** | **Locus Tag** | **Name** | **Best**  **Clone** | **R Value** | **Temp** | **sgRNA Sequence** |
| 122 | SAUSA300_1362 | sg1362_hup | A | 0.042 | 30 | CATTAGACATTCACCTCCTGgttttAGAGCTAGAAATAGCAAGTTAAAATAAGGC |
| 123 | SAUSA300_1364 | sg1364_engA | A | 0.476 | 30 | ATTGTAGATTTACCTACATTgttttAGAGCTAGAAATAGCAAGTTAAAATAAGGC |
| 124 | SAUSA300_1367 | sg1367_cmk | A | 0.346 | 30 | ACCATCTAATGCAATATTAAgttttAGAGCTAGAAATAGCAAGTTAAAATAAGGC |
| 125 | SAUSA300_1373 | sg1373_fer | A | 0.613 | 30 | TCGTCGTAATCATATATATCgttttAGAGCTAGAAATAGCAAGTTAAAATAAGGC |
| 126 | SAUSA300_1453 | sg1453_rnz | A | 0.877 | 37 | TGTGTATTTCTCTCTTTTGTgttttAGAGCTAGAAATAGCAAGTTAAAATAAGGC |
| 127 | SAUSA300_1454 | sg1454_zwf | A | 0.259 | 30 | CCAAAGATTGTGATTAAACAgttttAGAGCTAGAAATAGCAAGTTAAAATAAGGC |
| 128 | SAUSA300_1459 | sg1459_gnd | A | 0.024 | 30 | AGCTAGGTTTTTACCCATAAgttttAGAGCTAGAAATAGCAAGTTAAAATAAGGC |
| 129 | SAUSA300_1464 | sg1464_bmfBB | B | 0.332 | 30 | ACAGGAGAAAATGGCATAGAgttttAGAGCTAGAAATAGCAAGTTAAAATAAGGC |
| 130 | SAUSA300_1466 | sg1466_bmfBAA | A | 0.026 | 30 | CTTTTAGGTCTTCTTCGCTAgttttAGAGCTAGAAATAGCAAGTTAAAATAAGGC |
| 131 | SAUSA300_1475 | sg1475_accC | B | 0.284 | 30 | TGCACATATGCTTCTTCGTCgttttAGAGCTAGAAATAGCAAGTTAAAATAAGGC |
| 132 | SAUSA300_1490 | sg1490_efp | A | 0.594 | 30 | CTTTACCAGGCTTTACATGTgttttAGAGCTAGAAATAGCAAGTTAAAATAAGGC |
| 133 | SAUSA300_1492 | sg1492 | B | 0.466 | 37 | AAATGCACAAACGTTTCACTgttttAGAGCTAGAAATAGCAAGTTAAAATAAGGC |
| 134 | SAUSA300_1520 | sg1520_trmK | A | 0.523 | 30 | ATTCTCCTTTATGAAAAAAGgttttAGAGCTAGAAATAGCAAGTTAAAATAAGGC |
| 135 | SAUSA300_1521 | sg1521_rpoD | A | 0.113 | 30 | CTGTGTTATCAGACATGAAAgttttAGAGCTAGAAATAGCAAGTTAAAATAAGGC |
| 136 | SAUSA300_1522 | sg1522_dnaG | A | 0.696 | 30 | ATTTATTTTGTTCAATATAAgttttAGAGCTAGAAATAGCAAGTTAAAATAAGGC |
| 137 | SAUSA300_1525 | sg1525_glyS | A | 0.143 | 37 | TCATACATGAAAACGCCCCAgttttAGAGCTAGAAATAGCAAGTTAAAATAAGGC |
| 138 | SAUSA300_1530 | sg1530_ybeY | A | 0.202 | 30 | TCAATGATCTTACTTACCAAgttttAGAGCTAGAAATAGCAAGTTAAAATAAGGC |
| 139 | SAUSA300_1540 | sg1540_dnaK | A | 0.726 | 30 | TCAGGGTTTTGAATTACTTTgttttAGAGCTAGAAATAGCAAGTTAAAATAAGGC |
| 140 | SAUSA300_1541 | sg1541_grpE | A | 0.805 | 30 | TCATTAATTTTTTGATCTTTgttttAGAGCTAGAAATAGCAAGTTAAAATAAGGC |
| 141 | SAUSA300_1546 | sg1546_holA | A | 0.815 | 30 | CTTTGTTTTTCAACCAATTCgttttAGAGCTAGAAATAGCAAGTTAAAATAAGGC |
| 142 | SAUSA300_1557 | sg1557 | A | 0.016 | 30 | ATTGATTGAACATATGAATTgttttAGAGCTAGAAATAGCAAGTTAAAATAAGGC |
| 143 | SAUSA300_1567 | sg1567_greA | A | 0.687 | 30 | TCAAAACCTTCTTGAGTCATgttttAGAGCTAGAAATAGCAAGTTAAAATAAGGC |
| 144 | SAUSA300_1575 | sg1575_alaS | A | 0.772 | 30 | ATTGGCACTAATGGTGCAGAgttttAGAGCTAGAAATAGCAAGTTAAAATAAGGC |
| 145 | SAUSA300_1579 | sg1579_iscS | A | 0.135 | 30 | TACTTCAGGTTTTACTGGTGgttttAGAGCTAGAAATAGCAAGTTAAAATAAGGC |
| 146 | SAUSA300_1587 | sg1587_hisS | A | 0.481 | 30 | AAAATATCCTGCGTCCCTCTgttttAGAGCTAGAAATAGCAAGTTAAAATAAGGC |
| 147 | SAUSA300_1589 | sg1589_dtd | A | 0.460 | 37 | GTATTTCACCATTCATTTGTgttttAGAGCTAGAAATAGCAAGTTAAAATAAGGC |
| **LCML**  **Number** | **Locus Tag** | **Name** | **Best**  **Clone** | **R Value** | **Temp** | **sgRNA Sequence** |
| 148 | SAUSA300_1593 | sg1593_secF | A | 0.763 | 30 | TTTATAAGTTGCAGCCATTCgttttAGAGCTAGAAATAGCAAGTTAAAATAAGGC |
| 149 | SAUSA300_1600 | sg1600_obgE | A | 0.045 | 30 | AATACCATTACCACCATCACgttttAGAGCTAGAAATAGCAAGTTAAAATAAGGC |
| 150 | SAUSA300_1602 | sg1602 | A | 0.205 | 30 | CATATTCACCATGGTCAGCAgttttAGAGCTAGAAATAGCAAGTTAAAATAAGGC |
| 151 | SAUSA300_1611 | sg1611_valS | B | 0.363 | 30 | TACCAGTTACATTTGGTGGCgttttAGAGCTAGAAATAGCAAGTTAAAATAAGGC |
| 152 | SAUSA300_1619 | sg1619_hemA | B | 0.185 | 30 | TACAAAAACTATGAAATATAgttttAGAGCTAGAAATAGCAAGTTAAAATAAGGC |
| 153 | SAUSA300_1620 | sg1620_engB | A | 0.093 | 30 | ATGATTAATTCTATATTATTgttttAGAGCTAGAAATAGCAAGTTAAAATAAGGC |
| 154 | SAUSA300_1624 | sg1624 | B | 0.395 | 30 | AAATGAGTTGTTTATATGAgttttAGAGCTAGAAATAGCAAGTTAAAATAAGGC |
| 155 | SAUSA300_1627 | sg1627_infC | A | 0.040 | 30 | TTGAGTTTGATCTTTTGCTAgttttAGAGCTAGAAATAGCAAGTTAAAATAAGGC |
| 156 | SAUSA300_1629 | sg1629_thrS | B | 0.703 | 30 | ATTGATCCATCAGTTTCAAGgttttAGAGCTAGAAATAGCAAGTTAAAATAAGGC |
| 157 | SAUSA300_1631 | sg1631_dnaB | A | 0.425 | 30 | TGGTCTTAAGCCGAATTCGAgttttAGAGCTAGAAATAGCAAGTTAAAATAAGGC |
| 158 | SAUSA300_1632 | sg1632_nrdR | B | 0.569 | 30 | CTAACTCCGAAGTCAGAGTTgttttAGAGCTAGAAATAGCAAGTTAAAATAAGGC |
| 159 | SAUSA300_1634 | sg1634_coaE | B | 0.501 | 30 | CTTCCACACACTTTGCATACgttttAGAGCTAGAAATAGCAAGTTAAAATAAGGC |
| 160 | SAUSA300_1645 | sg1645_pfkA | A | 0.311 | 30 | TGTACGAACAACTGCTCTTAgttttAGAGCTAGAAATAGCAAGTTAAAATAAGGC |
| 161 | SAUSA300_1647 | sg1647_accD | B | 0.332 | 30 | TGAACTTGACCAGTTAAAAAgttttAGAGCTAGAAATAGCAAGTTAAAATAAGGC |
| 162 | SAUSA300_1649 | sg1649_dnaE | B | 0.229 | 30 | ACTGAAACTCCTGACGCATTgttttAGAGCTAGAAATAGCAAGTTAAAATAAGGC |
| 163 | SAUSA300_1657 | sg1657_ackA | B | 0.652 | 30 | ACTATTACAGATTATTTTTTgttttAGAGCTAGAAATAGCAAGTTAAAATAAGGC |
| 164 | SAUSA300_1673 | sg1673_plsC | A | 0.054 | 30 | ACGACATATTTACTATCCTTgttttAGAGCTAGAAATAGCAAGTTAAAATAAGGC |
| 165 | SAUSA300_1675 | sg1675_tyrS | A | 0.485 | 37 | TAAACTATCTGCCGTTGGATgttttAGAGCTAGAAATAGCAAGTTAAAATAAGGC |
| 166 | SAUSA300_1686 | sg1686_murC | A | 0.116 | 30 | TTTATGTTATTAGCATCAAAgttttAGAGCTAGAAATAGCAAGTTAAAATAAGGC |
| 167 | SAUSA300_1688 | sg1688 | A | 0.152 | 30 | GCGACATCTCCTACATATTTgttttAGAGCTAGAAATAGCAAGTTAAAATAAGGC |
| 168 | SAUSA300_1704 | sg1704_leuS | A | 0.380 | 37 | ATTTCTTTTCAATTTGATTGgttttAGAGCTAGAAATAGCAAGTTAAAATAAGGC |
| 169 | SAUSA300_1729 | sg1729 | A | 0.624 | 30 | ATAATGAGTCCAATGACTATgttttAGAGCTAGAAATAGCAAGTTAAAATAAGGC |
| 170 | SAUSA300_1730 | sg1730_metK | B | 0.338 | 30 | TGTTGTTGTAGAAATTTCGCgttttAGAGCTAGAAATAGCAAGTTAAAATAAGGC |
| 171 | SAUSA300_1748 | sg1748 | B | 0.492 | 30 | AGTAGAGTCGCCTATCTCTCgttttAGAGCTAGAAATAGCAAGTTAAAATAAGGC |
| 172 | SAUSA300_1749 | sg1749 | B | 0.401 | 30 | GTTACAACCCATATGATTGTgttttAGAGCTAGAAATAGCAAGTTAAAATAAGGC |
| 173 | SAUSA300_1869 | sg1869_map | B | 0.247 | 30 | AAGATGTTACTTTAGTATTTgttttAGAGCTAGAAATAGCAAGTTAAAATAAGGC |
| **LCML**  **Number** | **Locus Tag** | **Name** | **Best**  **Clone** | **R Value** | **Temp** | **sgRNA Sequence** |
| 174 | SAUSA300_1873 | sg1873_murT | A | 0.241 | 30 | TACGCGCCAATTTCGCTAGAgttttAGAGCTAGAAATAGCAAGTTAAAATAAGGC |
| 175 | SAUSA300_1879 | sg1879_dgkB | A | 0.009 | 30 | ATAGCTCTTTACCTGATGTCgttttAGAGCTAGAAATAGCAAGTTAAAATAAGGC |
| 176 | SAUSA300_1881 | sg1881_gatA | A | 0.119 | 30 | ATATCTTTAACAACATCAGAgttttAGAGCTAGAAATAGCAAGTTAAAATAAGGC |
| 177 | SAUSA300_1882 | sg1882_gatC | A | 0.069 | 30 | GCCATTTCTTCCGTTTCTTCgttttAGAGCTAGAAATAGCAAGTTAAAATAAGGC |
| 178 | SAUSA300_1885 | sg1885_ligA | A | 0.252 | 30 | TATTCACTATCTGGTACAGAgttttAGAGCTAGAAATAGCAAGTTAAAATAAGGC |
| 179 | SAUSA300_1886 | sg1886_pcrA | A | 0.358 | 30 | GCACCTGCCATAATTAACAAgttttAGAGCTAGAAATAGCAAGTTAAAATAAGGC |
| 180 | SAUSA300_1894 | sg1894_pncB | B | 0.555 | 30 | GTAGACTATAATATAAAGCGgttttAGAGCTAGAAATAGCAAGTTAAAATAAGGC |
| 181 | SAUSA300_1900 | sg1900_ppaC | B | 0.515 | 30 | AATGAAGCCACTCCCTCAGCgttttAGAGCTAGAAATAGCAAGTTAAAATAAGGC |
| 182 | SAUSA300_1911 | sg1911_pmtC | B | 0.496 | 30 | AAAATATATGAATATAAATCgttttAGAGCTAGAAATAGCAAGTTAAAATAAGGC |
| 183 | SAUSA300_1913 | sg1913_pmtA | A | 0.496 | 30 | ATTAACATTACTTAATTCTAgttttAGAGCTAGAAATAGCAAGTTAAAATAAGGC |
| 184 | SAUSA300_1983 | sg1983_groES | A | 0.648 | 30 | ATAATCACACGATTTCCAATgttttAGAGCTAGAAATAGCAAGTTAAAATAAGGC |
| 185 | SAUSA300_1988 | sg1988_hld | B | 0.420 | 30 | AGTATTTATTTCCTACAGTTgttttAGAGCTAGAAATAGCAAGTTAAAATAAGGC |
| 186 | SAUSA300_2002 | sg2002_gcp | A | 0.199 | 30 | TGATGTCTACTTGCCACTTCgttttAGAGCTAGAAATAGCAAGTTAAAATAAGGC |
| 187 | SAUSA300_2005 | sg2005_tsaE | A | 0.195 | 30 | TTTAATGATGTTAAATGTCGgttttAGAGCTAGAAATAGCAAGTTAAAATAAGGC |
| 188 | SAUSA300_2028 | sg2028_acpS | B | 0.280 | 30 | CTTGACCACCCGCTGTATAAgttttAGAGCTAGAAATAGCAAGTTAAAATAAGGC |
| 189 | SAUSA300_2039 | sg2039_ddl | A | 0.066 | 30 | CTTTCTCCAATCACCATCATgttttAGAGCTAGAAATAGCAAGTTAAAATAAGGC |
| 190 | SAUSA300_2046 | sg2046_oxaA | A | 0.102 | 30 | ACCATAATACCTAAAAATAAgttttAGAGCTAGAAATAGCAAGTTAAAATAAGGC |
| 191 | SAUSA300_2055 | sg2055_murA | A | 0.509 | 30 | AATAAAGATGCTGTCAATATgttttAGAGCTAGAAATAGCAAGTTAAAATAAGGC |
| 192 | SAUSA300_2062 | sg2062_atpF | B | 0.278 | 30 | CTGAATTTATGCGATAGGCAgttttAGAGCTAGAAATAGCAAGTTAAAATAAGGC |
| 193 | SAUSA300_2072 | sg2072_prfA | A | 0.221 | 30 | CTGAATCATTTACAACATCTgttttAGAGCTAGAAATAGCAAGTTAAAATAAGGC |
| 194 | SAUSA300_2079 | sg2079_fbaA | A | 0.437 | 30 | ATTTCTTTCATTGAAACTAAgttttAGAGCTAGAAATAGCAAGTTAAAATAAGGC |
| 195 | SAUSA300_2081 | sg2081_pyrG | A | 0.229 | 30 | CCTGGGTCAACATTTAAGTAgttttAGAGCTAGAAATAGCAAGTTAAAATAAGGC |
| 196 | SAUSA300_2084 | sg2084_coaA | A | 0.523 | 30 | ATTCAGTTTTAAAAGTACGTgttttAGAGCTAGAAATAGCAAGTTAAAATAAGGC |
| 197 | SAUSA300_2104 | sg2104_glmS | A | 0.379 | 30 | ACCTTTTAATAATAATTCTTgttttAGAGCTAGAAATAGCAAGTTAAAATAAGGC |
| 198 | SAUSA300_2113 | sg2113_dacA | A | 0.321 | 37 | ACTGAGGTTTTGAAAAAAGTgttttAGAGCTAGAAATAGCAAGTTAAAATAAGGC |
| 199 | SAUSA300_2182 | sg2182_infA | A | 0.438 | 30 | AACGCAATGTTTAAAGTAGAgttttAGAGCTAGAAATAGCAAGTTAAAATAAGGC |
| **LCML**  **Number** | **Locus Tag** | **Name** | **Best**  **Clone** | **R Value** | **Temp** | **sgRNA Sequence** |
| 200 | SAUSA300_2184 | sg2184_secY | A | 0.135 | 37 | TGTTCTAAAGAAGTTCACAAgttttAGAGCTAGAAATAGCAAGTTAAAATAAGGC |
| 201 | SAUSA300_2214 | sg2214_femX | A | 0.244 | 30 | TAATAAATCTCCATTTGGGTgttttAGAGCTAGAAATAGCAAGTTAAAATAAGGC |
| 202 | SAUSA300_2238 | sg2238_ureA | B | 0.536 | 30 | ATCTAATCGAAAACAAATAGgttttAGAGCTAGAAATAGCAAGTTAAAATAAGGC |
| 203 | SAUSA300_2283 | sg2283_rpiA | B | 0.380 | 30 | ATTTTAAATAATACTCGTTAgttttAGAGCTAGAAATAGCAAGTTAAAATAAGGC |
| 204 | SAUSA300_2292 | sg2292_fni | A | 0.357 | 30 | TGAATGCATTGCGTCAGATTgttttAGAGCTAGAAATAGCAAGTTAAAATAAGGC |
| 205 | SAUSA300_2483 | sg2483_mvaA | A | 0.282 | 30 | ATTCTTATCTAAATTTTGCAgttttAGAGCTAGAAATAGCAAGTTAAAATAAGGC |
| 206 | SAUSA300_2646 | sg2646_trmE | A | 0.283 | 30 | CCAATTGCCCCTTCACCCATgttttAGAGCTAGAAATAGCAAGTTAAAATAAGGC |
| 207 | SAUSA300_2484 | sg2484_mvaS | A | 0.277 | 30 | GCCATGTCTACATAGTACTTgttttAGAGCTAGAAATAGCAAGTTAAAATAAGGC |
| 208 | SAUSA300_0005 | sg0005_gyrB | B | 0.352 | 30 | GTCGATCCTATATACATACCgttttAGAGCTAGAAATAGCAAGTTAAAATAAGGC |
| 209 | SAUSA300_0527 | sg0527_rpoB | B | 0.312 | 30 | ATAAAAAGACAAAAAGAAAAgttttAGAGCTAGAAATAGCAAGTTAAAATAAGGC |
| 210 | SAUSA300_0425 | sg0425_mspA | A | 0.497 | 30 | ATAGTAGATTCTGTACATAAgttttAGAGCTAGAAATAGCAAGTTAAAATAAGGC |
| 211 | SAUSA300_0639 | sg0639 | B | 0.697 | 30 | ATAAAACTGCCTTCAACAATgttttAGAGCTAGAAATAGCAAGTTAAAATAAGGC |
| 212 | SAUSA300_1359 | sg1359 | B | 0.483 | 30 | TACTCAGAATAACAAATGCTgttttAGAGCTAGAAATAGCAAGTTAAAATAAGGC |
| 213 | SAUSA300_1590 | sg1590_relA | B | 0.445 | 37 | TGTATGGTAATCCGTTTTTTgttttAGAGCTAGAAATAGCAAGTTAAAATAAGGC |
| 214 | SAUSA300_1791 | sg1791_cbf1 | B | 0.367 | 37 | TTAACATGTACAATTTCTTCgttttAGAGCTAGAAATAGCAAGTTAAAATAAGGC |
| 215 | SAUSA300_2183 | sg2183_adk | A | 0.310 | 30 | TGAGTTCCTTTACCTGCGCCgttttAGAGCTAGAAATAGCAAGTTAAAATAAGGC |
| 216 | SAUSA300_2185 | sg2185_rplO | A | 0.372 | 30 | TAACTCATGTAATTTCATTTgttttAGAGCTAGAAATAGCAAGTTAAAATAAGGC |
| 217 | SAUSA300_2647 | sg2647_rnpA | A | 0.385 | 30 | AATCTGCATTCTTTTTAATTgttttAGAGCTAGAAATAGCAAGTTAAAATAAGGC |
| 218 | SAUSA300_2648 | sg2648_rpmH | A | 0.314 | 30 | TTACTATGTTTACGTTTATTgttttAGAGCTAGAAATAGCAAGTTAAAATAAGGC |
| 219 | SAUSA300_0015 | sg0015_rplI | B | 0.602 | 30 | TTTAAGTTGTGTTGCCGCATgttttAGAGCTAGAAATAGCAAGTTAAAATAAGGC |
| 220 | SAUSA300_0366 | sg0366_rpsF | A | 0.347 | 30 | TTTATATTTGCACCTCCTTGgttttAGAGCTAGAAATAGCAAGTTAAAATAAGGC |
| 221 | SAUSA300_0479 | sg0479_rplY | A | 0.506 | 30 | AACGTGTTTGTTTACCTTGAgttttAGAGCTAGAAATAGCAAGTTAAAATAAGGC |
| 222 | SAUSA300_0522 | sg0522_rplK | B | 0.321 | 30 | GCTGGACCAACTGGTGGTGCgttttAGAGCTAGAAATAGCAAGTTAAAATAAGGC |
| 223 | SAUSA300_0524 | sg0524_rplJ | B | 0.314 | 30 | CTTCAGCTACTGTTAATCCAgttttAGAGCTAGAAATAGCAAGTTAAAATAAGGC |
| 224 | SAUSA300_0530 | sg0530_rpsL | A | 0.191 | 30 | CGTACTAATTGGTTAATAGTgttttAGAGCTAGAAATAGCAAGTTAAAATAAGGC |
| 225 | SAUSA300_0990 | sg0990_rpoY | A | 0.358 | 37 | TAACCCTAGTAAAATCGTATgttttAGAGCTAGAAATAGCAAGTTAAAATAAGGC |
| **LCML**  **Number** | **Locus Tag** | **Name** | **Best**  **Clone** | **R Value** | **Temp** | **sgRNA Sequence** |
| 226 | SAUSA300_1027 | sg1027_rpmF | A | 0.404 | 30 | GTTTTAGAAGTTCTTCTTTTgttttAGAGCTAGAAATAGCAAGTTAAAATAAGGC |
| 227 | SAUSA300_1117 | sg1117_rpmB | A | 0.497 | 30 | CAGTTTACTCAAAATATAATgttttAGAGCTAGAAATAGCAAGTTAAAATAAGGC |
| 228 | SAUSA300_1131 | sg1131_rpsP | A | 0.260 | 30 | GCTACTACGATACGATAGAAgttttAGAGCTAGAAATAGCAAGTTAAAATAAGGC |
| 229 | SAUSA300_1134 | sg1134_rplS | A | 0.443 | 30 | CCTCGACAAATATATAGCAGgttttAGAGCTAGAAATAGCAAGTTAAAATAAGGC |
| 230 | SAUSA300_1149 | sg1149_rpsB | A | 0.286 | 30 | CCATTATAAATTCCTCCTATgttttAGAGCTAGAAATAGCAAGTTAAAATAAGGC |
| 231 | SAUSA300_1166 | sg1166_rpsO | B | 0.425 | 30 | AGTACAGCGATTTGTACTTCgttttAGAGCTAGAAATAGCAAGTTAAAATAAGGC |
| 232 | SAUSA300_1511 | sg1511_rpmG | A | 0.368 | 30 | TAAAGTTACGTTTACGCGCAgttttAGAGCTAGAAATAGCAAGTTAAAATAAGGC |
| 233 | SAUSA300_1535 | sg1535_rpsU | A | 0.321 | 30 | TCCCTCCCTCCAAATATCAAgttttAGAGCTAGAAATAGCAAGTTAAAATAAGGC |
| 234 | SAUSA300_1545 | sg1545_rpsT | B | 0.171 | 37 | CTTTTAGGAGGTGACAGAAAgttttAGAGCTAGAAATAGCAAGTTAAAATAAGGC |
| 235 | SAUSA300_1601 | sg1601_rpmA | B | 0.236 | 37 | TTGTCGTCATAATTGATATCgttttAGAGCTAGAAATAGCAAGTTAAAATAAGGC |
| 236 | SAUSA300_1625 | sg1625_rplT | B | 0.326 | 37 | TAACACGTTTAGCTGCTCCGgttttAGAGCTAGAAATAGCAAGTTAAAATAAGGC |
| 237 | SAUSA300_1666 | sg1666_rpsD | A | 0.457 | 37 | CCATGTTGTCCTGGTGCGTAgttttAGAGCTAGAAATAGCAAGTTAAAATAAGGC |
| 238 | SAUSA300_2074 | sg2074_rpmE2 | A | 0.276 | 30 | CTCCTTTGCCCTGAACCATCgttttAGAGCTAGAAATAGCAAGTTAAAATAAGGC |
| 239 | SAUSA300_2171 | sg2171_rpsI | B | 0.577 | 30 | ACTGTGATGTTACCTTCACCgttttAGAGCTAGAAATAGCAAGTTAAAATAAGGC |
| 240 | SAUSA300_2177 | sg2177_rplQ | A | 0.839 | 30 | GAAAAGAAGATTGATAAAGGgttttAGAGCTAGAAATAGCAAGTTAAAATAAGGC |
| 241 | SAUSA300_0386 | sg0386_xpt | A | 0.899 | 30 | CCAAACTAAATAATAGTTTCgttttAGAGCTAGAAATAGCAAGTTAAAATAAGGC |
| 242 | SAUSA300_0529 | sg0529_rplGB | A | 0.355 | 30 | ATTTACATAAAAATAACAAGgttttAGAGCTAGAAATAGCAAGTTAAAATAAGGC |
| 243 | SAUSA300_0775 | sg0755_gapR | A | 0.417 | 30 | CAACTTTCAAAGTAGAAAAAgttttAGAGCTAGAAATAGCAAGTTAAAATAAGGC |
| 244 | SAUSA300_0906 | sg0906 | A | 0.438 | 30 | CTTACGAACACTATTTTTAAgttttAGAGCTAGAAATAGCAAGTTAAAATAAGGC |
| 245 | SAUSA300_1072 | sg1072_mraZ | B | 0.208 | 37 | ATTTAAGTCATAACGAAACTgttttAGAGCTAGAAATAGCAAGTTAAAATAAGGC |
| 246 | SAUSA300_1108 | sg1108_def | A | 0.479 | 30 | GTAATCCTAAATTTATTGTAgttttAGAGCTAGAAATAGCAAGTTAAAATAAGGC |
| 247 | SAUSA300_1121 | sg1121_fapR | A | 0.415 | 30 | AGTTCATGGTCTGTGATGAAgttttAGAGCTAGAAATAGCAAGTTAAAATAAGGC |
| 248 | SAUSA300_1155 | sg1155_rasP | A | 0.307 | 30 | CCCATACCGATCGCAAATTCgttttAGAGCTAGAAATAGCAAGTTAAAATAAGGC |
| 249 | SAUSA300_1158 | sg1158_rimP | B | 0.279 | 30 | TAAGACATTGAAAAGAAATAgttttAGAGCTAGAAATAGCAAGTTAAAATAAGGC |
| 250 | SAUSA300_1558 | sg1558_mtnN | A | 0.406 | 30 | TATTGTTACTTCTTCTTCCAgttttAGAGCTAGAAATAGCAAGTTAAAATAAGGC |
| 251 | SAUSA300_1635 | sg1635_mutM | A | 0.493 | 30 | CTTTTTACATGTTCTACTTCgttttAGAGCTAGAAATAGCAAGTTAAAATAAGGC |
| **LCML**  **Number** | **Locus Tag** | **Name** | **Best**  **Clone** | **R Value** | **Temp** | **sgRNA Sequence** |
| 252 | SAUSA300_1690 | sg1690 | A | 0.444 | 30 | GGTTCTATCACTCTACAATCgttttAGAGCTAGAAATAGCAAGTTAAAATAAGGC |
| 253 | SAUSA300_1914 | sg1914_pmtR | A | 0.357 | 30 | TGCTTAATCTGTTCATAAATgttttAGAGCTAGAAATAGCAAGTTAAAATAAGGC |
| 254 | SAUSA300_2029 | sg2029 | B | 0.637 | 30 | CGTATTTTAAGTTAATCGATgttttAGAGCTAGAAATAGCAAGTTAAAATAAGGC |
| 255 | SAUSA300_2205 | sg2205_rpsJ | A | 0.298 | 30 | CTGCTGATTGATCAATTACGgttttAGAGCTAGAAATAGCAAGTTAAAATAAGGC |
| 256 | SAUSA300_0014 | sg0014_gdpP | A | 0.532 | 30 | TATTAGTAAAGCTTTCTTAGgttttAGAGCTAGAAATAGCAAGTTAAAATAAGGC |
| 257 | SAUSA300_1603 | sg1603_rplU | A | 0.273 | 30 | TAATAAGTCACGCCATACATgttttAGAGCTAGAAATAGCAAGTTAAAATAAGGC |
| 258 | SAUSA300_2172 | sg2172_rplM | A | 0.453 | 30 | ATGATAAACGACCTAATGTTgttttAGAGCTAGAAATAGCAAGTTAAAATAAGGC |
| 259 | SAUSA300_1476 | sg1476_accB | A | 0.310 | 30 | TTCAAGCATGTTCATATTGCgttttAGAGCTAGAAATAGCAAGTTAAAATAAGGC |
| 260 | SAUSA300_1700 | sg1700_murJ | A | 0.027 | 30 | CTTGGTAATTAATATACTAAgttttAGAGCTAGAAATAGCAAGTTAAAATAAGGC |
| 261 | SAUSA300_1080 | sg1080_ftsZ | A | 0.004 | 30 | AAATTTCCTCCTAGTTTTAgttttAGAGCTAGAAATAGCAAGTTAAAATAAGGC |

# **Supplementary Table 5. Additional primers used in this study**

| **Number** | **Sequence** |
| --- | --- |
| 5846 | ACTAGTATTATACCTAGGACTGAGCTAGC |
| 3381 | caaGGAGGCGCCGCAGGAACTATGACCTAATTTTGATGG |
| 3382 | CATAGTTCCTGCGGCGCCTCCttgaaaacggtcggtgaagcgc |
| 3627 | GCTGCGCTgtcgacgctcatacatgcgcctatttatatc |
| 3628 | CTTCACCCATttcatagcctccttacacttactcg |
| 3629 | ggaggctatgaaATGGGTGAAGATAGTGAATTAATTAG |
| 3630 | tgcattCCATGGccatatgaaaatgaaatctag |
| 3714 | TACTcccgggGGAGGCGCCGCAGGAGTCACCTCCTAGCTGACTC |
| 3715 | cccgggAGTAAAGGAGAAGAACTTTTCACTGG |
| 5450 | AACTGCAGAAAAAAtaaggaggAAAAAAAATGGATAAGAAATACTCAA |
| 5451 | CCGACGTCGACTTAGTCACCTCCTAGCTGACTCA |
| 5452 | CCGACGTCGACttaTTTGTATAGTTCATCCA |
| 5602 | cgtggatccTCCTGCGGCGCCTCCAGTAAAGGAGAAGAACTTTTCACTG |
| 5603 | cgccgaattcttaTTTGTATAGTTCATCCATGCC |
| 5639 | CGCGCCCGGGCAACACATACTTGTACTTGCC |
| 5745 | cgagctcgaatccggtctccc |
| 5746 | gggagaccggattcgagctcg |
| 5837 | GCGGGAATTCGTCATATGTAGAGAGGTACC |
| 5838 | gcggGAATTCgtcatatgtagagaggtacctcgagcggcccaagcttaaaaaaatctcgccaacaagttgacgagataaacacggcattttgccttgttttagtagattctgtaattttcattacagagtactaaaacTCTAATTTACGATCAACAAAAtgcgaaagacacaatacacc |
| 5839 | GTACTAAAACGATTTCTTTAAACAATCTTTTTGCGAAAGACACAATACACC |
| 5840 | GTCTTTCGCAAAAAGATTGTTTAAAGAAATCGTTTTAGTACTCTGTAATGA |
| 5845 | GTTTTAGAGCTAGAAATAGCAAGTTAAAATAAGGC |
| 5846 | ACTAGTATTATACCTAGGACTGAGCTAGC |
| 5922 | AATTCTAAAGATCTCGCGGGATCCTTACTCGAgatccccgggcgagctcgaatccg |
| 5923 | ctctagagtcgacctgcagcc |
| 6013 | cgcgcccgggTTGACAGCTAGCTCAGTCCTAGGTATAATACTAGTgtcccattcggcttggttcgagttttagtactctgtaatga |
| 7268 | tctatcattgatagagtcccgggaaaaaaaaataaggaggaaaatggataagaaatactcaataggc |
| 7272 | attaatgcagcgctagctacggccgttagtcacctcctagctgac |
| 5849 | TTTGGAGATCCGTTTGGTGGGTTTTAGAGCTAGAAATAGCAAGTTAAAATAAGGC |
| 5887 | AAACCAAAACCACCACCAAAGTTTTAGAGCTAGAAATAGCAAGTTAAAATAAGGC |
| 5889 | ttccatcgaaccaagccgaaGTTTTAGAGCTAGAAATAGCAAGTTAAAATAAGGC |
| 5890 | tcccattcggcttggttcgaGTTTTAGAGCTAGAAATAGCAAGTTAAAATAAGGC |
| 6423 | CTTGGTAATTAATATACTAAGTTTTAGAGCTAGAAATAGCAAGTTAAAATAAGGC |
| 6424 | AAATTTCCTCCTAGTTTTATGTTTTAGAGCTAGAAATAGCAAGTTAAAATAAGGC |
| 6426 | TCATATATCTTTCCTCGTTCGTTTTAGAGCTAGAAATAGCAAGTTAAAATAAGGC |
| 7015 | ATATgaattcTTAAGACCCACTTTCACATTTAAG |
| 7016 | ATATgctagctaCGGCCGtaCCCGGGACTCTATCAATGATAGAGAGCTTATTTTAATTATACTCTATCA |
| 7268 | tctatcattgatagagtcccgggaaaaaaaaataaggaggaaaatggataagaaatactcaataggc |
| 7272 | attaatgcagcgctagctacggccgttagtcacctcctagctgac |
